# Cation–π Interactions Contribute to Substrate Recognition in γ‐Butyrobetaine Hydroxylase Catalysis

**DOI:** 10.1002/chem.201503761

**Published:** 2015-12-14

**Authors:** Jos J. A. G. Kamps, Amjad Khan, Hwanho Choi, Robert K. Lesniak, Jürgen Brem, Anna M. Rydzik, Michael A. McDonough, Christopher J. Schofield, Timothy D. W. Claridge, Jasmin Mecinović

**Affiliations:** ^1^ Institute for Molecules and MaterialsRadboud University NijmegenHeyendaalseweg 1356525 AJNijmegenThe Netherlands; ^2^ Chemistry Research LaboratoryDepartment of ChemistryUniversity of Oxford12 Mansfield RoadOxfordOX1 3TAUK

**Keywords:** cation–pi interactions, C−H oxidation, enzyme catalysis, molecular recognition, oxygenases

## Abstract

γ‐Butyrobetaine hydroxylase (BBOX) is a non‐heme Fe^II^‐ and 2‐oxoglutarate‐dependent oxygenase that catalyzes the stereoselective hydroxylation of an unactivated C−H bond of γ‐butyrobetaine (γBB) in the final step of carnitine biosynthesis. BBOX contains an aromatic cage for the recognition of the positively charged trimethylammonium group of the γBB substrate. Enzyme binding and kinetic analyses on substrate analogues with P and As substituting for N in the trimethylammonium group show that the analogues are good BBOX substrates, which follow the efficiency trend N^+^>P^+^>As^+^. The results reveal that an uncharged carbon analogue of γBB is not a BBOX substrate, thus highlighting the importance of the energetically favorable cation–π interactions in productive substrate recognition.

## Introduction

**Figure 1:**
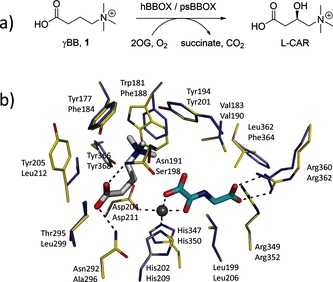
BBOX‐catalyzed hydroxylation: a) hBBOX‐/psBBOX‐catalyzed hydroxylation of γBB **1**; b) view of the hBBOX active site (yellow sticks, upper residue numbers, PDB ID: 3O2G) and the psBBOX model (blue sticks, lower residue numbers) with γBB (white sticks), *N*‐oxalylglycine (NOG, a 2OG analogue, cyan sticks) and Zn^II^ substituting for Fe^II^ (grey sphere).^13^

2‐Oxoglutarate (2OG) and Fe^II^‐dependent oxygenases play important roles in human physiology, including in hypoxia sensing, DNA repair, chromatin modification and fatty acid metabolism.^1^, ^2^ γ‐Butyrobetaine hydroxylase (BBOX), a 2OG oxygenase, catalyzes the stereoselective hydroxylation of γ‐butyrobetaine (γBB, **1**) to form l‐carnitine (l‐CAR) in eukaryotes and some prokaryotes (Figure 1 a).^3^, ^4^, ^5^, ^6^ l‐Carnitine is required for the transport of fatty acids into the mitochondrial matrix, where they are converted into acetyl‐CoA.^7^ Structural analyses on human BBOX (hBBOX) reveal that the active site Fe^II^ is chelated by a 2His‐1Asp triad and that the 2OG cosubstrate binds in a similar mode to other 2OG oxygenases (Figure 1 b).^8^, ^9^ The BBOX active site contains an apparent “aromatic cage,” which binds the γBB substrate's trimethylammonium group, and two asparagine residues that hydrogen bond with the γBB carboxylate (Figure 1 b).^8^, ^9^ The chiral environment of the enzyme's active site enables hBBOX to catalyze the oxidative desymmetrization of achiral *N*,*N*‐dialkyl piperidine‐4‐carboxylates.^10^ hBBOX also catalyzes an unusual Stevens‐type rearrangement of Mildronate (also known as Meldonium), a γBB competitive inhibitor that is clinically used in the treatment of myocardial infarction in order to inhibit fatty acid metabolism.^11^, ^12^ Recent studies revealed that subtle differences in the active sites of human and *Pseudomonas* sp. AK1 BBOX (hereafter psBBOX) can result in altered substrate‐analogue selectivities.^13^

Functionally and structurally diverse proteins contain aromatic cages (or aromatic boxes) as recognition modules for substrate binding. Aromatic cages typically comprise the sidechains of 2–4 aromatic residues (Trp, Tyr, Phe), and are observed on both exposed and buried sites.^14^, ^15^, ^16^ Work by Dougherty and coworkers has demonstrated that aromatic cages can recognize positively charged quaternary ammonium species *via* favorable cation–π interactions.^17^, ^18^, ^19^

Cation–π interactions are involved in associations of Cys‐loop receptors and G‐protein‐coupled receptors with neurotransmitters, including acetylcholine, serotonin, dopamine, epinephrine, and histamine.^20^ Studies on chromatin interactions involved in the regulation of gene expression reveal that “reader domain” proteins that recognize trimethyllysine‐containing histone tails interact through cation–π interactions.^14^, ^21^ Thus, along with hydrophobic effects, hydrogen bonding, ion pairing, and van der Waals interactions, cation–π interactions are crucial noncovalent forces in protein–protein and protein–ligand associations.^22^

**Scheme 1:**
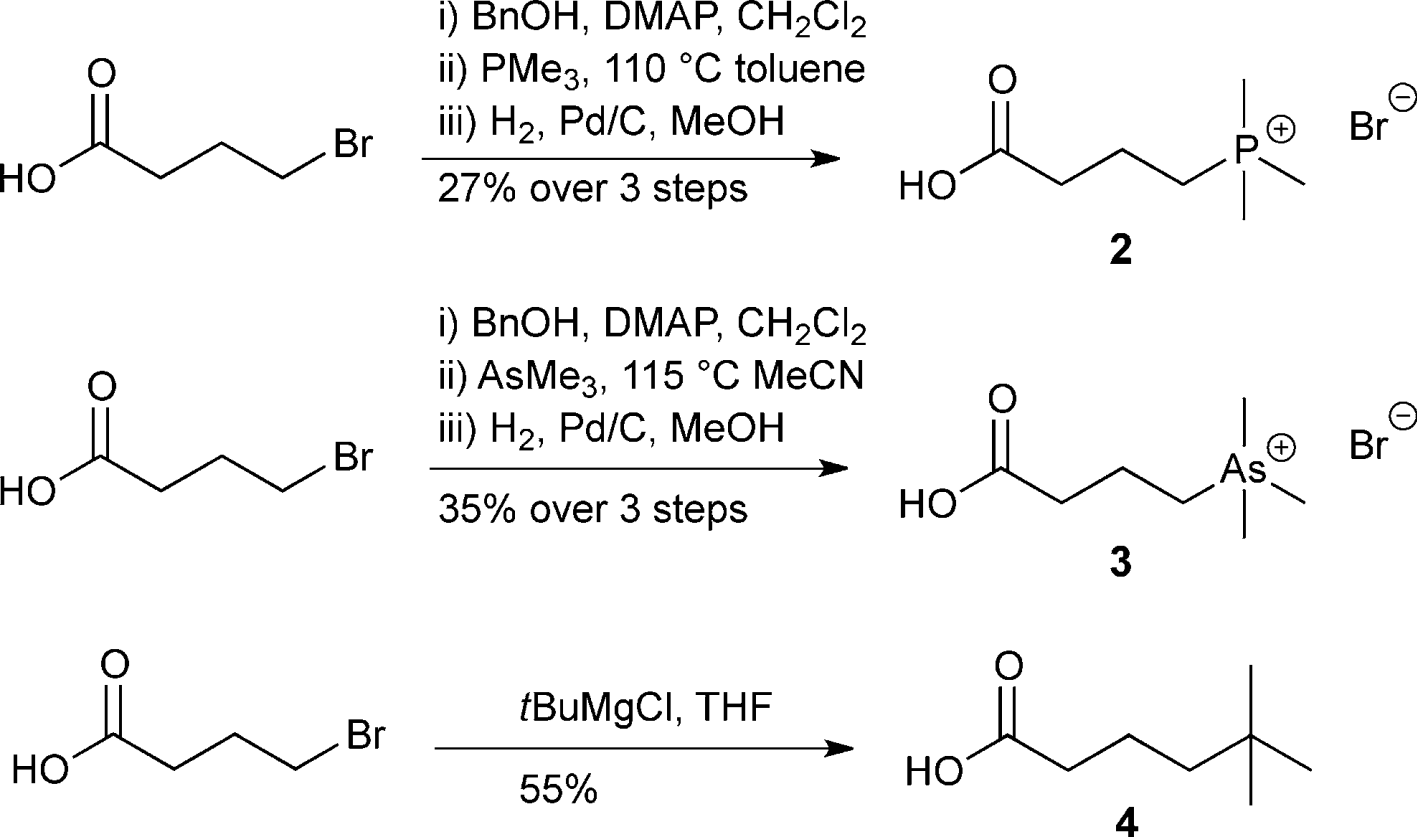
Syntheses of γBB analogues **2**–**4**. DMAP=4‐dimethylaminopyridine.

The BBOX aromatic cage contains the electron‐rich aromatic residues Phe184, Phe188 and Tyr201; Tyr368 is also located in close proximity, with its side‐chain OH group likely positioned to form an H‐bond with Asp211 (Figure 1 b).^13^ The location of the γBB trimethylammonium group inside the aromatic cage suggests that the association between γBB and BBOX could be substantially mediated by energetically favorable cation–π interactions. In support of this proposition, previous work with recombinant psBBOX employing a radioactive‐based assay with the C‐analogue **4** of γBB (**1**), provided evidence that it is a poor substrate.^23^ Herein, we report on the use of NMR and MS assays to investigate γBB analogues with P, As, and C substituting for N (**2**–**4**, respectively; Scheme 1) as psBBOX substrates; the results clearly support the proposal that cation–π interactions are crucial in the recognition of γBB by psBBOX.

We envisaged that replacement of the trimethylammonium group of γBB by three closely analogous functionalities, that is, positively charged trimethylphosphonium and trimethylarsonium “Group V analogues” **2** and **3**, and the neutral *tert*‐butyl analogue **4**, would provide insights into the role of cation–π interactions in BBOX catalysis (Scheme 1). Like γBB (**1**), the phosphorus (**2**) and arsenic (**3**) derivatives possess a “fixed” positive charge with respect to their trimethyl group, but are slightly larger than **1**, whereas the carbon analogue (**4**) has nearly the same size and shape, but lacks a positive charge. We hypothesized that direct comparison of binding and psBBOX‐catalyzed hydroxylation of the positively charged γBB **1** and neutral **4** would inform on the interactions between the NMe_3_^+^ group and the aromatic cage of psBBOX. Thus, if **4** is a much poorer psBBOX ligand and substrate than **1**, the requirement of cation–π interactions in psBBOX catalysis would be implied. In contrast, if **4** is a better ligand and substrate for psBBOX, that would suggest that hydrophobic interactions dominate the psBBOX–γBB association.

## Results and Discussion

The phosphorus (**2**) and arsenic (**3**) analogues of γBB (**1**) were synthesized in concise three‐step sequences from 4‐bromobutyric acid (Scheme 1 and Figure S1 in the Supporting Information). 5,5‐Dimethylhexanoic acid (**4**) was synthesized from 4‐bromobutanoic acid and a twofold excess of *tert*‐butylmagnesium chloride (Scheme 1 and Figure S1).

**Figure 2:**
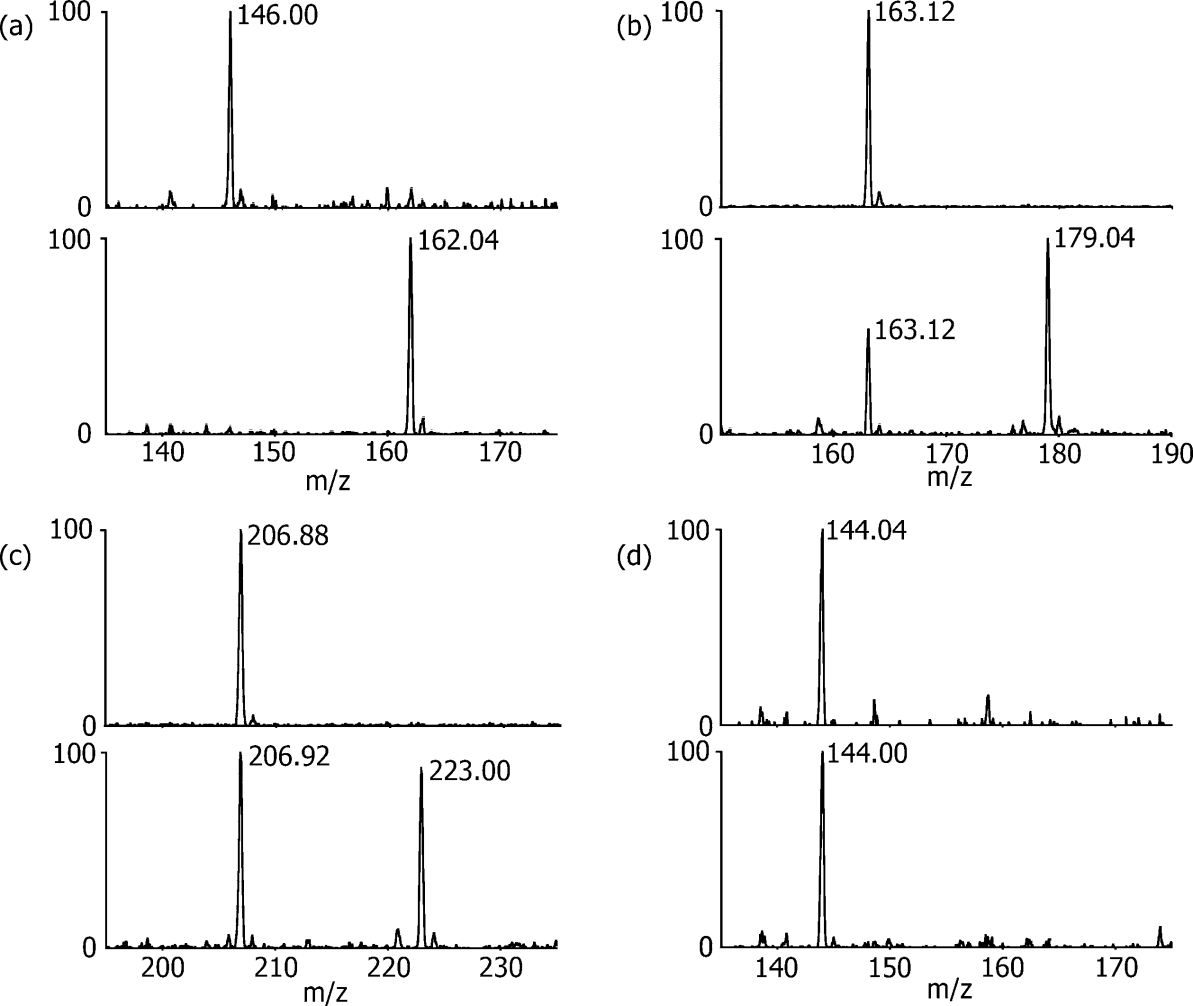
Mass spectrometry data for psBBOX‐catalyzed hydroxylations: a) natural substrate γ‐BB **1**; b) phosphorus analogue **2**; c) arsenic analogue **3**; d) neutral carbon analogue **4**. Top panel=starting substrate; bottom panel=psBBOX‐catalyzed reaction [a–c) 1 μm psBBOX, 5 min; d) 10 μm psBBOX, 3 h].

**Figure 3:**
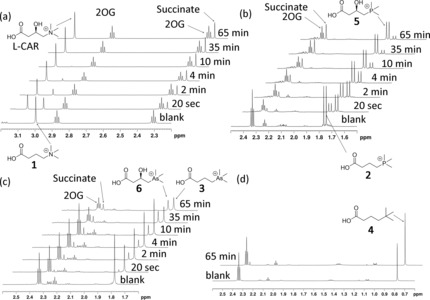
^1^H NMR monitoring of the hydroxylations by psBBOX in the presence of 2OG as a cosubstrate and Fe^II^ as a cofactor: a) **1**; b) **2**; c) **3**; d) **4** (blank=reaction mixture in the absence of psBBOX). The substrate/product ratio is measured for each spectrum as a function of reaction time.

We then used LC‐MS analyses to test for psBBOX‐catalyzed hydroxylation of the three γBB analogues. In the presence of psBBOX (1 μm), **1** was efficiently hydroxylated (complete conversion in 5 min; Figure 2 a and Figure S2 in the Supporting Information). Phosphorus analogue **2** was also a good substrate, with about 70 % conversion (Figure 2 b); arsenic analogue **3** was less well hydroxylated (approximately 45 %), but clear evidence for hydroxylation was obtained (Figure 2 c). Importantly, the neutral analogue **4** was not hydroxylated by psBBOX within our limits of detection; use of an increased amount of psBBOX (10 μm) and prolonged incubation (3 h) did not result in the observation of hydroxylated product by LC‐MS (Figure 2 d). In the absence of psBBOX, no hydroxylation of **2** or **3** took place (see the Supporting Information, Figure S3). Time‐course NMR studies were consistent with the order of efficiency as observed by LC‐MS (**1**>**2**>**3**) and revealed that in each case psBBOX‐catalyzed hydroxylation of **1**–**3** is tightly coupled to oxidation of 2OG to succinate (Figure 3 a–c). Consistent with the LC‐MS results, the NMR assays revealed that **4** was not hydroxylated, even in the presence of 10 μm psBBOX (Figure 3 d). Controls with **2** and **3** showed a lack of hydroxylation without psBBOX (see the Supporting Information, Figures S4 and S5). Overall, these results reveal the importance of a positively charged XMe_3_ substrate group for psBBOX catalysis.

**Scheme 2:**
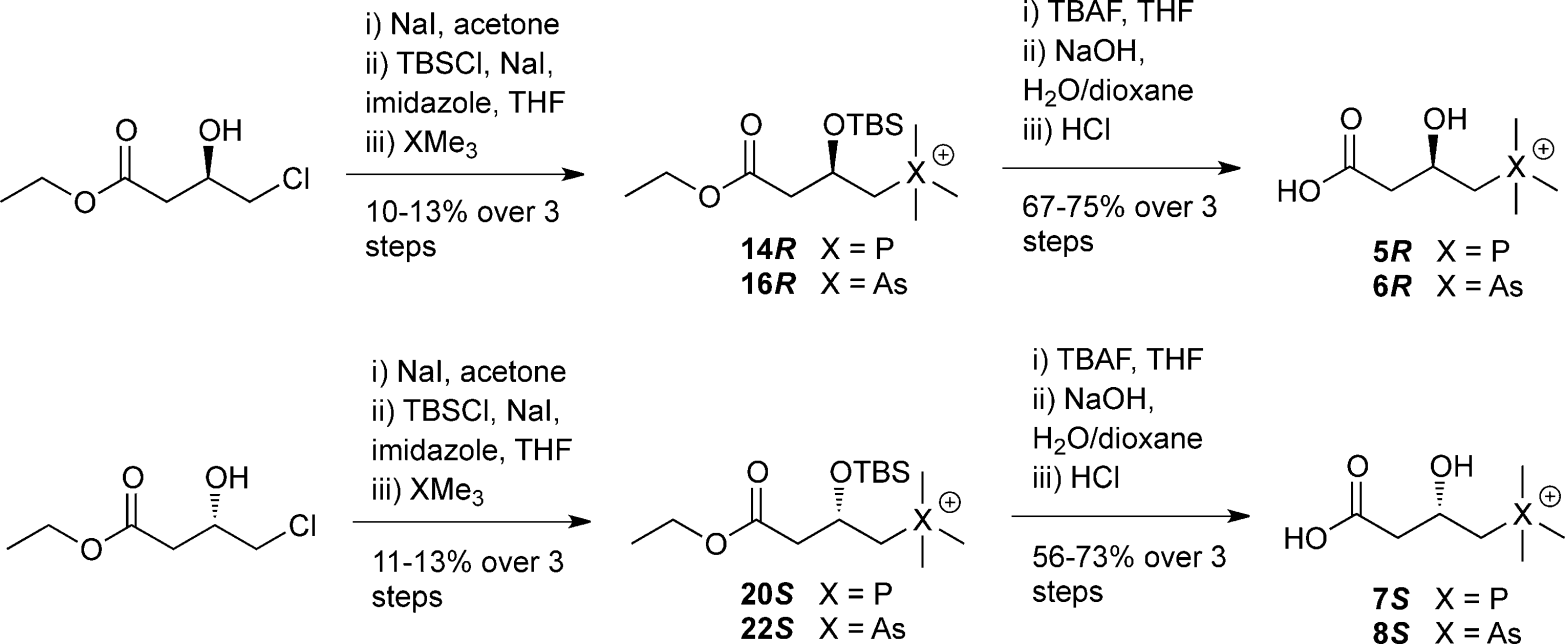
Stereoselective syntheses of (3*R*)‐ and (3*S*)‐hydroxylated phosphorus‐ and arsenic‐containing derivatives **5**–**8**.

**Scheme 3:**
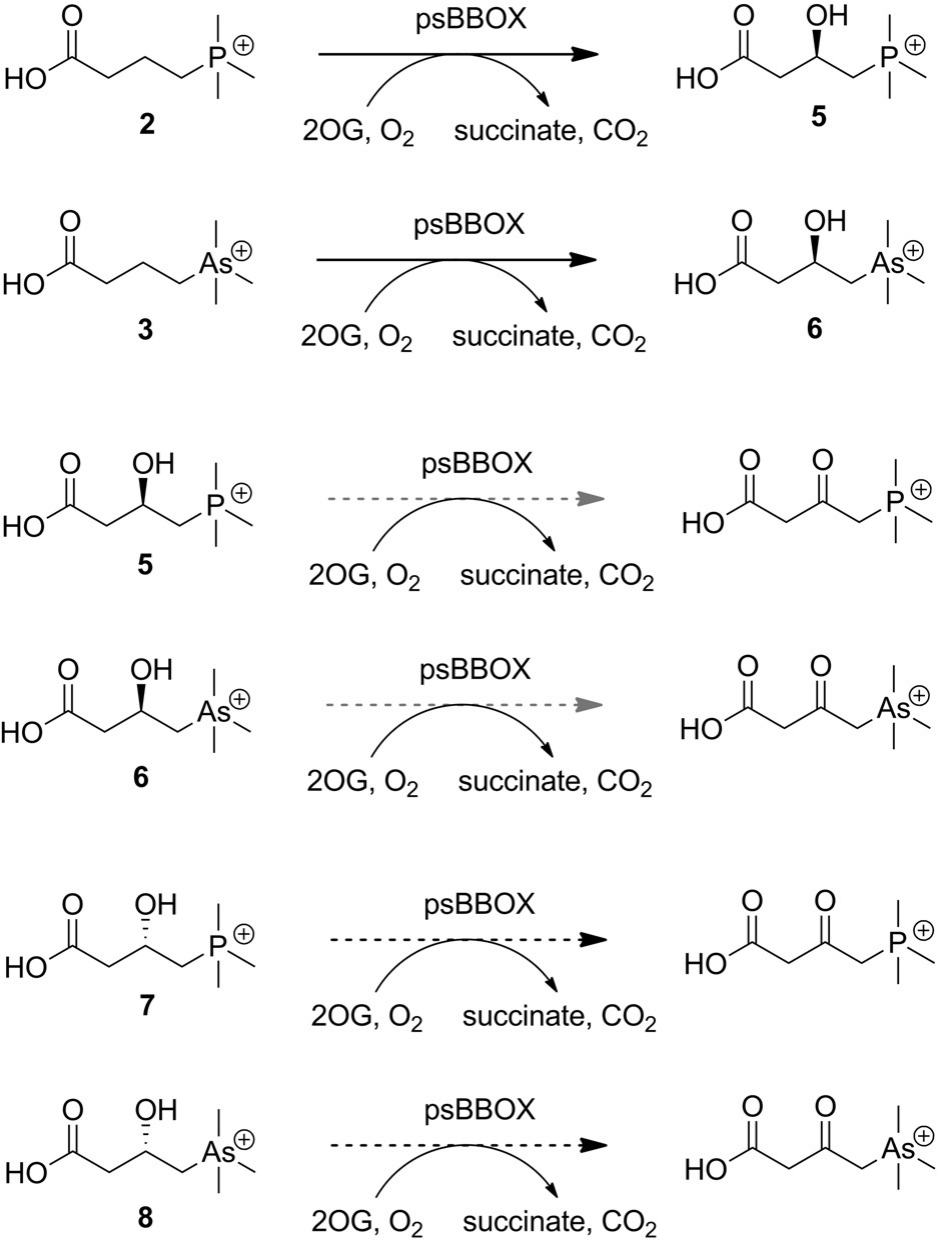
psBBOX‐catalyzed stereoselective hydroxylation of positively charged phosphorus (**2**) and arsenic (**3**) analogues of γBB, and their (3*R*)‐ and (3*S*)‐hydroxylated derivatives. Black dashed arrows and grey dashed arrows indicate poor and very poor conversions, respectively.

COSY‐ and HSQC‐based 2D NMR spectroscopy of the products implied that psBBOX‐catalyzed hydroxylation of **2** and **3** occurs at C3 (see the Supporting Information, Figures S6–S11). These assignments were confirmed by stereoselective synthesis of the (3*R*)‐ and (3*S*)‐hydroxylated phosphorus and arsenic derivatives **5**–**8** (Scheme 2 and Figure S12 in the Supporting Information). The intensity of ^1^H NMR peaks that correspond to the hydroxylated products increased upon the addition of authentic (3*R*)‐hydroxylated phosphorus‐ (**5**) and arsenic‐containing (**6**) products into the respective reaction mixtures, confirming that psBBOX‐catalyzed hydroxylation of the analogues occurs at C3 (see the Supporting Information, Figures S13 and S14). Together, these results imply that the psBBOX‐catalyzed hydroxylation of **2** and **3** results in the C3‐hydroxylated products **5** and **6**, likely with (3*R*) stereochemistry (Scheme 3).

LC‐MS analyses testing (3*R*)‐ and (3*S*)‐hydroxylated phosphorus derivatives **5** and **7** as substrates showed that the latter is a poor psBBOX substrate, giving a small amount of the 3‐keto product in the presence of 20 μm psBBOX (as assigned on the basis of a −2 Da mass shift), whereas the (3*R*)‐**5** enantiomer appears not to be converted within limits of detection for this assay (see the Supporting Information, Figure S15). We did not detect ketone formation with the hydroxylated arsenic derivatives (3*R*)‐**6** or (3*S*)‐**8** by LC‐MS (see the Supporting Information, Figure S16). However, NMR assays indicated that all four hydroxylated derivatives **5**–**8** are very poor psBBOX substrates with low levels of conversion being observed in the presence of 10 μm psBBOX, likely to the C3 ketone, which we could not fully characterize due to the low levels of conversion (Scheme 3 and Figures S17–S21 in the Supporting Information). The observation of a product with the same chemical shift for the PMe_3_^+^ group from incubation of **5** and **7** supports the proposed formation of the 3‐ketone (as with hydroxylation of the enantiomeric arsenic derivatives **6** and **8**; Figure S17–S21). As for the carnitine enantiomers,^13^ the (3*S*) enantiomers **7** and **8** were substantially better substrates than the (3*R*) enantiomers **5** and **6**, which afforded barely detectable products (36 % vs. 7 % for phosphorus derivatives **7** and **5**; 22 % vs. 3 % for arsenic derivatives **8** and **6**). These results are consistent with previous results of hBBOX‐ and psBBOX‐catalyzed hydroxylation of d‐ and l‐carnitine.^13^

Under (near) anaerobic conditions (Ar atmosphere), only traces (<5 %) of hydroxylated products were observed for **2** and **3** (see the Supporting Information, Figure S22). Reactions of **2** and **3** in H_2_^18^O (90 % ^18^O, Tris buffer, pH 7.5) afforded hydroxylated products with the same molecular mass as products from standard reaction in Tris buffer/H_2_^16^O (see the Supporting Information, Figure S23). These results are consistent with the oxygen atom in the hydroxylated products from **2** and **3** deriving (at least predominantly) from atmospheric oxygen and not from water, as also found in oxygen‐labeling studies on most other 2OG oxygenases.^24^

To quantify the relative efficiencies of the substrate analogues **2** and **3**, we carried out kinetic analyses using LC‐MS. The results revealed that the natural NMe_3_^+^‐containing substrate **1** is a superior substrate to the PMe_3_^+^‐ and AsMe_3_^+^‐containing analogues **2** and **3**, by approximately 2‐ and 3‐fold, respectively, as measured by *k*_cat_/*K*_m_ values (Table 1 and Figure S24 in the Supporting Information). The differences in *k*_cat_ and *K*_m_ values are relatively small for **1**, **2**, and **3**, implying the importance of a positively charged XMe_3_ group. There is a trend in decreasing *k*_cat_ from **1** to **3**, suggesting that the longer C−P and C−As bond lengths (1.9 Å and 2.0 Å, respectively) relative to the C−N bond length (1.5 Å) may cause substrates **2** and **3** to adopt a non‐optimal binding mode with respect to the Fe^IV^=O intermediate, as observed with substrate analogue studies on other 2OG oxygenases.^1^ The *K*_m_ values for all three positively charged substrates were within error (1.00–1.37 mm).

**Table 1:** Kinetic parameters for conversion of γBB **1** and the phosphorus (**2**) and arsenic (**3**) analogues into the corresponding hydroxylated products by psBBOX.^[a]^

	**1**	**2**	**3**
*V*_max_ [μm s^−1^]	2.81±0.31	1.65±0.16	1.04±0.13
*k*_cat_ [s^−1^]	7.02±0.77	4.13±0.40	2.61±0.31
*K*_m_ [mm]	1.00±0.21	1.20±0.20	1.37±0.28

[a] 400 nm psBBOX was used with varying concentrations of substrates from 50 μm to 1.5 mm.

Although the kinetic studies and time‐course analyses imply that binding in the aromatic cage is important, the small differences in the *k*_cat_ and *K*_m_ values and the complexity of 2OG oxygenase catalysis motivated us to carry out NMR titration studies to obtain *K*_D_ values for the binding of **1**–**3** to the psBBOX**⋅** Zn^II^ **⋅**2OG complex (using Zn^II^ as an unreactive Fe^II^ substitute); the *K*_D_ values for analogues **1**, **2**, and **3** were 5 μm, 7 μm, and 17 μm, respectively (see the Supporting Information, Figures S25–S27). The NMR experiments also implied that **4** does not bind in the active site of psBBOX within detection limits (see the Supporting Information, Figure S28). Similar trends have been observed in inhibition studies on the serine protease factor Xa, which contains an aromatic cage in its “S4” substrate residue binding pocket. That is, a ligand possessing a quaternary ammonium moiety inhibits factor Xa to a similar extent as one with an analogous phosphonium group, whereas the neutral carba analogue is substantially (60‐fold) less potent.^25^, ^26^

**Figure 4:**
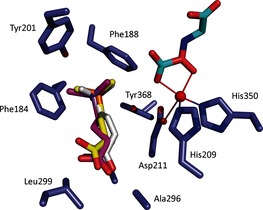
Modeled structure of psBBOX (blue) complexed with Zn^II^ (red), *N*‐oxalylglycine (cyan), and substrates **1** (white), **2** (yellow), and **3** (magenta).

To investigate the binding modes of **2** and **3** to psBBOX, we used an X‐ray crystal structure of hBBOX in complex with Zn^II^, NOG, and γBB [Protein Data Bank (PDB) ID: 3O2G] to make a homology model of psBBOX. All three positively charged substrates (**1**, **2**, **3**) are predicted to bind in a similar manner in the psBBOX active site, consistent with the observation of C3 hydroxylation, and with the XMe_3_^+^ group positioned in the aromatic cage (Figure 4). Due to strong negative inductive effect of a large XMe_3_^+^ group, the C4 position (adjacent to the XMe_3_^+^ group) is activated, but also the most sterically hindered. The C2 position, although the most activated, is positioned away from the Fe^IV^=O intermediate. Thus, of all three potential sites, psBBOX‐catalyzed hydroxylation occurs at the C3 site of **1**–**3**.

We calculated the CHELPG atomic charges (see Experimental Section) of the X atom of the XMe_3_ group and the attached three carbon and nine hydrogen atoms for the docked conformations for **1**–**3** and the minimized energy conformation for **4** (see the Supporting Information, Table S1). The average partial charges of the nine hydrogen atoms of XMe_3_^+^ show a very slight incremental trend of +0.1506 (for **3**), +0.1596 (for **2**), and +0.1652 (for **1**). The calculated partial charge of hydrogen atoms in the neutral *tert*‐butyl group of **4** is +0.0884. These results are in agreement with trends in binding affinities as observed by NMR spectroscopy, that is, the more positively charged H atoms of the XMe_3_^+^ substrates result in stronger cation–π interactions with the psBBOX aromatic cage.

## Conclusions

In conclusion, our substrate analogue studies employing both turnover and binding assays with purified recombinant enzyme clearly support the proposal that recognition and BBOX‐catalyzed hydroxylation of γBB involve energetically favorable cation–π interactions between the positively charged trimethylammonium group of γBB and the aromatic cage of psBBOX. The observation that the neutral carbon analogue of γBB does not bind to psBBOX and does not undergo psBBOX‐catalyzed hydroxylation in our assays further supports this view. Furthermore, the results reveal that the positively charged trimethylphosphonium and trimethylarsonium analogues of γBB are good substrate mimics and have a potential to act as small‐molecule probes for functional studies of carnitine biosynthesis. Thus, for example, the enzymatic conversion of the phosphorus analogue could be probed by ^31^P NMR spectroscopy, and the ^32^P‐labeled substrate might have a potential for utilization in radioactive tracing inside cells. Phosphorus and arsenic analogues of naturally occurring molecules that contain the quaternary ammonium groups might become useful probes for other genuinely important biomolecular processes that are driven by strong cation–π interactions. Given the central role of carnitine in eukaryotic fatty acid metabolism, our results also highlight biomedicinally important cation–π interactions.

## Experimental Section

### BBOX production and purification

Recombinant psBBOX was produced according to a previously described procedure.^13^ In brief, cells were cultured in 2TY media supplemented with 50 μg mL^−1^ ampicillin until mid‐log phase growth was achieved (OD600 0.7). Production of the recombinant proteins was then induced by addition of 0.2 mm IPTG and the cells were cultured for further 16 h at 15 °C. Cells were harvested by centrifugation (8 min, 8 g), then resuspended in lysis buffer (50 mm Tris pH 7.5/500 mm NaCl) supplemented with 0.2 % Tween 20, DNAse, Lysosyme, and EDTA‐free protease‐inhibitors.

The cell lysates were loaded onto a 5 mL HisTrap HP column (GE Healthcare Life Sciences, Little Chalfont, UK), with 50 mm Tris pH 7.5/500 mm NaCl, containing 20 mm imidazole, then eluted with an imidazole gradient (up to 500 mm imidazole). Fractions containing the purified psBBOX protein were concentrated by centrifugal ultrafiltration (50 kDa cutoff membrane). The protein solution was then injected onto a Superdex S200 column (300 mL) and eluted with 20 mm Tris pH 7.5/200 mm NaCl supplemented with 10 mm EDTA. Fractions containing purified psBBOX were concentrated by centrifugal ultrafiltration (50 kDa cutoff filter) and buffer exchanged by using a PD‐10 column to a Chelex 100‐treated metal‐free buffer (50 mm Tris pH 7.5/200 mm NaCl). The purity of the resulting fractions was ascertained to be >90 % by SDS‐PAGE analysis. Concentrations of the purified proteins were determined by using a ND‐1000 NanoDrop spectrophotometer.

### Enzyme kinetics experiments

Kinetics experiments were conducted at 296 K in Tris buffer (20 mm) and NaCl (200 mm) at pH 7.5. To a premixed solution of psBBOX (400 nm), FeSO_4_ (50 μm), 2OG (1.5 mm), and ascorbate (5 mm) was added the substrate in a range of different concentrations. After 1 min, an aliquot (20 μL) of the reaction mixture was quenched with MeCN (80 μL). Subsequently the sample was analyzed by LC‐MS. Each experiment was performed in duplicate.

### NMR experiments

All NMR experiments were performed on a Bruker Avance III 700 MHz spectrometer equipped with a TCI inverse cryoprobe and on a Bruker Avance II 500 MHz spectrometer equipped with a 5 mm ^13^C(^1^H) dual cryoprobe at 298 K and data analyzed using Bruker Topspin 3.2. All spectra were processed with a Lorentzian line broadening of 0.3 Hz. The solutions were buffered in 50 mm Tris‐D_11_ **⋅**HCl pH 7.5, in 90:10 H_2_O/D_2_O. Bruker MATCH 3 mm diameter and 5 mm NMR tubes, with total sample volumes of 160 μL and 500 μL, respectively, were used. For the psBBOX‐catalyzed substrate‐turnover experiments, the assay mixture was incubated in an Eppendorf tube and whenever necessary the reaction was quenched (stopped) with the addition of 1 m HCl (5 μL) and the spectrum was recorded for analysis. To measure the ligand binding constant by psBBOX titration, separate samples were prepared. The PROJECT‐CPMG pulse sequence (90°_*x*_–[*τ*–180°_*y*_–*τ*–90°_*y*_–*τ*–180°_*y*_–*τ*] *n*–acquisition), as described by Aguilar et al.,^27^ was used to remove the broad resonances of the protein. The relaxation edited (CPMG) ^1^H NMR experiments were recorded with a total filter time of 32 ms. Protein titration data were fitted using OriginPro 9.0 (Origin lab, Northampton, MA, USA) to calculate the ligand binding constant (*K*_D_). Water suppression was achieved by presaturation.

### Computational methods

For psBBOX protein‐ligand docking simulation, the X‐ray crystal structure of hBBOX in complex with NOG and γBB was employed (PDB entry: 3O2G) to make a homology structure of psBBOX using Modeller 9v4.^28^ Careful attention was paid to the assignment of protonation states for Asp, Glu, His, and Lys residues. We calculated partial charge distribution by using quantum mechanical calculation at PBE1PBE/LanL2DZ level of theory.^29^ Their partial charge is assigned with charges from electrostatic potentials using a grid‐based method (CHELPG).^30^ After reassigning CHELPG partial charges to X–γBB using the above quantum mechanical calculation, docking simulations of the X–γBB substrate with psBBOX were carried using the empirical AutoDock^31^ scoring function improved by implementation of a new solvation model. The modified scoring function has the following form [Eq. (1)]:

(1) $$\Delta G_{\mathrm{bind}}^{\mathrm{aq}}=W_{\mathrm{vdW}}\sum_{i=1}\sum_{j>1}\left(\frac{A_{ij}}{r_{ij}^{12}}-\frac{B_{ij}}{r_{ij}^{6}}\right)+W_{\mathrm{hbond}}\sum_{i=1}\sum_{j>1}E\left(t\right)\left(\frac{C_{ij}}{r_{ij}^{12}}-\frac{D_{ij}}{r_{ij}^{10}}\right)+W_{\mathrm{elec}}\sum_{i=1}\sum_{j>1}\frac{q_{1}q_{2}}{ɛ\left(r_{ij}\right)r_{ij}}+W_{\mathrm{tor}}N_{\mathrm{tor}}+\Delta G_{\mathrm{sol}}=\sum_{i}^{\mathrm{atoms}}\left\{S_{i}\left(O_{i}^{\max}-\sum_{j}^{i\neq j}V_{j}e^{\frac{r_{ij}^{2}}{2\sigma^{2}}}\right)+P_{i}\sum_{j}^{i\neq j}V_{j}e^{\frac{r_{ij}^{2}}{2\sigma^{2}}}\right\}$$

where *W*_vdW_, *W*_hbond_, *W*_elec_, *W*_tor_, and *W*_sol_ are the weighting factors for the van der Waals, hydrogen‐bond and electrostatic interactions, the torsional term, and the desolvation energy of the inhibitors, respectively. *r_ij_* represents the interatomic distance and *A_ij_*, *B_ij_*, *C_ij_*, and *D_ij_* are related to the depths of the potential energy well and the equilibrium separations between the two atoms. The hydrogen bond term has an additional weighting factor, *E*(*t*), representing the angle‐dependent directionality. A cubic equation approach was applied to obtain the dielectric constant required to compute the interatomic electrostatic interactions between psBBOX and X–γBB. In the entropic term, *N*_tor_ is the number of sp^3^ bonds in the ligand. In the desolvation term, *S_i_*, *P_i_*, and *V_i_* are the solvation parameter, self‐solvation parameter, and fragmental volume of atom *i*, respectively, whereas Occmaxi is the maximum atomic occupancy.^32^, ^33^

## Supporting information

As a service to our authors and readers, this journal provides supporting information supplied by the authors. Such materials are peer reviewed and may be re‐organized for online delivery, but are not copy‐edited or typeset. Technical support issues arising from supporting information (other than missing files) should be addressed to the authors.

Supplementary
