# Supplementary material for: Cation–π Interactions Contribute to Substrate Recognition in γ‐Butyrobetaine Hydroxylase Catalysis
Source: Chemistry. 2015 Dec 14;22(4):1270–6. doi: 10.1002/chem.201503761 (PMC4736438; doi:10.1002/chem.201503761)
Supplement: Supplementary file 1 — Supplementary [file CHEM-22-1270-s001.pdf]

CHEMISTRY
A European Journal

Supporting Information

Cation–π Interactions Contribute to Substrate Recognition in γ-Butyrobetaine Hydroxylase Catalysis

Jos J. A. G. Kamps,[a] Amjad Khan,[b] Hwanho Choi,[b] Robert K. Lesniak,[b] Jürgen Brem,[b] Anna M. Rydzik,[b] Michael A. McDonough,[b] Christopher J. Schofield,[b] Timothy D. W. Claridge,[b] and Jasmin Mecinović*[a]

chem_201503761_sm_miscellaneous_information.pdf

Table of contents

1. Synthetic procedures

2. Supporting figures

3. NMR spectra

4. References

1. Synthetic procedures

General methods

All experiments were conducted under the following conditions, unless otherwise stated: Commercially available compounds were used as supplied without purification. Dried solvents were obtained by purification of HPLC grade solvents over activated alumina column using an MBraun SPS800 solvent purification system. Compound purification was done by column chromatography, using silica gel, Merck™ grade (pore size 60 Å; particle size 230-400 mesh, 40-63 µm). Reaction progress was monitored using glass TLC plates (TLC Silica gel 60G, F_{254}, Merck, Germany) and observed by UV light and/ or by staining in permanganate. Compound analysis done by NMR (^{1}H, ^{13}C NMR analyses used), were recorded on a Varion Inova 400 at 400 MHz and 101 MHz respectively, while ^{31}P NMR analysis were done on either a Bruker DMX300 or Bruker Avance III 400 MHz at 121 MHz and 162 MHz respectively. Reported chemical shifts are in parts per million (ppm), moving from high to low frequencies and referenced to the residual solvent resonance ($CDCl_3$ or DMSO-d_6). Reported coupling constants (*J*) are noted in hertz (Hz) to the nearest 0.5 Hz. To assign multiplicity of signals the follow standard abbreviations were used: s = singlet, d = doublet, t = triplet, q = quartet, quint = quintet, m = multiplet, br = broad. When possible, ^{1}H assignments were made using appropriate 2D NMR methods, such as COSY, HSQC and HMBC. High resolution mass spectrometry (MS) analyses were performed using Electrospray Ionization MS using a JEOL AccuToF machine. Mass spectrometry and chromatography analysis were done using a Shimadzu UFLC LC-20AD LC/MS system, equipped with a RPC18 200 × 2 guard column. Typical conditions for an LC-MS run are: 157 bar, mobile phase; 2 minutes 5% MeCN 95% H_2O (both with 0.1% formic acid), in 16 minutes decreasing polarity to 100% MeCN, maintaining this polarity for 5 minutes. Subsequently the polarity is increased to 95% H_2O for 5 minutes. UV/VIS detection of this machine was done by Ultraviolet Visible Shimadzu SPD-M20A (200-600 nm), while MS analyses have been done using the Thermo scientific LCQ Fleet. When specified, purification of products was carried out by preparative HPLC using a Shimadzu LC 20AT, equipped with a Phenomenex Gemini NX (particle size: 10 µm, pore size: 110 Å, C18) in conjunction with a Shimadzu SPD 20A deuterium lamp at 215 and 254 nm as detector. A typical run was performed as follows: initial grade 10% MeCN in H_2O (both solvent contain 0.1% TFA), 10 mL/min, at 18 minutes polarity has decreased to 70% MeCN and further to 100% MeCN at 20 minutes which was maintained for 3 minutes. Afterwards the polarity was increased to 10% MeCN in H_2O over 2 minutes maintaining a constant polarity for 5 minutes (30 minutes total runtime).

Synthesis of 5,5-dimethylhexanoic acid (4)[1]

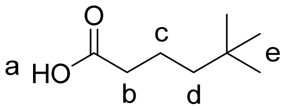

A solution of 4-bromobutanoic acid (501 mg, 3.70 mmol, 1 equivalent) in THF (8 mL) was purged with Ar $_{(g)}$ and then cooled at 0 °C, before carefully adding *tert*-butyl-magnesium chloride (8.2 mmol, 8.2 mL). After stirring for 18 hours the reaction was quenched with $NH_4Cl_{(aq)}$ (25 mL, saturated) and extracted with Et_2O (3 × 30 mL). The combined organic extracts were washed once with brine (50 mL) and dried over Na_2SO_4, filtered and concentrated. The crude product was purified by column chromatography (SiO_2, Et_2O in *n*-heptane 25 to 40%), affording compound **4** (291 mg, 2.02 mmol, 55%) as a clear colorless oil, which solidified upon standing. Mp : 35-36 °C. ^{1}H NMR (400 MHz, $CDCl_3$) δ: 11.34 (brs, 1H, H_a), 2.33 (t, J = 7.5 Hz, 2H, H_b), 1.66 – 1.55 (m, 2H, H_c), 1.25 – 1.18 (m, 2H, H_d), 0.89 (s, 9H, H_e). ^{13}C NMR (101 MHz, $CDCl_3$) δ: 180.5, 43.5, 34.9, 30.3, 29.2, 20.0.[1]

Synthesis of benzyl 4-bromobutanoate (9)

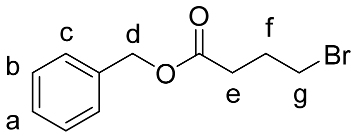

To a stirred solution of 4-bromobutyric acid (100 mg, 0.74 mmol, 1 equivalent) in dry CH_2Cl_2 (10 mL) were added DMAP (24 mg, 0.20 mmol, 0.27 equivalents), benzyl alcohol (170 µL, 1.51 mmol, 2.0 equivalents) and DCC (168 mg, 0.82 mmol, 1.1 equivalents) under inert atmosphere. The resulting solution was stirred for 3 hours, after which the solution was diluted with CH_2Cl_2 (20 mL) and washed with aqueous HCl (2 × 0.5 N in 20 mL). Subsequently the organic layer was washed with saturated $NaHCO_{3(aq)}$ (2 × 20 mL) and brine (30 mL) and dried over Na_2SO_4, filtered and concentrated. The crude product was purified by column chromatography (SiO_2, EtOAc in *n*-heptane 0 to 5%), affording benzyl ester **9** (70 mg, 0.27 mmol, 37%) as a clear colorless oil. ^{1}H NMR (400 MHz, $CDCl_3$) δ: 7.41 – 7.30 (m, 5H, H_a and H_b and H_c), 5.13 (s, 2H, H_d), 3.46 (t, J = 6.5 Hz, 2H, H_g), 2.56 (t, J = 7.5 Hz, 2H, H_e), 2.24 – 2.15 (m, 2H, H_f). ^{13}C NMR (101 MHz, $CDCl_3$) δ: 172.3, 135.7, 128.6, 128.3, 128.2, 66.4, 32.6, 32.4, 27.7.

Synthesis of (4-(benzyloxy)-4-oxobutyl) trimethylphosphonium bromide (10)

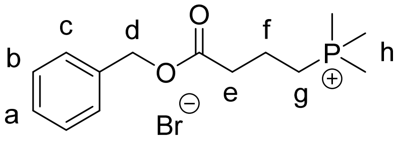

A microwave vial was charged with benzyl 4-bromobutarate ester **9** (500 mg, 1.94 mmol, 1 equivalent) under $Ar_{(g)}$ atmosphere. Subsequently a solution of PMe_3 (4.0 mL, 2 equivalents, 1 M in toluene) was added and the resulting solution was stirred for 60 minutes in a microwave machine applying a maximum of 400 W. The sample was then allowed to cool to r.t. and reaction mixture was diluted with MeOH and transferred into a round bottom flask. The solvent was removed and the crude solid was dissolved in a minimum amount of CH_2Cl_2 and precrystallized from Et_2O, affording **10** as a white solid (530 mg, 1.59 mmol, 82%). Mp: 143-145 °C. ^{1}H NMR (400 MHz, CD_3OD) δ: 7.40 – 7.24 (m, 5H, H_a and H_b and H_c), 5.13 (s, 2H, H_d), 2.57 (t, J = 7.0 Hz, 2H, H_e), 2.32 – 2.20 (m, 2H, H_g), 1.94 – 1.81 (m, 11H, H_f and H_h). ^{13}C NMR (101 MHz, CD_3OD) δ: 172.2, 136.1, 128.3, 128.1, 128.0, 66.1, 33.7 (d, J_{C-P} = 22.0 Hz), 22.2 (d, J_{C-P} = 53.0 Hz), 16.7 (d, J_{C-P} = 4.0 Hz), 6.8 (d, J_{C-P} =

55.0 Hz). ^{31}P NMR (121 MHz, CD_3OD) δ: 27.58 (m, 1P). ESI-MS calcd for $C_{14}H_{22}O_2P$ $[M]^+$ 253.1357, found 253.1336.

Synthesis of 4-trimethylphosphoniobutanoic acid bromide (2)

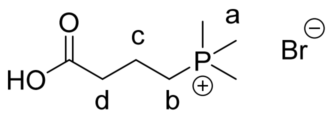

Bromide **10** (250 mg, 0.748 mmol, 1 equivalent) was dissolved in MeOH (20 mL) and treated with Pd/C (125 mg, 10% Pd/C). The suspension was stirred under $H_{2(g)}$ for 20 hours and subsequently filtered through a pad of Celite. After removal of the solvent, the crude product was recrystallised from $CHCl_3$, affording compound **2** (164 mg, 0.675 mmol, 90%) as clear colorless solids. Mp : 164-166 °C. ^{1}H NMR (400 MHz, D_2O) δ: 2.38 (t, J = 7.0 Hz, 2H, H_d), 2.13 – 2.03 (m, 2H, H_b), 1.78 – 1.66 (m, 11H, H_a and H_c). ^{13}C NMR (101 MHz, D_2O) δ: 177.1, 34.0 (d, $J_{C\text{-}P}$ = 21.5 Hz), 22.1 (d, $J_{C\text{-}P}$ = 53.5 Hz), 16.5 (d, $J_{C\text{-}P}$ = 4.0 Hz), 6.9 (d, $J_{C\text{-}P}$ = 55.5 Hz). ^{31}P NMR (121 MHz, D_2O) δ: 26.3 (m, 1P). ESI-MS calcd for $C_7H_{16}O_2P$ $[M]^+$ 163.0888, found 163.0885.

Synthesis of (4-(benzyloxy)-4-oxobutyl) trimethylarsonium bromide (11)

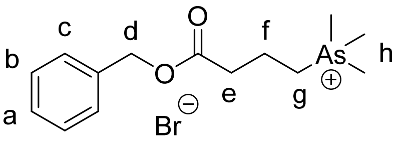

To a solution of bromine **9** (200 mg, 0.778 mmol, 1 equivalent) in MeCN (10 mL) was added $AsMe_3$ (180 µL, 1.69 mmol, 2.17 equivalents) under $Ar_{(g)}$ atmosphere. The reaction mixture was then heated to 115 °C and stirred under pressure for 22 hours. Subsequently the solution was allowed to cool to r.t. and the solvent was removed under reduced pressure. Compound **11** (290 mg, 0.774 mmol, 99%) was obtained as a white solid. Mp : 106-108 °C. ^{1}H NMR (400 MHz, CD_3OD) δ: 7.48 – 7.26 (m, 5H, H_a and H_b and H_c), 5.12 (s, 2H, H_d), 2.56 (t, J = 7.0 Hz, 2H, H_e), 2.44 – 2.40 (m, 2H, H_g), 2.00 – 1.91 (m, 2H, H_f), 1.90 (s, 9H, H_h). ^{13}C NMR (101 MHz, CD_3OD) δ: 172.2, 136.1, 128.2, 127.9, 127.9, 66.1, 33.7, 23.4, 17.8, 5.8. ESI-MS calcd for $C_{14}H_{22}O_2As$ $[M]^+$ 297.0836, found 297.0822.

Synthesis of (4-(benzyloxy)-4-oxobutyl) trimethylarsonium bromide (3)

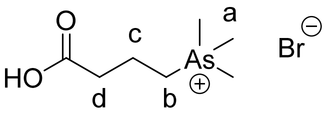

A solution of arsonium bromide **11** (100 mg, 0.265 mmol, 1 equivalent) in MeOH (20 mL) was treated with Pd/C (50 mg, 10% Pd/C) and stirred for 72 hours under $H_{2(g)}$ atmosphere. Subsequently the mixture was filtered through a pad of Celite and the solvent was removed under reduced pressure. The crude solid was washed with CH_2Cl_2 (3 × 10 mL) to afford compound **3** (73 mg, 0.252 mmol, 95%) as a white solid. Mp : 153-155 °C. ^{1}H NMR (400 MHz, D_2O) δ: 2.36 (t, J = 7.0 Hz, 2H, H_d), 2.28 – 2.19 (m, 2H, H_b), 1.82 – 1.74 (m, 2H, H_c), 1.72 (s, 9H, H_a). ^{13}C NMR (101 MHz, D_2O) δ: 177.5, 34.4, 23.1, 17.7, 6.1. ESI-MS calcd for $C_7H_{16}O_2As$ $[M]^+$ 207.0339, found 207.0366.

Synthesis of (*R*)-ethyl 4-iodo-3-hydroxybutanoate (12)[2]

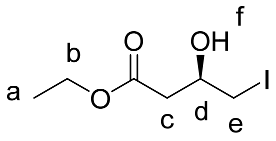

A microwave vial was charged with NaI (1.78 g, 12 mmol, 4 equivalents) and (*R*)-ethyl 4-chloro-3-hydroxybutanoate (480 µL, 3 mmol, 1 equivalent) and then suspended in acetone (5 mL) under $Ar_{(g)}$ atmosphere. The vial was capped and heated to 55 °C. After stirring for 72 hours the solution was allowed to cool to r.t. and filtered. Solvent was evaporated and the resulting crude oil was purified by flash column chromatography (SiO_2, Et_2O in *n*-heptane 50%), affording the iodo compound **12** (550 mg, 2.13 mmol, 71%) as a pale yellow oil. $[\alpha]^{25}_{D}$ +10.4 (*c* 3.03, EtOH). ^{1}H NMR (400 MHz, $CDCl_3$) δ: 4.19 (q, *J* = 7.0 Hz, 2H, H_b), 4.04 – 3.96 (m, 1H, H_d), 3.39 – 3.26 (dq, *J* = 5.5, 10.5 Hz, 2H, H_c), 2.64 (dq, *J* = 16.5, 6.0 Hz, 2H, H_e), 1.29 (t, *J* = 8.0, 6.5 Hz, 3H, H_a). ^{13}C NMR (101 MHz, $CDCl_3$) δ: 171.7, 67.5, 61.0, 40.7, 14.1, 12.0. These data are in good agreement with previously reported data.[2]

Synthesis of ethyl (*R*)-3-((tert-butyldimethylsilyl)oxy)-4-iodobutanoate (13)[2]

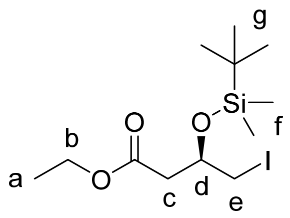

A round bottom flask was charged with NaI (638 mg, 4.26 mmol, 2 equivalents), imidazole (288 mg, 4.26 mmol, 2 equivalents) and iodide **12** (550 mg, 2.13 mmol, 1 equivalent) and then dissolved in dry DMF (8 mL) under $Ar_{(g)}$ atmosphere. Subsequently *tert*-butyl dimethyl silylchloride (3.2 mL, 3.20 mmol, 1.5 equivalents, 1 M solution in THF) was added dropwise and the resulting solution was left stirring for 16 hours. The reaction mixture was diluted with H_2O (30 mL) and Et_2O (30 mL). The aqueous layer was extracted with Et_2O (3 × 30 mL) and the combined organic extracts were washed once with brine (50 mL). The organic layers were dried over Na_2SO_4, filtered and evaporated. The resulting crude oil was purified by column chromatography (SiO_2, Et_2O in *n*-heptane 1-10%), yielding TBS protected alcohol **13** (609 mg, 1.63 mmol, 77%) as a clear colorless oil. $[\alpha]^{22}_{D}$ +30.1 (*c* 1.99, EtOH). ^{1}H NMR (400 MHz, $CDCl_3$) δ: 4.21 – 4.07 (m, 2H, H_b), 4.06 – 3.97 (m, 1H, H_d), 3.32 – 3.21 (m, 2H, H_e), 2.71 – 2.49 (m, 2H, H_c), 1.30 – 1.23 (t, *J* = 7.0 Hz, 3H, H_a), 0.86 (s, 9H, H_g), 0.11 (s, 3H, H_f), 0.06 (s, 3H, H_f). ^{13}C NMR (101 MHz, $CDCl_3$) δ: 171.1, 68.6, 60.8, 42.7, 25.8, 18.1, 14.3, 13.2, -4.4, -4.8. These data are in good agreement with previously reported data.[2]

Synthesis of (*R*)-(2-((tert-butyldimethylsilyl)oxy)-4-ethoxy-4-oxobutyl)trimethylphosphonium iodide (14)

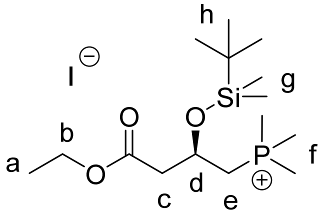

A microwave vial was charged with iodide **13** (100 mg, 0.269 mmol, 1 equivalent) and PMe_3 (1.08 mL, 1.08 mmol, 4 equivalents, 1 M in toluene) under $Ar_{(g)}$ atmosphere and the resulting solution was heated to 75 °C. After 24 hours the solution was allowed to cool down to r.t. and the solvent was removed under reduced pressure. The resulting crude oil was purified by preparative HPLC (t_r 20.1 minutes), affording phosphonium salt **14** (23 mg, 0.051 mmol, 19%) as a clear colorless oil. $[\alpha]^{25}_{D}$ -22.3 (*c* 1.01, $CHCl_3$). ^{1}H

NMR (400 MHz, CD_3OD) δ: 4.49 – 4.37 (m, 1H, H_d), 4.02 – 3.92 (m, 2H, H_b), 2.61 – 2.39 (m, 4H, H_c and H_e), 1.73 (d, J = 14.5Hz, 9H, H_f), 1.11 – 1.03 (m, 3H, H_a), 0.73 (s, 9H, H_h), 0.02 (s, 3H, H_g), 0.00 (s, 3H, H_g). ^{13}C NMR (101 MHz, CD_3OD) δ: 171.5, 66.5 (d, $J_{C\text{-}P}$ = 6.0 Hz) , 62.0, 43.9 (d, $J_{C\text{-}P}$ = 9.5 Hz), 32.3 (d, $J_{C\text{-}P}$ = 53.5 Hz), 26.3, 18.8, 14.4, 9.5 (d $J_{C\text{-}P}$ = 55.0 Hz), -3.9, -4.3. ^{31}P NMR (162 MHz, CD_3OD) δ: 24.66 (m, 1P). ESI-MS calcd for $C_{15}H_{34}O_3PSi$ $[M]^+$321.2015, found 321.2025.

Synthesis of (*R*)-(4-ethoxy-2-hydroxy-4-oxobutyl)trimethylphosphonium iodide (15)

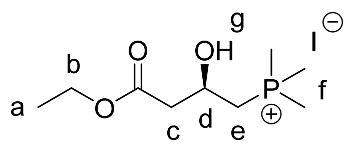

A round bottom flask was charged with phosphonium salt **14** (23 mg, 0.051 mmol, 1 equivalent) and tetrabutylammonium fluoride (TBAF, 100 µL, 0.100 mmol, 1.2 equivalents, 1 M solution in THF) and the resulting solution was stirred for 1 hour at r.t. Subsequently the solvent was removed and the crude oil was purified by preparative HPLC (t_r 7.3 minutes), affording alcohol **15** (15 mg, 0.044 mmol, 88%) as a clear colorless oil. $[\alpha]^{25}_D$ -24.4 (*c* 1.00, MeOH). ^1H NMR (400 MHz, CD_3OD) δ: 4.45 – 4.29 (m, 1H, H_d), 4.19 – 4.05 (m, 2H, H_b), 2.60 (dd, J = 6.0, 1.5 Hz, 2H, H_c), 2.51 – 2.42 (m, 2H, H_e), 1.88 (d, J = 15.0 Hz, 9H, H_f), 1.29 – 1.17 (t, J = 7.0 Hz, 3H, H_a). ^{13}C NMR (101 MHz, CD_3OD) δ: 172.1, 64.3 (d, $J_{C\text{-}P}$ = 6.5 Hz), 61.8, 44.2 (d, $J_{C\text{-}P}$ = 14.0 Hz), 31.6 (d, $J_{C\text{-}P}$ = 56.0 Hz), 14.5, 9.1 (d, $J_{C\text{-}P}$ = 55.5 Hz). ^{31}P NMR (162 MHz, CD_3OD) δ: 26.49 (m, 1P). ESI-MS calcd for $C_9H_{20}O_3P$ $[M]^+$ 207.1150, found 207.1157.

Synthesis of (*R*)-(3-carboxy-2-hydroxypropyl)trimethylphosphonium trifluoroacetate (5)

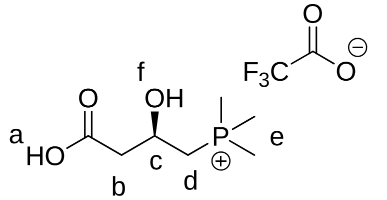

To an aqueous solution of NaOH (4.4 mg, 0.110 mmol, 2.5 equivalents in H_2O) and 1,4-dioxane (5 mL) was added ester **15** (15 mg, 0.044 mmol, 1 equivalent). The solution was stirred for 2 hours at r.t. before acidifying to pH 1 with HCl (3 N, 1 M in H_2O). The solvent was evaporated (caution, do not heat for an extend period of time, as this induced product degradation) and the crude solid was suspended in a minimal amount of MeOH, filtered and purified by preparative HPLC (t_r 6.5 minutes, mobile phase contain 0.1% TFA), affording acid **5** (11 mg, 0.0375 mmol, 85%) as a clear colorless oil. $[\alpha]^{25}_D$ -11.0 (*c* 1.51, MeOH). ^1H NMR (400 MHz, CD_3OD) δ: 4.43 – 4.31 (m, 1H, H_c), 2.62 – 2.58 (dd, J = 7.0, 1.5 Hz, 2H, H_b), 2.51 (m, 2H, H_d), 1.91 (d, J = 14.5 Hz, 9H, H_e). ^{13}C NMR (101 MHz, CD_3OD) δ: 177.0, 65.0 (d, $J_{C\text{-}P}$ = 6.5 Hz), 45.6 (d, $J_{C\text{-}P}$ = 14.0 Hz), 31.7 (d, $J_{C\text{-}P}$ = 55.5 Hz), 9.2 (d, $J_{C\text{-}P}$ = 55.5 Hz). ^{31}P NMR (162 MHz, CD_3OD) δ: 26.04 (m, 1P). ESI-MS calcd for $C_7H_{16}O_3P$ $[M]^+$ 179.0837, found 179.0841.

Synthesis of (*R*)-(2-((tert-butyldimethylsilyl)oxy)-4-ethoxy-4-oxobutyl)trimethylarsonium iodide (16)

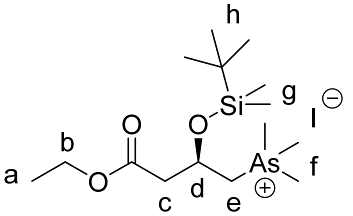

A microwave vial was charged with iodide **13** (100 mg, 0.269 mmol, 1 equivalent) and dissolved in dry MeCN (1 mL) and under $Ar_{(g)}$ atmosphere. To this was added $AsMe_3$ (115 µL, 1.08 mmol, 4 equivalents) and the resulting solution was heated to 75 °C. After 24 hours the solution was allowed to cool down to r.t. and the solvent was removed under reduced pressure. The resulting crude oil was purified by preparative HPLC (t_r 20.3 minutes), affording arsonium salt **16** (32 mg, 0.065 mmol, 24%) as a clear colorless oil. $[\alpha]^{25}_{D}$ -20.3 (*c* 0.97, $CHCl_3$). ^{1}H NMR (400 MHz, CD_3OD) δ: 4.49 (quint, *J* = 5.5 Hz, 1H, H_d), 3.97 (q, *J* = 7.0 Hz, 2H, H_b), 2.65 (d, *J* = 5.5 Hz, 2H, H_c), 2.59 – 2.45 (m, 2H, H_e), 1.77 (s, 9H, H_f), 1.09 (t, *J* = 7.0 Hz, 3H, H_a), 0.75 (s, 9H, H_h), 0.01 (2 × s, *J* = 4.5 Hz, 2 × 3H, H_g). ^{13}C NMR (101 MHz, CD_3OD) δ: 171.6, 67.3, 62.1, 43.8, 34.6, 26.4, 18.9, 14.4, 9.3, -3.9, -4.6. ESI-MS calcd for $C_{15}H_{34}O_3AsSi$ $[M]^+$ 365.1493, found 365.1506.

Synthesis of (*R*)-(4-ethoxy-2-hydroxy-4-oxobutyl)trimethylarsonium iodide (17)

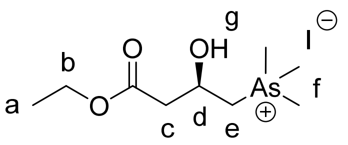

A round bottom flask was charged with arsonium salt **16** (32 mg, 0.065 mmol, 1 equivalent) and tetrabutylammonium fluoride (TBAF, 100 µL, 0.100 mmol, 1.2 equivalents, 1 M solution in THF) and the resulting solution was stirred for 1 hour at r.t. Subsequently the solvent was removed and the crude oil was purified by preparative HPLC (t_r 7.5 minutes), affording alcohol **17** (23 mg, 0.061 mmol, 94%) as a clear colorless oil. $[\alpha]^{25}_{D}$ -24.9 (*c* 1.02, MeOH). ^{1}H NMR (400 MHz, CD_3OD) δ: 4.40 – 4.31 (m, 1H, H_d), 4.14 (qd, *J* = 7.0, 1.0 Hz, 2H, H_b), 2.69 – 2.57 (m, 4H, H_c and H_e), 1.89 (s, 9H, H_f), 1.24 (t, *J* = 7.0 Hz, 3H, H_a). ^{13}C NMR (101 MHz, CD_3OD) δ: 172.1, 64.6, 61.8, 44.0, 33.8, 14.5, 8.5. ESI-MS calcd for $C_9H_{20}O_3As$ $[M]^+$ 251.0628, found 251.0635.

Synthesis of (*R*)-(3-carboxy-2-hydroxypropyl)trimethylarsonium iodide (6)

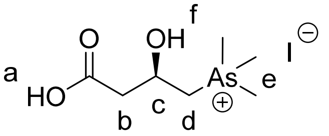

To an aqueous solution of NaOH (5 mL, 4 M in H_2O) and 1,4-dioxane (5 mL) was added ester **17** (23 mg, 0.061 mmol, 1 equivalent). The solution was stirred for 2 hours before acidifying to pH 3 with HCl (1 M in H_2O). The solvent was evaporated (caution, do not heat for an extend period of time, as this induced product degradation) and the crude solid was suspended in a minimal amount of MeOH, filtered and purified by preparative HPLC (t_r 6.6 minutes), affording acid **6** (15 mg, 0.043 mmol, 71%) as a clear colorless oil. $[\alpha]^{25}_{D}$ -12.9 (*c* 1.04, MeOH). ^{1}H NMR (400 MHz, CD_3OD) δ: 4.32 – 4.24 (m, 1H, H_c), 2.61 (qd, *J* = 13.5, 7.5 Hz, 2H, H_b), 2.48 (d, *J* = 6.5 Hz, 2H, H_d), 1.89 (s, 9H, H_e). ^{13}C NMR (101 MHz, CD_3OD) δ: 176.8, 65.3, 45.2, 34.0, 8.5. ESI-MS calcd for $C_7H_{16}O_3As$ $[M]^+$ 223.0315, found 223.0325.

Synthesis of (*S*)-ethyl 4-iodo-3-hydroxybutanoate (18)[2]

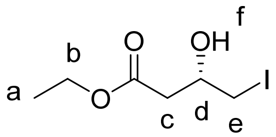

A microwave vial was charged with NaI (1.77 g, 12 mmol, 4 equivalents) and ethyl (*S*)-4-chloro-3-hydroxybutanoate (480 µL, 3 mmol, 1 equivalent) and then suspended in acetone (5 mL) under $Ar_{(g)}$ atmosphere. The vial was capped and heated to 55 °C. After stirring for 72 hours the solution was allowed to cool down to r.t. and filtered. Solvent was evaporated and the resulting crude oil was purified by flash column chromatography (SiO_2, Et_2O in *n*-heptane 50%), affording iodo compound **18** (537 mg, 2.08 mmol, 69%) as a pale yellow oil. $[\alpha]^{25}_D$ -10.1 (*c* 2.99, EtOH). ^1H NMR (400 MHz, $CDCl_3$) δ: 4.19 (q, *J* = 7.0 Hz, 2H, H_b), 4.06 – 3.94 (m, 1H, H_d), 3.40 – 3.25 (m, 2H, H_c), 2.65 (dq, *J* = 16.5, 6.0), 1.29 (t, *J* = 7.0 Hz, 3H, H_a). ^{13}C NMR (101 MHz, $CDCl_3$) δ: 171.9, 67.6, 61.2, 40.8, 14.3, 12.2. These data are in good agreement with previously reported data.[2]

Synthesis of ethyl (*S*)-3-((tert-butyldimethylsilyl)oxy)-4-iodobutanoate (19)[2]

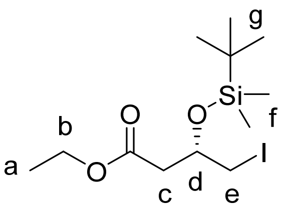

A round bottom flask was charged with NaI (620 mg, 4.14 mmol, 2 equivalents), imidazole (280 mg, 4.14 mmol, 2 equivalents) and iodide **18** (537 mg, 2.07 mmol, 1 equivalent) and then dissolved in dry DMF (8 mL) under $Ar_{(g)}$ atmosphere. Subsequently *tert*-butyl dimethyl silylchloride (3.1 mL, 3.10 mmol, 1.5 equivalents, 1 M solution in THF) was added dropwise and the resulting solution was left stirring for 16 hours. The reaction mixture was diluted with H_2O (30 mL) and Et_2O (30 mL). The aqueous layer was extracted with Et_2O (3 × 30 mL) and the combined organic extracts were washed once with brine (50 mL). The organic layers were dried over Na_2SO_4, filtered and evaporated. The resulting crude oil was purified by column chromatography (SiO_2, Et_2O in *n*-heptane 1-10%), yielding TBS protected alcohol **19** (485 mg, 1.64 mmol, 79%) as a clear colorless oil. $[\alpha]^{22}_D$ -29.1 (*c* 2.10, EtOH). ^1H NMR (400 MHz, $CDCl_3$) δ 4.13 – 4.00 (m, 2H, H_b), 4.00 – 3.92 (m, 1H, H_d), 3.25 – 3.18 (m, 2H, H_e) 2.66 – 2.41 (m, 2H, H_c), 1.20 (t, *J* = 7.0 Hz, 3H, H_a), 0.82 (s, *J* = 2.5 Hz, 9H, H_g), 0.05 (s, 3H, H_f), 0.00 (s, 3H, H_f). ^{13}C NMR (101 MHz, $CDCl_3$) δ: 171.1, 68.5, 60.7, 42.7, 25.8, 18.1, 14.3, 13.1, -4.4, -4.8. These data are in good agreement with previously reported data.[2]

Synthesis of (*S*)-(2-((tert-butyldimethylsilyl)oxy)-4-ethoxy-4-oxobutyl)trimethylphosphonium iodide (20)

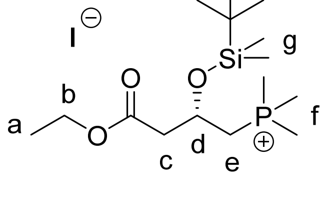

A microwave vial was charged with iodide **19** (100 mg, 0.269 mmol, 1 equivalent) and PMe_3 (1.08 mL, 1.08 mmol, 4 equivalents, 1 M in toluene) under $Ar_{(g)}$ atmosphere and the resulting solution was heated to 75 °C. After 24 hours the solution was allowed to cool down to r.t. and the solvent was removed under reduced pressure. The resulting crude oil was purified by preparative HPLC (t_r 20.1 minutes), affording phosphonium salt **20** (25 mg, 0.056 mmol, 20%) as a clear colorless oil. $[\alpha]^{25}_D$ +22.0 (*c* 1.03, $CHCl_3$). ^1H NMR (400 MHz,

CD_3OD) δ: 4.50 – 4.35 (m, 1H, H_d), 3.97 (qd, *J* = 7.0, 1.0 Hz, 2H, H_b), 2.63 – 2.40 (m, 4H, H_c and H_e), 1.74 (d, *J* = 14.5 Hz, 9H, H_f), 1.08 (t, *J* = 7.0 Hz, 3H, H_a), 0.74 (s, *J* = 3.0 Hz, 9H, H_h), 0.01 (s, 3H, H_g), 0.00 (s, 3H, H_g). ^{13}C NMR (101 MHz, CD_3OD) δ: 171.5, 66.5 (d, J_{C-P} = 6.0 Hz), 62.0, 43.9 (d, J_{C-P} = 9.5 Hz), 32.3 (d, J_{C-P} = 54.0 Hz), 26.4, 18.8, 14.4, 9.5 (d, J_{C-P} = 55.0 Hz), -3.9, -4.3. ^{31}P NMR (162 MHz, CD_3OD) δ: 24.67 (m, 1P). ESI-MS calcd for $C_{15}H_{34}O_3PSi$ $[M]^+$ 321.2015, found 321.2024.

Synthesis of (*S*)-(4-ethoxy-2-hydroxy-4-oxobutyl)trimethylphosphonium iodide (21)

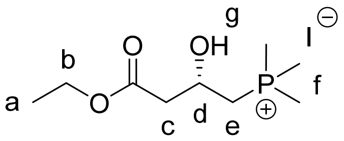

A round bottom flask was charged with phosphonium salt **20** (25 mg, 0.056 mmol, 1 equivalent) and tetrabutylammonium fluoride (TBAF, 100 µL, 0.100 mmol, 1.2 equivalents, 1 M solution in THF) and the resulting solution was stirred for 1 hour. Subsequently the solvent was removed and the crude oil was purified by preparative HPLC (t_r 7.3 minutes), affording alcohol **21** (16 mg, 0.048 mmol, 84%) as a clear colorless oil. $[\alpha]^{25}_D$ +23.9 (*c* 0.98, MeOH). ^{1}H NMR (400 MHz, CD_3OD) δ: 4.44 – 4.32 (m, 1H, H_d), 4.14 (qd, *J* = 7.0, 1.0 Hz, 2H, H_b), 2.60 (dd, *J* = 6.0, 1.5 Hz, 2H, H_c), 2.50 – 2.43 (m, 2H, H_e), 1.88 (d, *J* = 15.0 Hz, 9H, H_f), 1.24 (t, *J* = 7.0 Hz, 3H, H_a). ^{13}C NMR (101 MHz, CD_3OD) δ: 172.1 (d, J_{C-P} = 2.5 Hz), 64.3 (d, J_{C-P} = 6.0 Hz), 61.9, 44.2 (d, J_{C-P} = 14.0 Hz), 31.6 (d, J_{C-P} = 56.0 Hz), 14.5, 9.1 (d, J_{C-P} = 55.5 Hz). ^{31}P NMR (162 MHz, CD_3OD) δ: 26.05 (m, 1P). ESI-MS calcd for $C_9H_{20}O_3P$ $[M]^+$ 207.1150, found 207.1161.

Synthesis of (*S*)-(3-carboxy-2-hydroxypropyl)trimethylphosphonium trifluoroacetate (7)

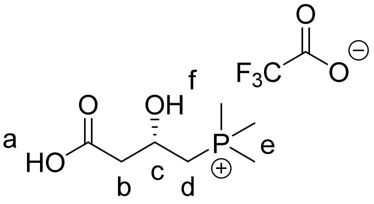

To an aqueous solution of NaOH (4.8 mg, 0.120 mmol, 2.5 equivalents in H_2O) and 1,4-dioxane (5 mL) was added ester **21** (16 mg, 0.048 mmol, 1 equivalent). The solution was stirred for 2 hours before acidifying to pH 1 with HCl (1 M in H_2O). The solvent was evaporated (caution, do not heat for an extend period of time, as this induced product degradation) and the crude solid was suspended in a minimal amount of MeOH, filtered and purified by preparative HPLC (t_r 6.5 minutes, mobile phase contains 0.1% TFA), affording acid **7** (12 mg, 0.042 mmol, 87%) as a clear colorless oil. $[\alpha]^{25}_D$ +11.4 (*c* 1.52, MeOH). ^{1}H NMR (400 MHz, CD_3OD) δ: 4.30 (brs, 1H, H_c), 2.56 – 2.35 (m, 4H, H_b and H_d), 1.89 (d, *J* = 14.5 Hz, 9H, H_e). ^{13}C NMR (101 MHz, CD_3OD) δ: 176.5, 64.9 (d, J_{C-P} = 6.0 Hz), 45.3 (d, J_{C-P} = 13.9 Hz), 31.7 (d, J_{C-P} = 55.4 Hz), 9.2 (d, J_{C-P} = 55.4 Hz). ^{31}P NMR (162 MHz, CD_3OD) δ: 26.06 (m, 1P). ESI-MS calcd for $C_7H_{16}O_2P$ $[M]^+$ 179.0837, found 179.0840.

Synthesis of (*S*)-(2-((tert-butyldimethylsilyl)oxy)-4-ethoxy-4-oxobutyl)trimethylarsonium iodide (22)

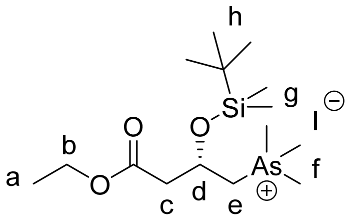

A microwave vial was charged with iodide **19** (100 mg, 0.269 mmol, 1 equivalent) and dissolved in dry MeCN (1 mL) under $Ar_{(g)}$ atmosphere. To this was added $AsMe_3$ (115 µL, 1.08 mmol, 4 equivalents) and the resulting solution was heated to 75 °C. After 24 hours the solution was allowed to cool down to r.t. and the solvent was removed under reduced atmosphere. The resulting crude oil was purified by preparative HPLC (t_r 20.3 minutes), affording arsonium salt **22** (30 mg, 0.0609 mmol, 23%) as a clear colorless oil. $[\alpha]^{25}_{D}$ +19.9 (*c* 1.00, $CHCl_3$). ^1H NMR (400 MHz, CD_3OD) δ: 4.49 (dq, *J* = 11.0, 5.5 Hz, 1H, H_d), 3.98 (q, *J* = 7.0 Hz, 2H, H_b), 2.67 (d, *J* = 5.5 Hz, 2H, H_c), 2.55 – 2.49 (m, 2H, H_e), 1.78 (s, 9H, H_f), 1.09 (t, 3H, H_a), 0.75 (s, 9H, H_h), 0.01 (s, 3H, H_g), 0.00 (s, 3H, H_g). ^{13}C NMR (101 MHz, CD_3OD) δ: 171.6, 67.3, 62.1, 43.9, 34.6, 26.4, 18.9, 14.4, 9.3, -3.9, -4.6. ESI-MS calcd for $C_{15}H_{34}O_3AsSi$ $[M]^+$ 365.1493, found 365.1503.

Synthesis of (*S*)-(4-ethoxy-2-hydroxy-4-oxobutyl)trimethylarsonium iodide (23)

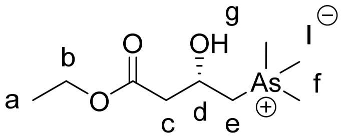

A round bottom flask was charged with arsonium salt **22** (30 mg, 0.0609 mmol, 1 equivalent) and tetrabutylammonium fluoride (TBAF, 75 µL, 0.075 mmol, 1.2 equivalents, 1 M solution in THF) and the resulting solution was stirred for 1 hour. Subsequently the solvent was removed and the crude oil was purified by preparative HPLC (t_r 7.5 minutes), affording alcohol **23** (20 mg, 0.053 mmol, 86%) as a clear colorless oil. $[\alpha]^{25}_{D}$ +23.9 (*c* 1.00, MeOH). ^1H NMR (400 MHz, CD_3OD) δ: 4.40 – 4.31 (m, 1H, H_d), 4.14 (q, *J* = 7.0, 2H, H_b), 2.66 – 2.58 (m, 4H, H_c and H_e), 1.89 (s, 9H, H_f), 1.24 (t, *J* = 7.0 Hz, 3H, H_a). ^{13}C NMR (101 MHz, CD_3OD) δ: 172.1, 64.6, 61.9, 44.0, 33.8, 14.5, 8.5. ESI-MS calcd for $C_9H_{20}O_3As$ $[M]^+$ 251.0628, found 251.0637.

Synthesis of (*S*)-(3-carboxy-2-hydroxypropyl)trimethylarsonium iodide (8)

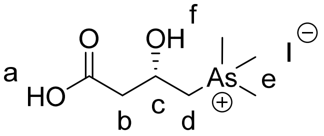

To an aqueous solution of NaOH (5 mL, 4 M in H_2O) and 1,4-dioxane (5 mL) was added ester **23** (20 mg, 0.053 mmol, 1 equivalent). The solution was stirred for 2 hours before acidifying to pH 1 with HCl (1 M in H_2O). The solvent was evaporated (caution, do not heat for an extend period of time, as this induced product degradation) and the crude solid was suspended in a minimal amount of MeOH, filtered and purified by preparative HPLC (t_r 6.6 minutes), affording acid **8** (12 mg, 0.034 mmol, 65%) as a clear colorless oil. $[\alpha]^{25}_{D}$ +13.1 (*c* 1.02, MeOH). ^1H NMR (400 MHz, CD_3OD) δ: 4.38 – 4.29 (m, 1H, H_c), 2.70 – 2.61 (m, 2H, H_d), 2.58 (d, *J* = 6.5 Hz, 2H, H_b), 1.88 (s, *J* = 7.0 Hz, 9H, H_e). ^{13}C NMR (101 MHz, CD_3OD) δ: 174.0, 64.7, 43.8, 33.9, 8.5. ESI-MS calcd for $C_7H_{16}O_3As$ $[M]^+$ 223.0315, found 223.0315.

2. Supporting figures

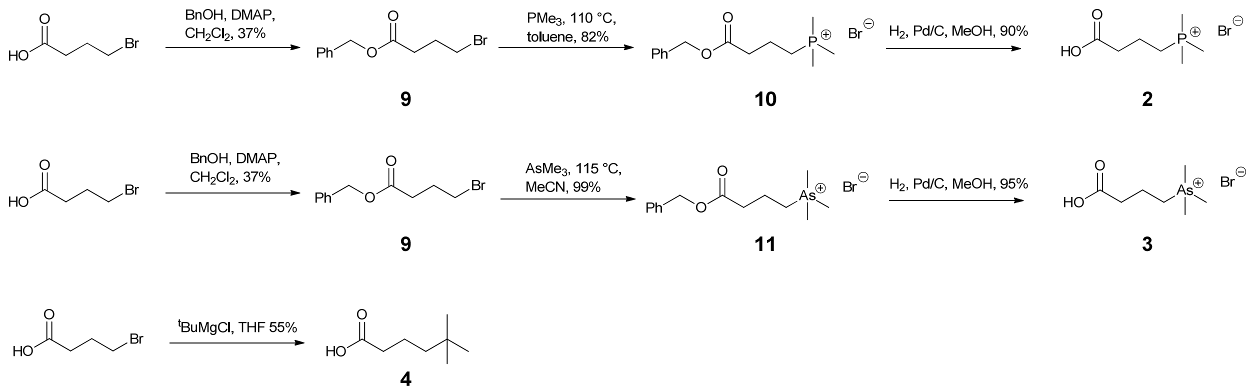

Figure S1. Syntheses of the phospha-(**2**), arsa-(**3**) and carba-(**4**) analogues of γBB.

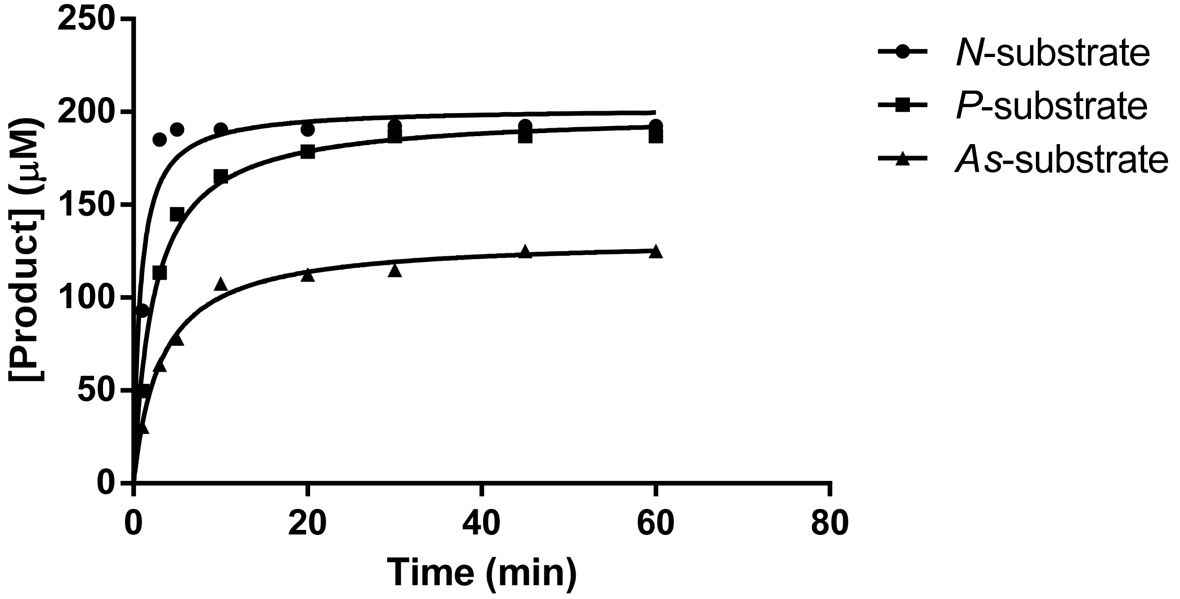

Figure S2. Time-course analyses of the psBBOX-catalysed hydroxylation of γBB (**1**, circle), phospha (**2**, square) and arsa (**3**, triangle) as observed by LC-MS. Conditions used in these experiments are: psBBOX (1 µM), 2OG (1.5 mM), ascorbate (5 mM), $FeSO_4$ (50 µM) and substrate (50 µM) in a TRIS (20 mM), NaCl (200 mM) buffer at pH 7.5.

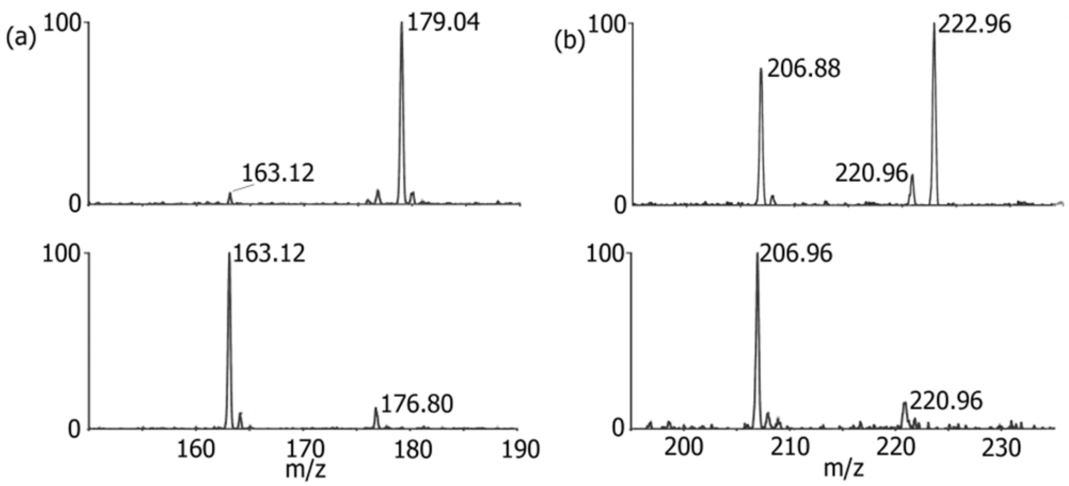

Figure S3. a) Hydroxylation of phospha-substrate **2** in the presence of psBBOX (top) and in the absence of psBBOX (bottom); b) Hydroxylation of arsa-substrate **3** in the presence (top) and in the absence of psBBOX (bottom). A typical reaction used the following conditions psBBOX (2 μM), 2OG (1.5 mM), ascorbate (5 mM), $FeSO_4$ (50 μM) and substrate (200 μM) in Tris buffer pH 7.5. Peaks at 176.80 Da ($M+H^+$) and 220.96 Da ($M+2Na^+$) derive from sodium ascorbate. Reactions were quenched with acetonitrile (80% v/v final concentration) after 1 hour incubation at 23 °C.

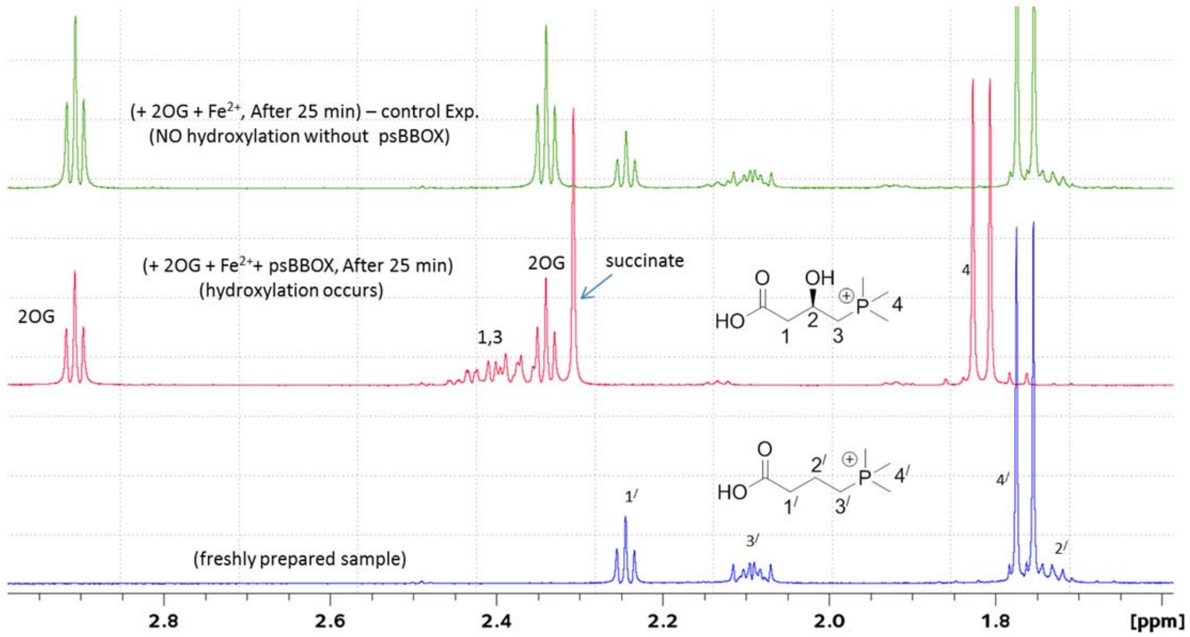

Figure S4. Hydroxylation of phospha-substrate **2** (bottom) in the presence (middle) and absence (top) of psBBOX.

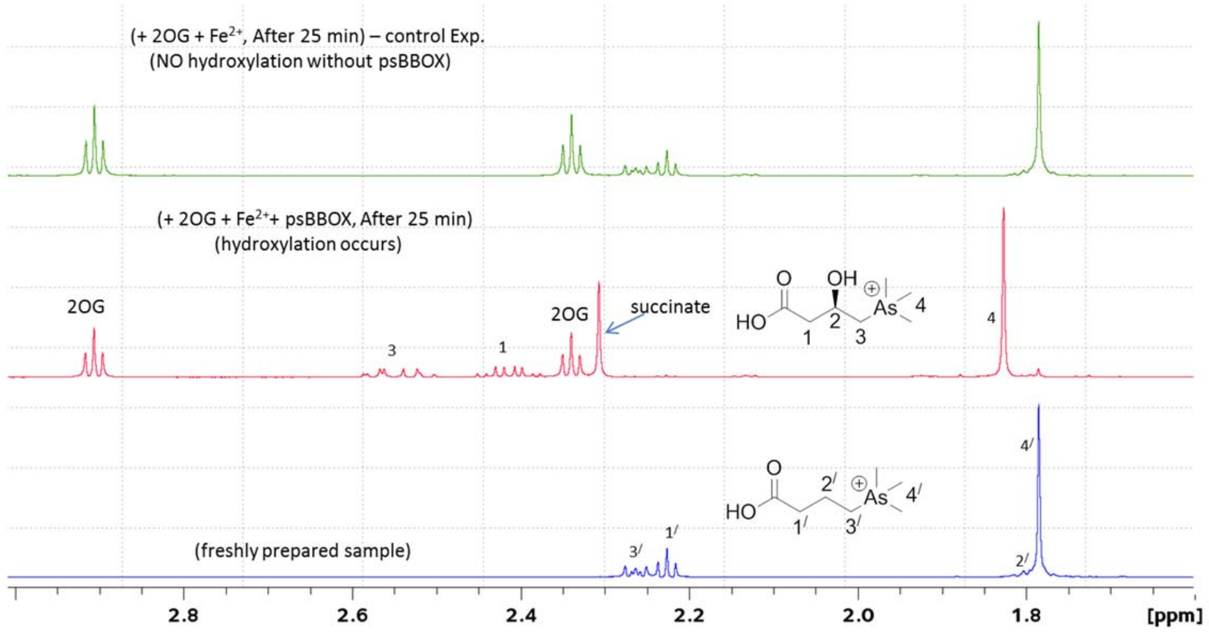

Figure S5. Hydroxylation of arsa-substrate **3** (bottom) in the presence (middle) and absence (top) of psBBOX.

^{1}H NMR (700 MHz; D_2O): δ/ppm **2**; br, m, 4.21, **1**; dd, 2.36, **3**; dd, 2.31, **4**; s, 1.74.

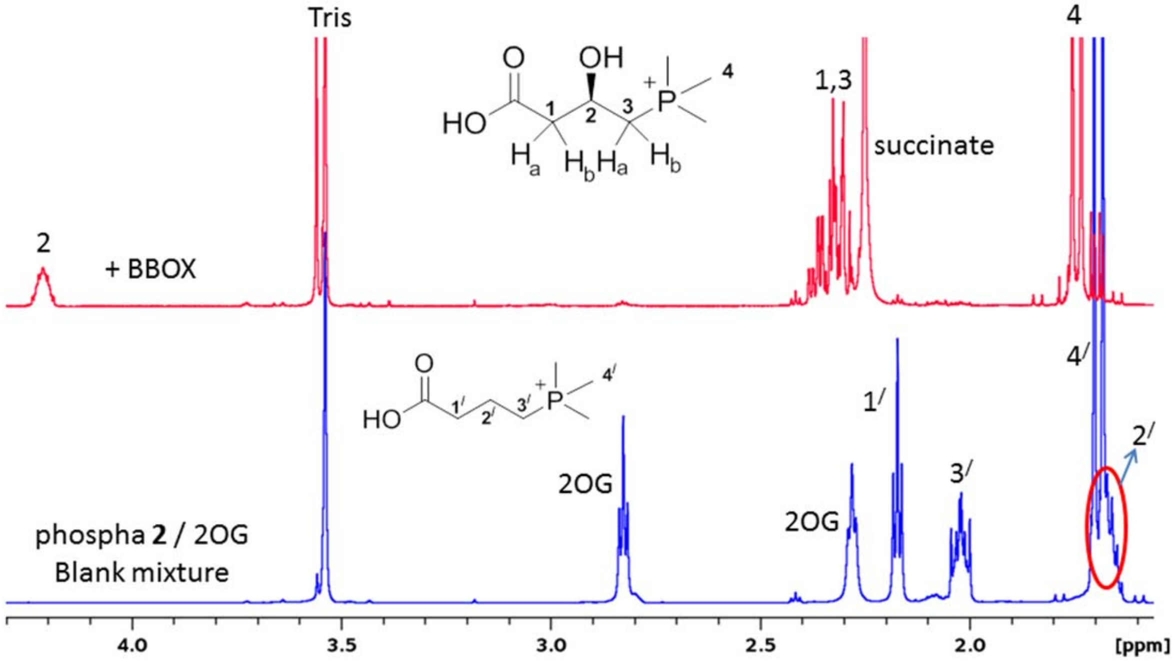

Figure S6. ^{1}H NMR assignment of the phospha-substrate **2** (bottom) and its (3*R*)-hydroxylated product **5** (top).

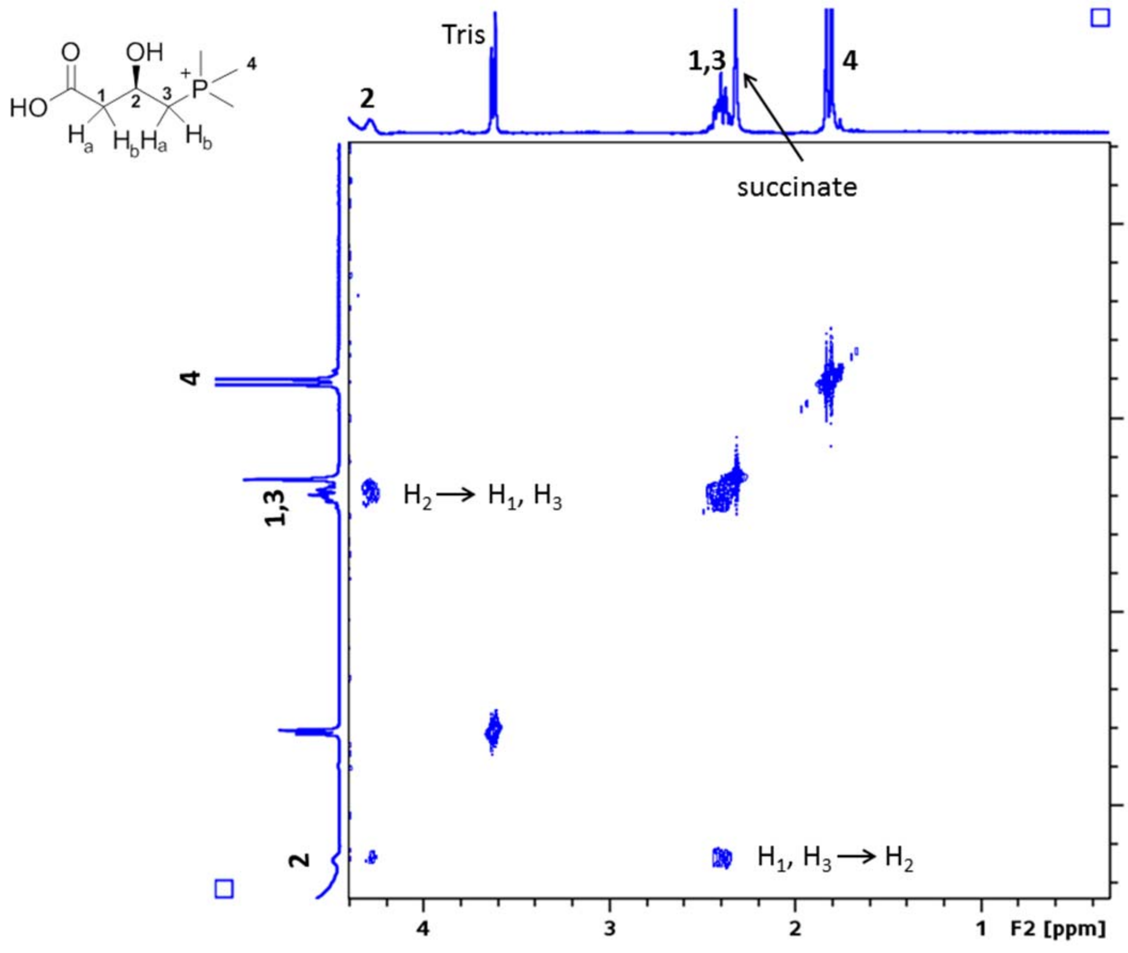

Figure S7. COSY assignment of **5** from psBBOX-catalysed hydroxylation of **2.**

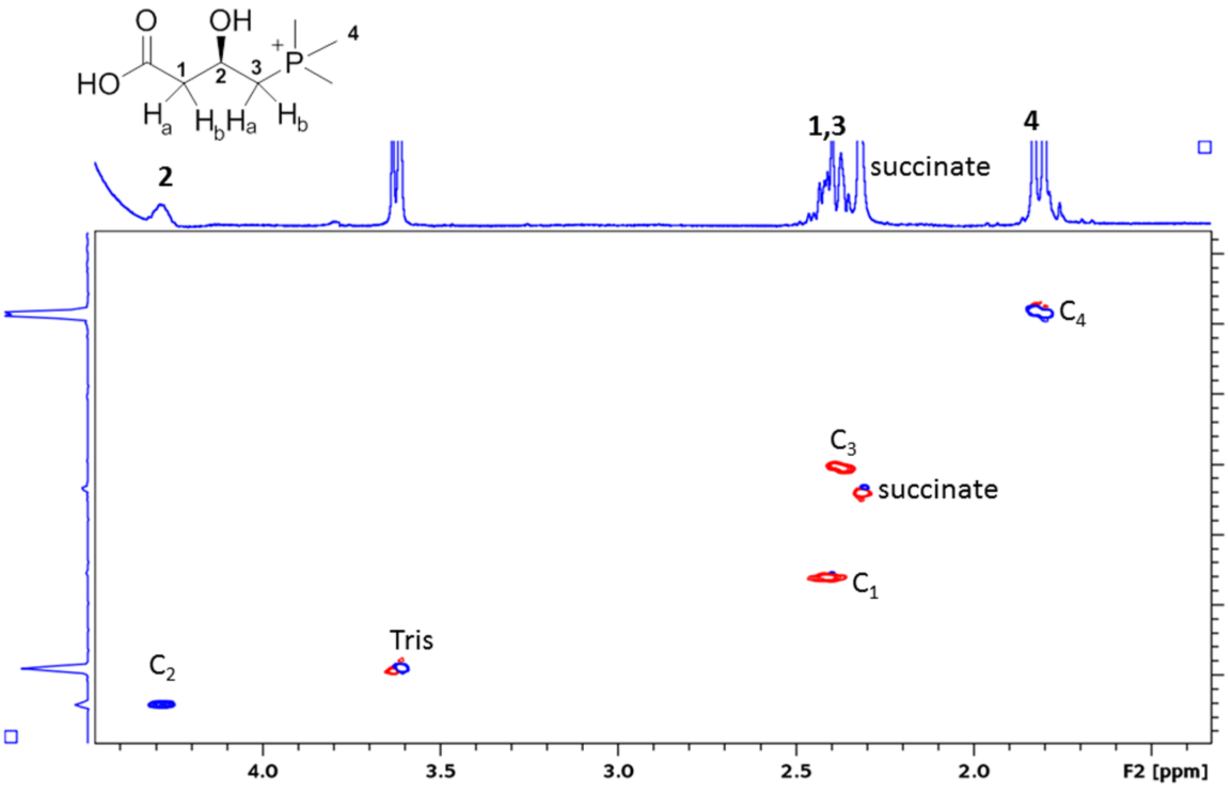

^{13}C NMR (500 MHz; D_2O): δ/ppm **2**; 64.1, **1**; 46.1, **3**; 30.4, **4**; 8.2.

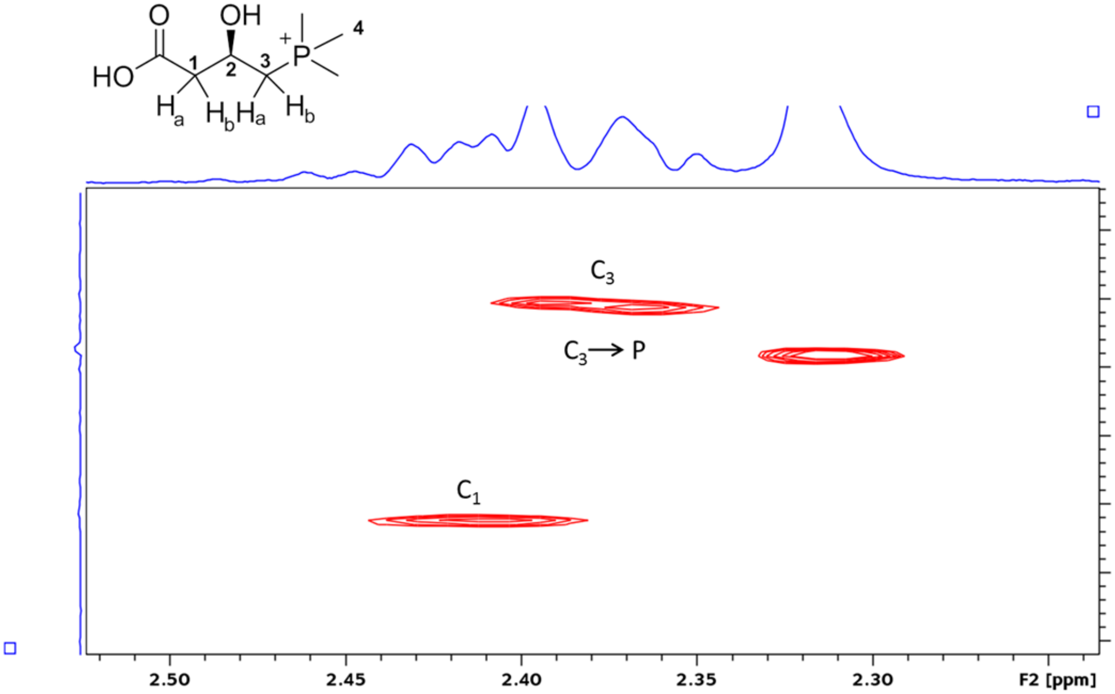

Figure S8. ^{1}H-^{13}C HSQC assignment of **5** from psBBOX-catalysed hydroxylation of **2** (top) and a zoomed view (bottom) highlighting the presence of phosphorus coupling with C3.

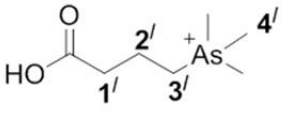

^{1}H NMR (700 MHz; D_2O): δ/ppm **2**, br, m, 4.19, **3**, dd, 2.47, **1**, dd, 2.33, **4**, s, 1.75.

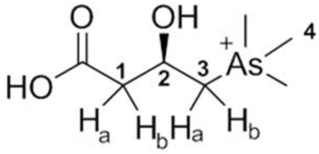

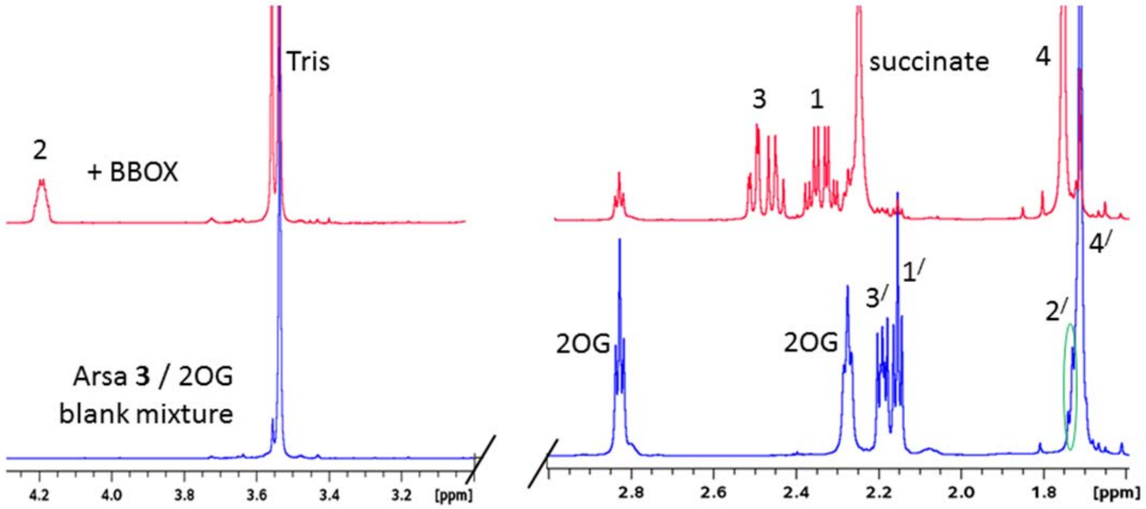

Figure S9. ^{1}H NMR assignment of arsa-substrate **3** (bottom) and its (3*R*)-hydroxylated product **6** (top).

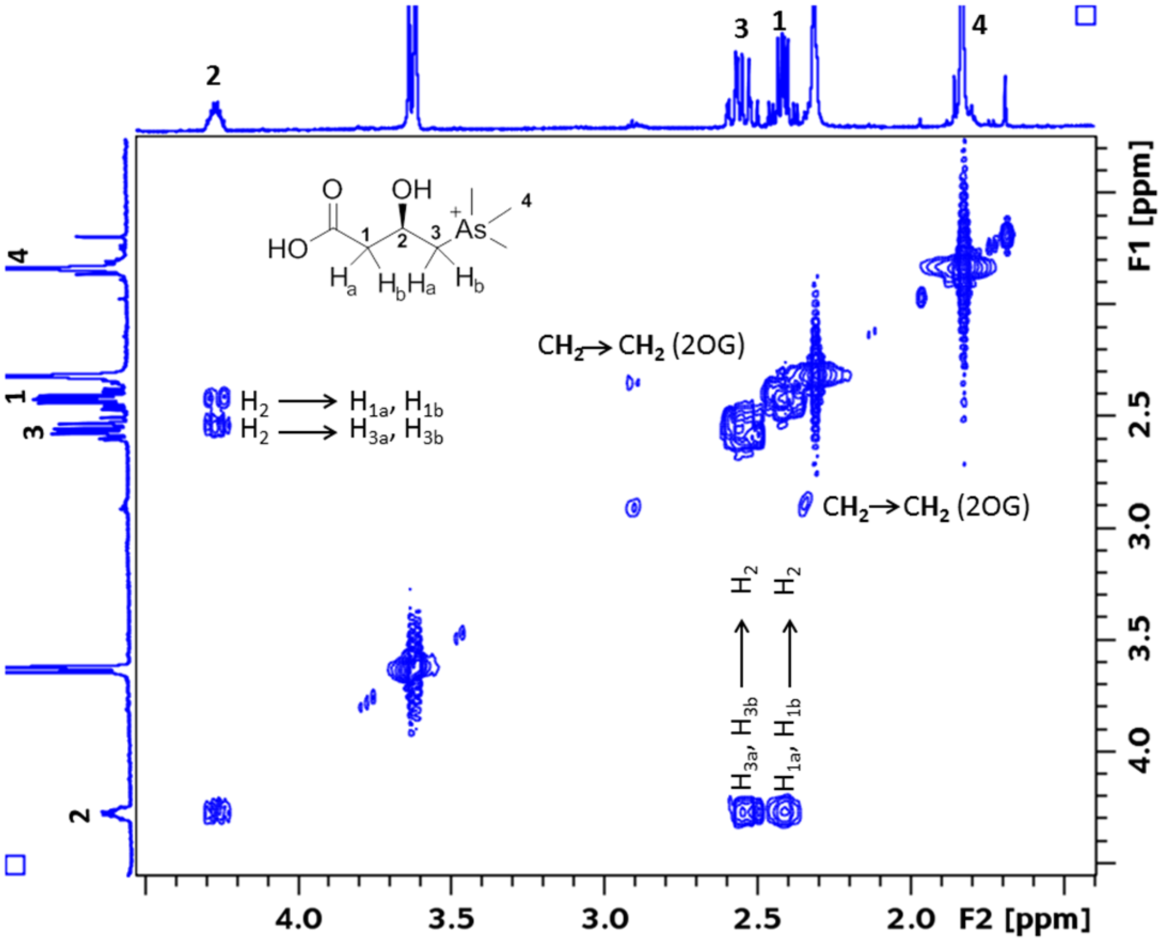

Fig. S10 COSY assignment of **6** from psBBOX-catalysed hydroxylation of **3.**

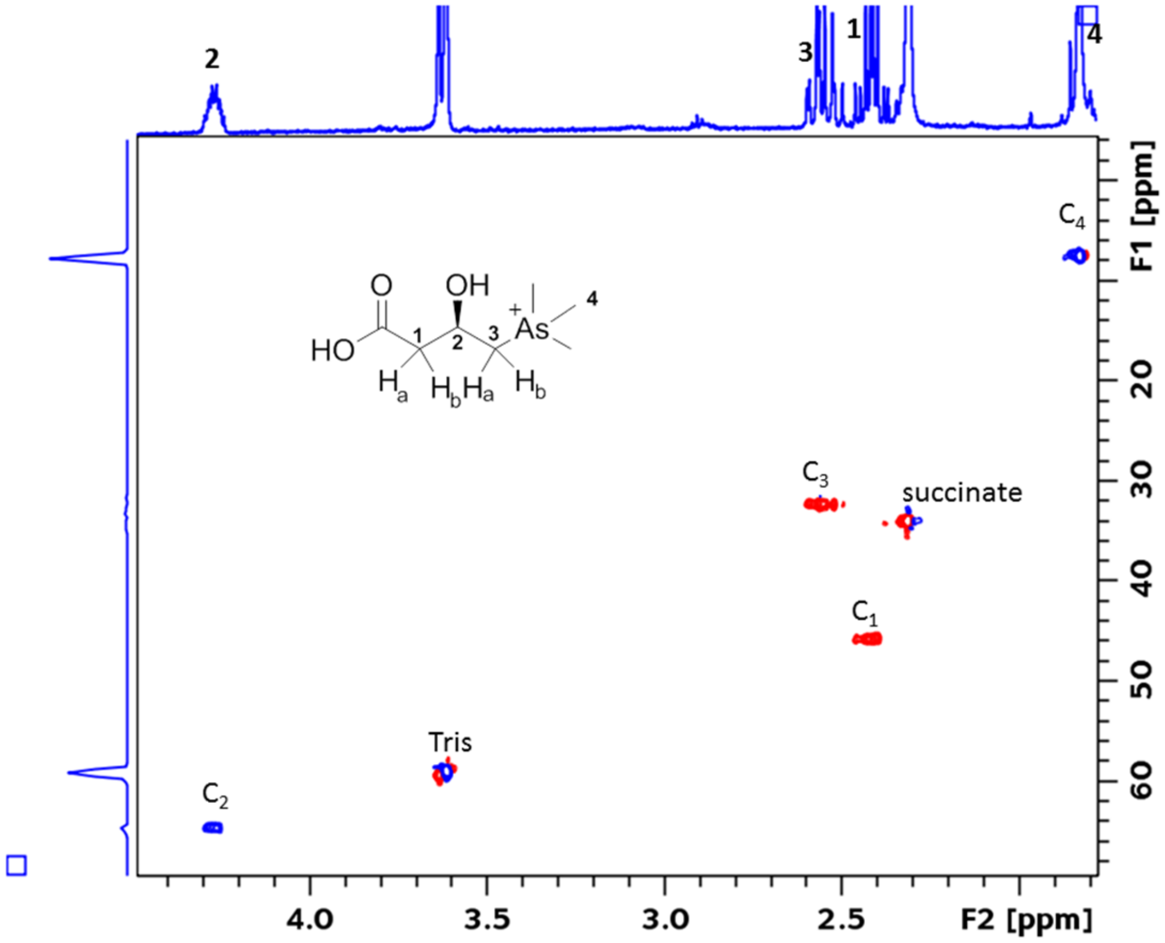

^{13}C NMR (500 MHz; D_2O): δ/ppm **2**; 64.4, **1**; 45.9, **3**; 32.6, **4**; 7.3.

Figure S11. ^{1}H-^{13}C HSQC assignment of **6** from psBBOX-catalysed hydroxylation of **3**.

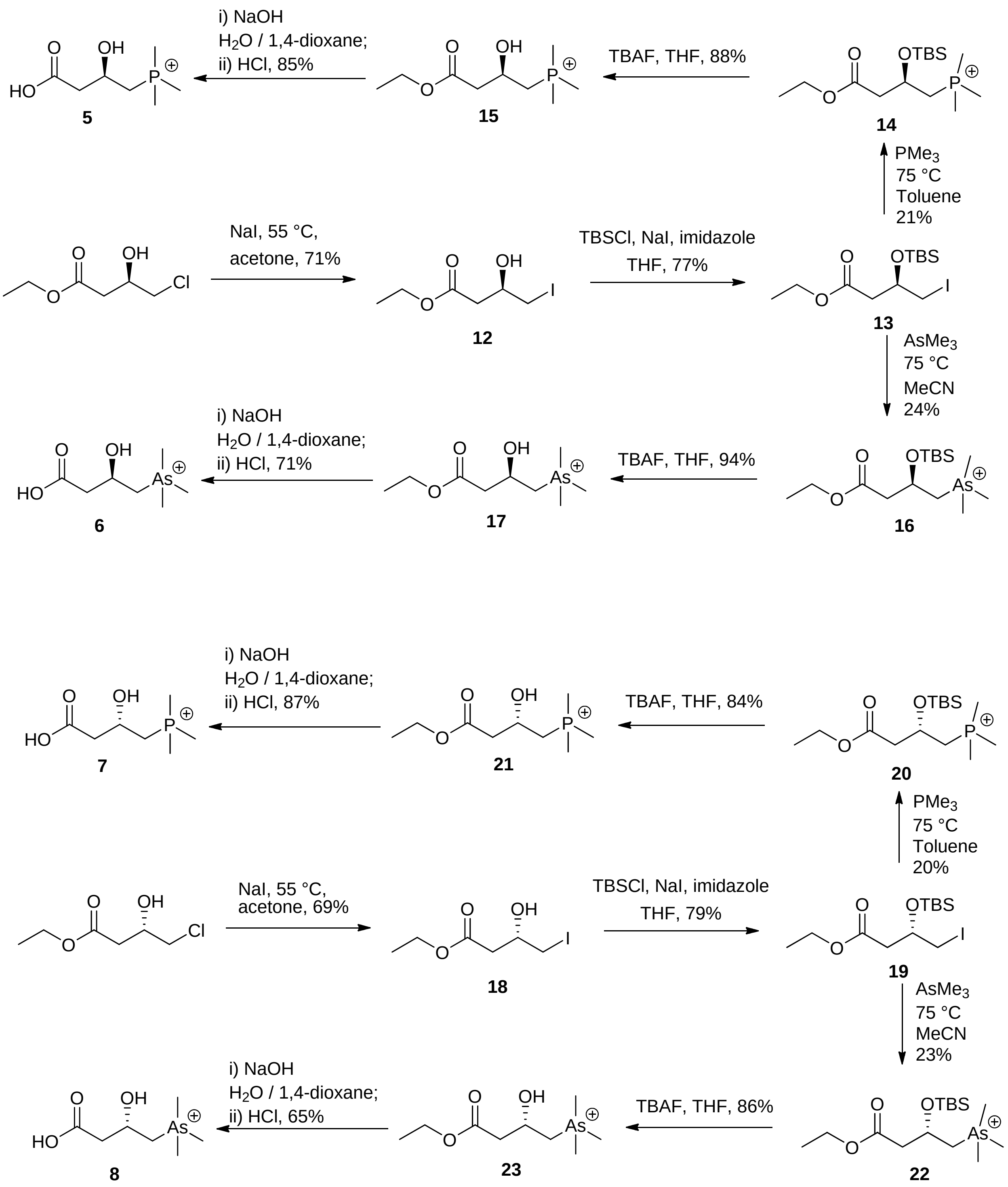

Figure S12. Syntheses of the (3*R*)- and (3*S*)-hydroxylated phospha **5**/**7** and arsa **6**/**8** compounds.

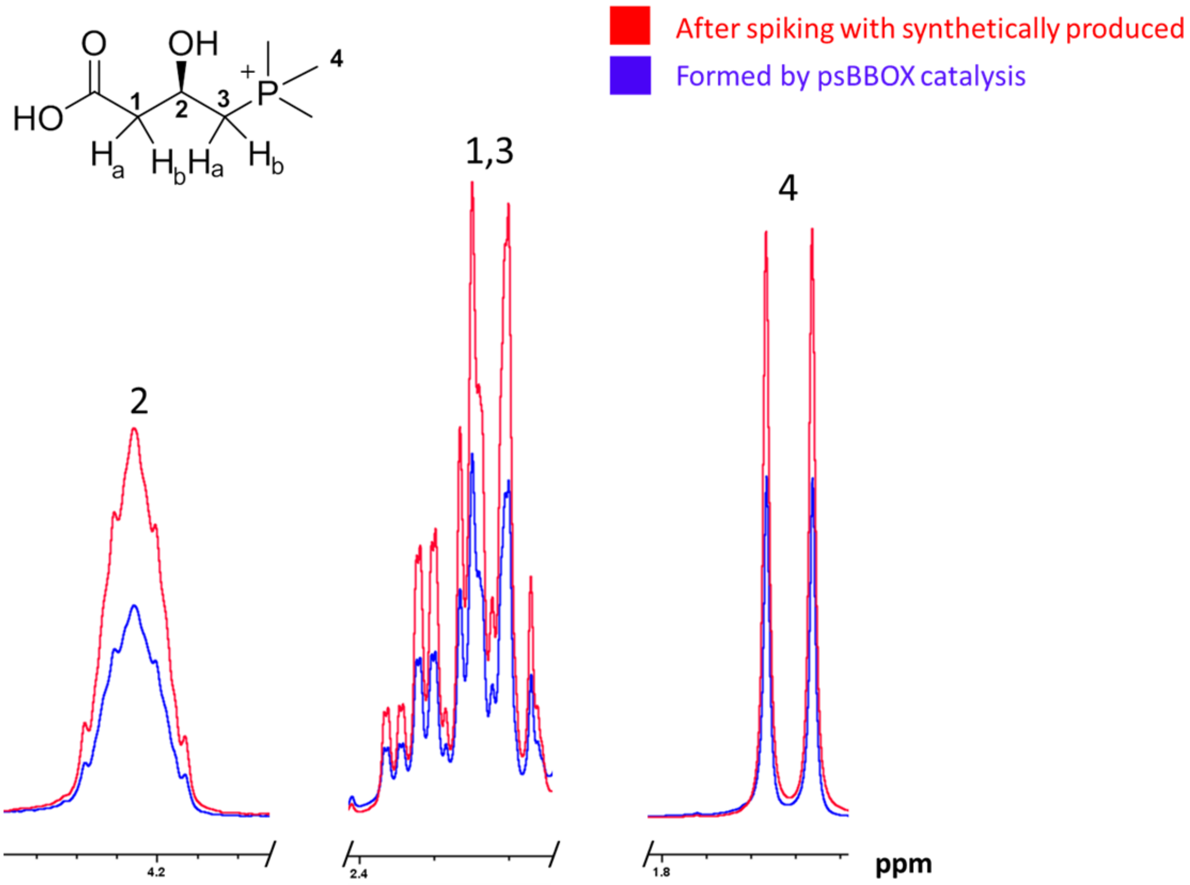

Figure S13. ^{1}H NMR assignment of hydroxylated phospha-analogue **5** before (blue) and after addition of synthetic (3*R*)-**5** (red).

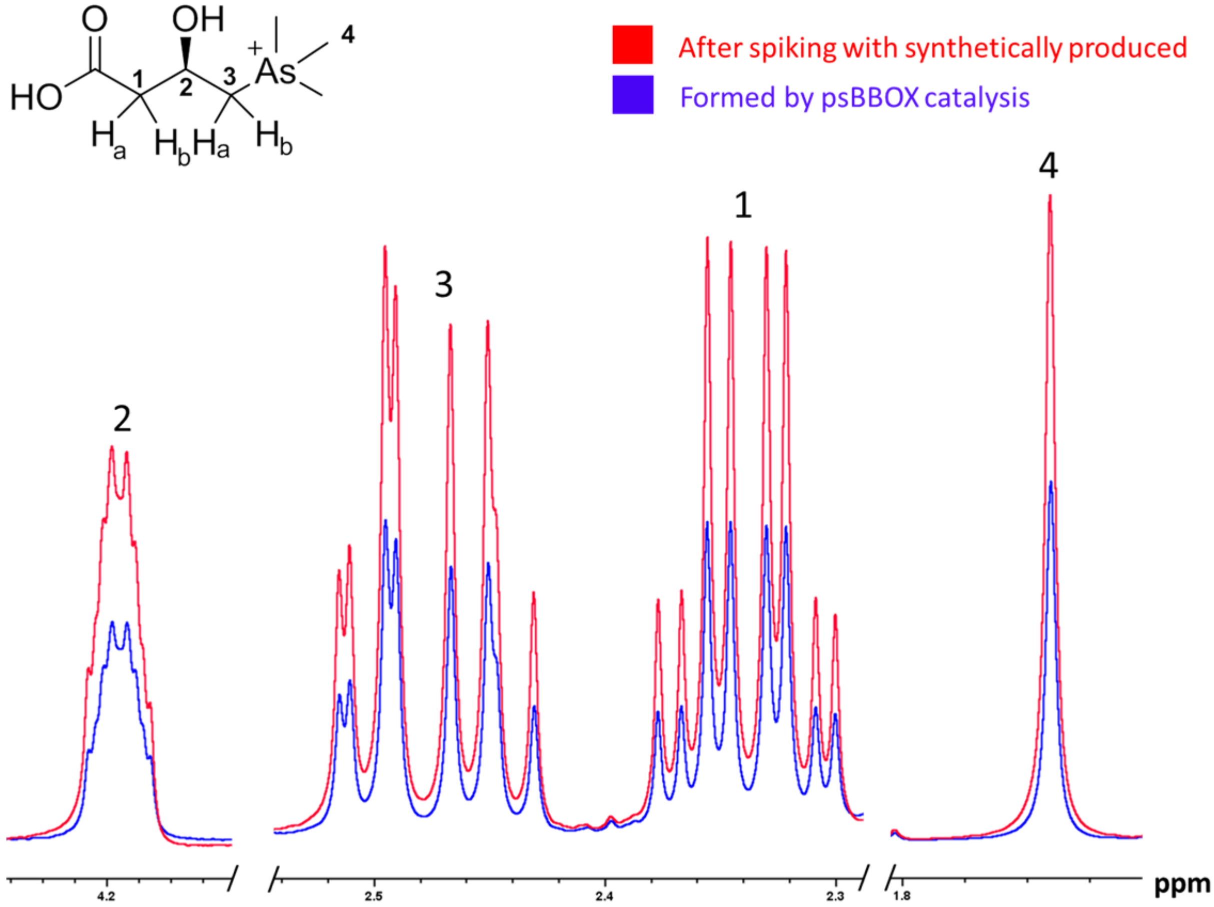

Figure S14. ^{1}H NMR assignment of hydroxylated arsa-analogue **6** before (blue) and after addition of the synthetic (3*R*)-**6** (red).

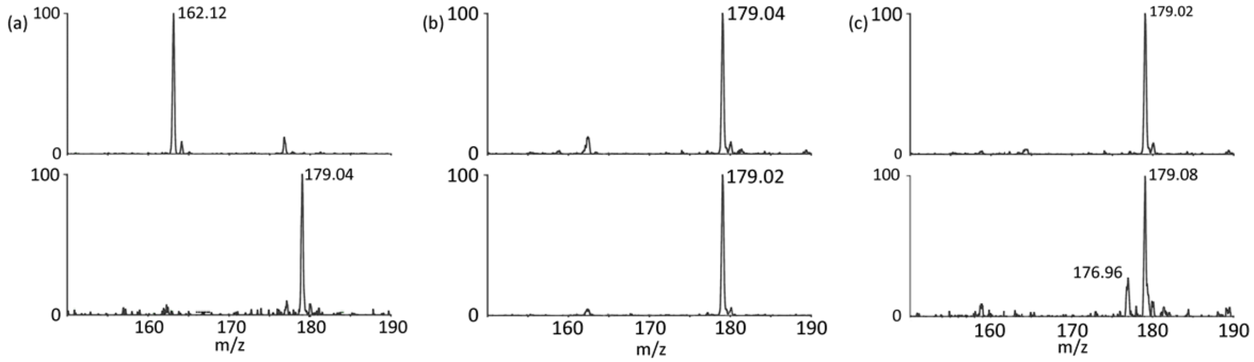

Figure S15. a) psBBOX-catalysed hydroxylation of phospha-substrate **2** in the presence of 20 µM psBBOX, b) psBBOX-catalysed hydroxylation of (3*R*)-hydroxylated phospha-analogue **5** in the presence of 20 µM psBBOX, c) psBBOX-catalysed hydroxylation of (3*S*)-hydroxylated phospha-analogue **7** in presence of 20 µM psBBOX. A typical reaction used the following conditions psBBOX (20 µM), 2OG (1.5 mM), ascorbate (5 mM), $FeSO_4$ (100 µM) and substrate (200 µM) in Tris buffer pH 7.5. Reactions were quenched with acetonitrile (80% v/v final concentration) after 1 hour incubation at 23 °C. Top panel = starting substrate, bottom panel = psBBOX-catalysed reaction.

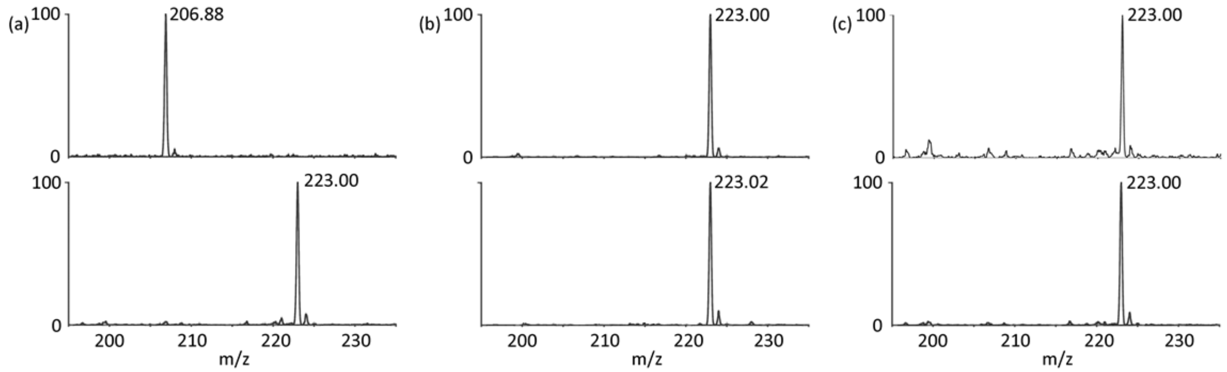

Figure S16. a) psBBOX-catalysed hydroxylation of arsa-substrate **3** in presence of 20 µM psBBOX, b) psBBOX-catalysed hydroxylation of (3*R*)-hydroxylated arsa-analogue **6** in presence of 20 µM psBBOX, c) psBBOX-catalysed hydroxylation of (3*S*)-hydroxylated arsa-analogue **8** in presence of 20 µM psBBOX. A typical reaction used the following conditions psBBOX (20 µM), 2OG (1.5 mM), ascorbate (5 mM), $FeSO_4$ (100 µM) and substrate (200 µM) in Tris buffer pH 7.5. Reactions were quenched with acetonitrile (80% v/v final concentration) after 1 hour incubation at 23 °C. Top panel = starting substrate, bottom panel = psBBOX-catalysed reaction.

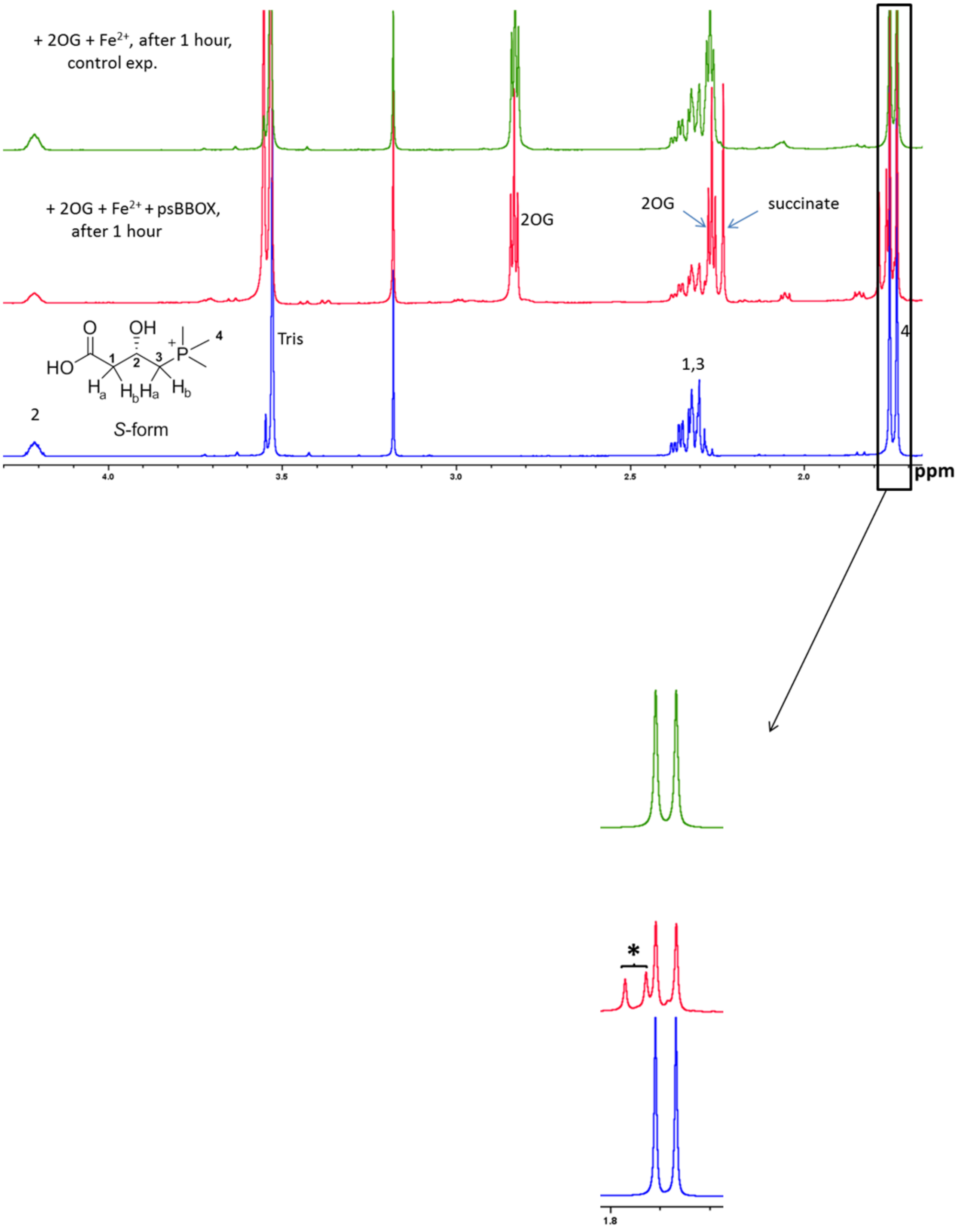

Figure S17. Hydroxylation of (3*S*)-hydroxylated phospha-analogue **7** (bottom, blue) in the presence (middle, red) and absence (top, green) of psBBOX. The appearance of the new PMe_3^+ peak (marked with *) indicates the formation of the product, likely the corresponding 3-keto derivative of **2** (as observed by LC-MS analyses, see Fig. S15c).

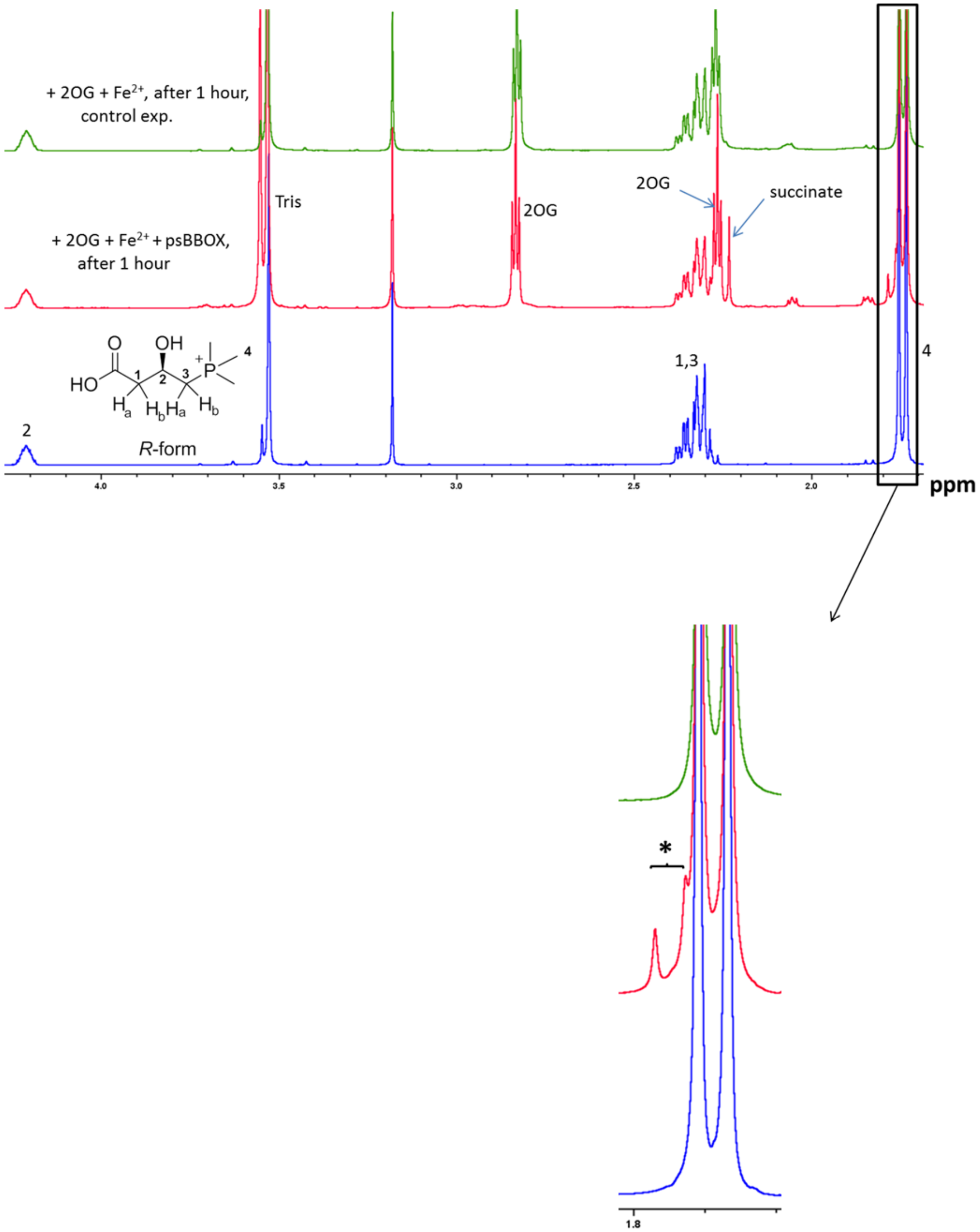

Figure S18. Hydroxylation of (3*R*)-hydroxylated phospha-analogue **5** (bottom, blue) in the presence (middle, red) and absence (top, green) of psBBOX. The appearance of the new PMe_3^+ peak (marked with *) indicates the formation of the product, likely the corresponding 3-keto derivative of **2**.

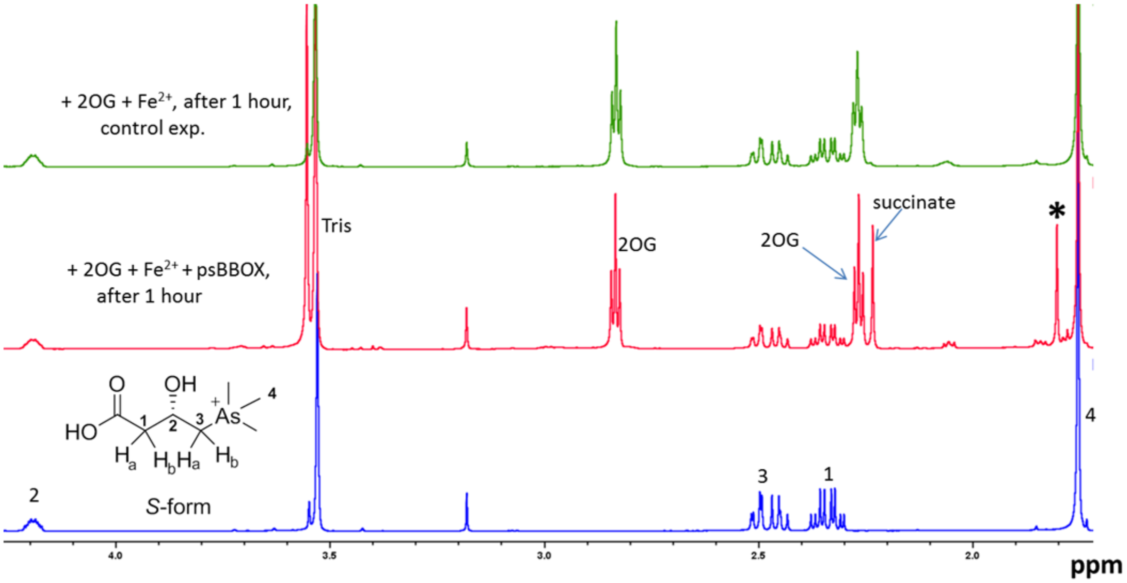

Figure S19. Hydroxylation of (3*S*)-hydroxylated arsa-analogue **8** (bottom, blue) in the presence (middle, red) and absence (top, green) of psBBOX. The appearance of the new $AsMe_3^+$ peak (marked with *) indicates the formation of the product, likely the corresponding 3-keto derivative of **3**.

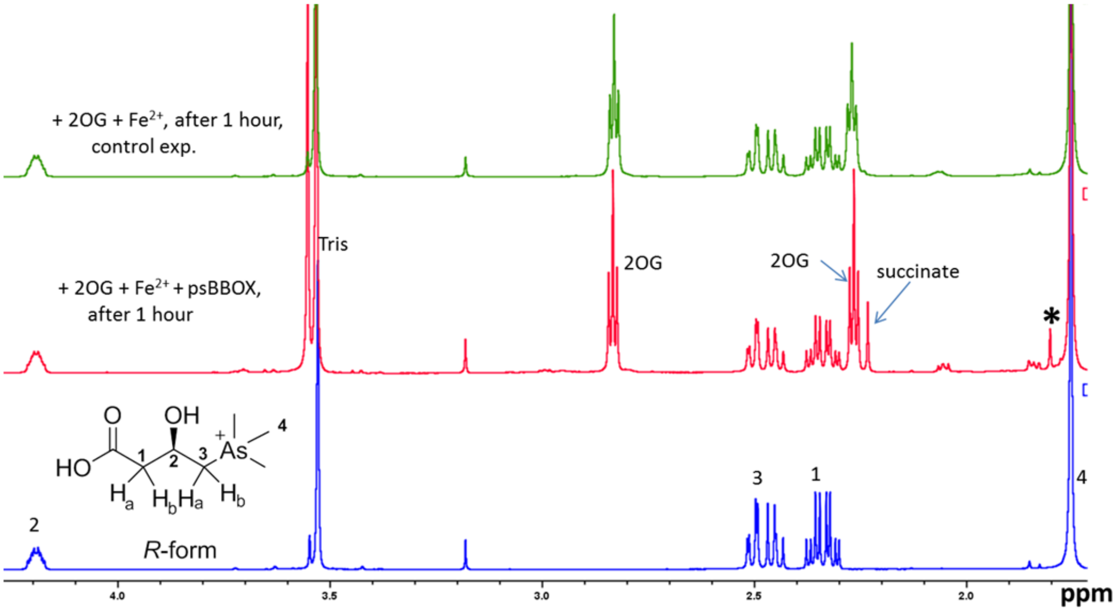

Figure S20. Hydroxylation of (3*R*)-hydroxylated arsa-analogue **6** (bottom, blue) in the presence (middle, red) and absence (top, green) of psBBOX. The appearance of the new $AsMe_3^+$ peak (marked with *) indicates the formation of the product, likely the corresponding 3-keto derivative of **3**.

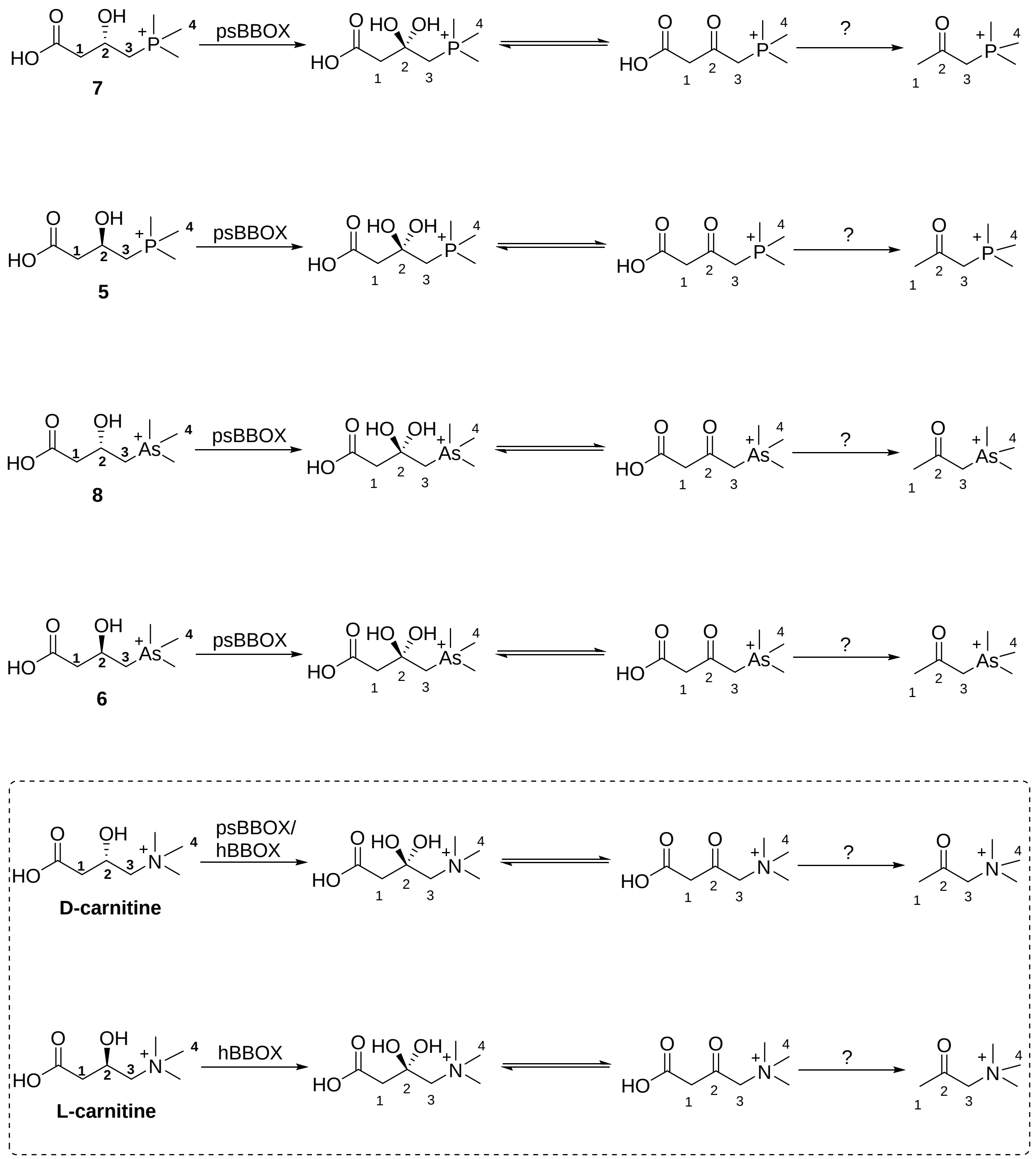

Figure S21. A schematic presentation of the plausible enzymatic and non-enzymatic pathways for the conversion of 3-hydroxylated phospha and arsa derivatives (**5**–**8**) into the corresponding 3-ketone derivatives that could eventually undergo decarboxylation reaction to yield the trimethyl(2-oxopropyl)phosphonium and trimethyl(2-oxopropyl)arsonium products. Products of the BBOX-catalysed hydroxylation of D- and L-carnitine as previously reported.[3]

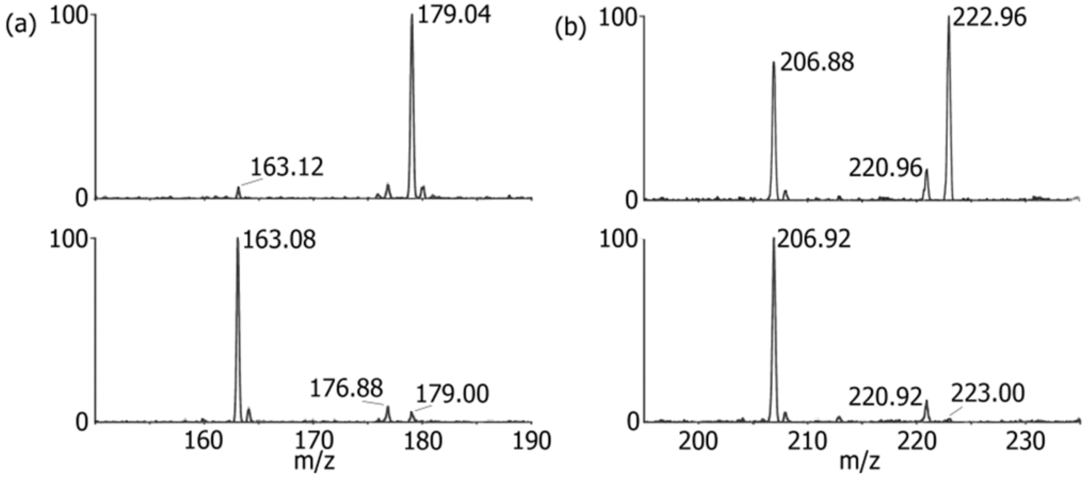

Figure S22. a) psBBOX-catalysed hydroxylation of phospha-substrate **2** under standard conditions (top) and under argon atmosphere (bottom); b) psBBOX-catalysed hydroxylation of arsa-substrate **3** under standard conditions (top) and under argon atmosphere (bottom). A typical reaction with psBBOX (2 µM), 2OG (1.5 mM), ascorbate (5 mM), $FeSO_4$ (50 µM) and substrate (200 µM). Peaks at 176.88 Da ($M+H^+$) and 220.96 Da ($M+2Na^+$) derive from sodium ascorbate. Reactions were quenched with acetonitrile (80% v/v final concentration) after 1 hour incubation at 23 °C.

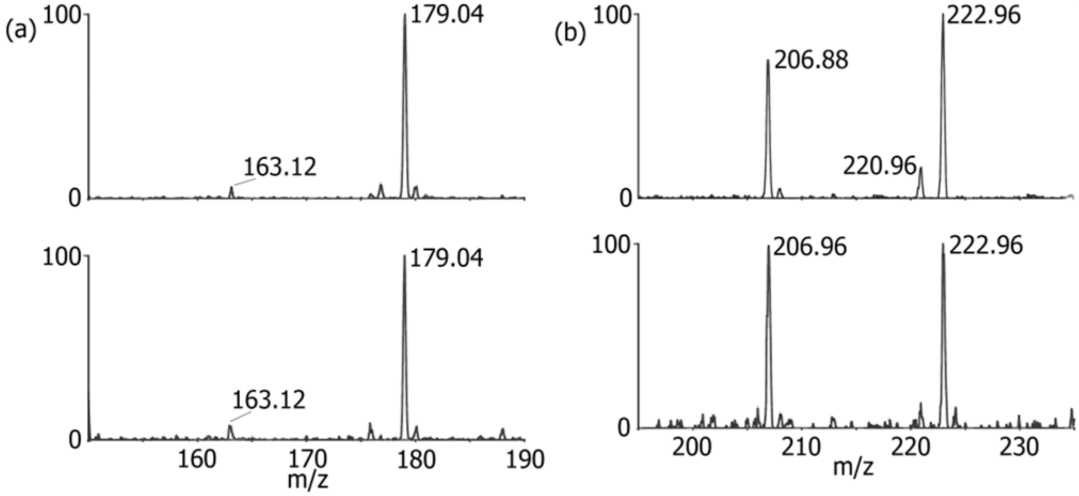

Figure S23. a) psBBOX-catalysed hydroxylation of phospha-substrate **2** under standard conditions in $H_2{}^{16}O$ (top) and in $H_2{}^{18}O$ (bottom); b) psBBOX-catalysed hydroxylation of arsa-substrate **3** under standard conditions in $H_2{}^{16}O$ (top) and in $H_2{}^{18}O$ (bottom). A standard reaction was carried out with the following conditions psBBOX (2 µM), 2OG (1.5 mM), ascorbate (5 mM), $FeSO_4$ (50 µM) and substrate (200 µM) in Tris buffer pH 7.5. Reactions were quenched with acetonitrile (80% v/v final concentration) after 1 hour incubation at 23 °C.

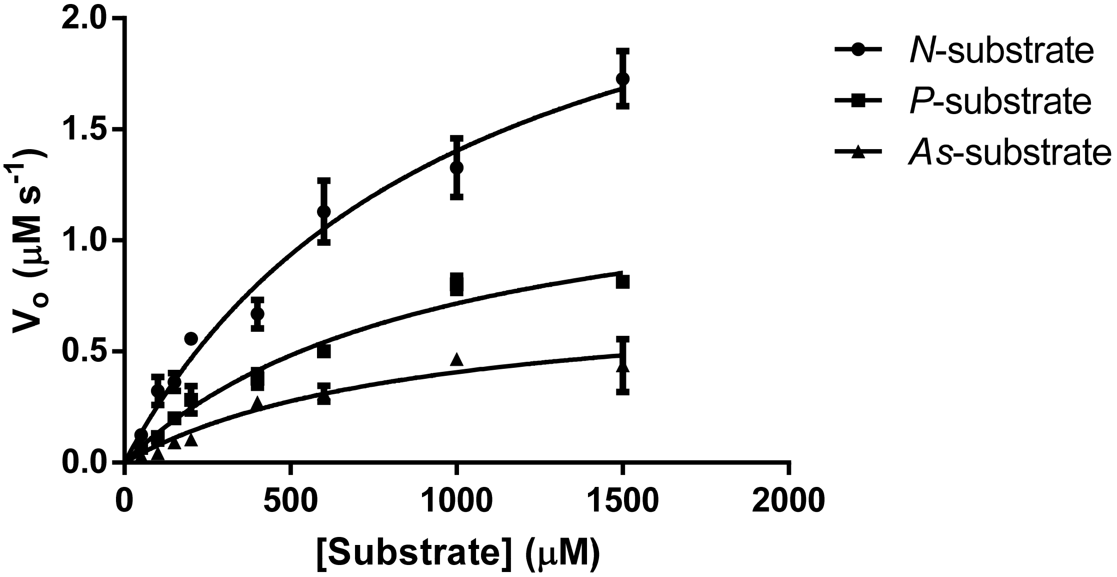

Figure S24. Plots of the kinetic experiments conducted using MS. Conditions used in these experiments are: psBBOX (400 nM), 2OG (1.5 mM), ascorbate (5 mM), $FeSO_4$ (50 µM) and the assigned substrate (50 µM – 1.5 mM) in a TRIS (20 mM), NaCl (200 mM) buffer at pH 7.5. Reactions were quenched with acetonitrile (80% v/v final concentration) after 1 minute incubation at 23 °C.

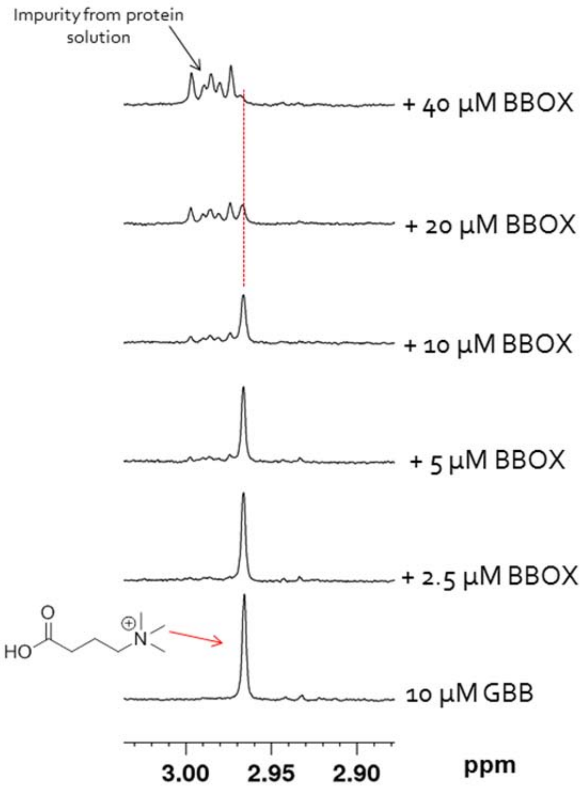

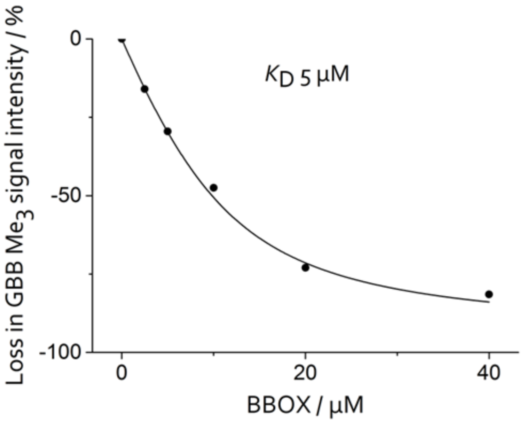

Figure S25. Measurements of the binding dissociation constant (K_D) of γBB **1** by relaxation edited (CPMG) ^{1}H NMR experiments (part of spectra shown), monitoring the attenuation of the Me_3 signal of GBB as a function of BBOX concentration (top image). The bottom image shows the fitting curve of the titration data points using Origin Pro. A K_D of 5 µM was obtained. The assay mixture contains 10 µM γBB, 1 mM 2OG, 300 µM $ZnCl_2$ in 50 mM Tris-D_{11} buffer, pH 7.5 in 90% H_2O and 10% D_2O. The filter time used in CPMG experiments was 32 ms. The CPMG ^{1}H NMR experiments were recorded to selectively remove the broad resonances of psBBOX, hence to integrate the γBB signals without interference from psBBOX resonances.

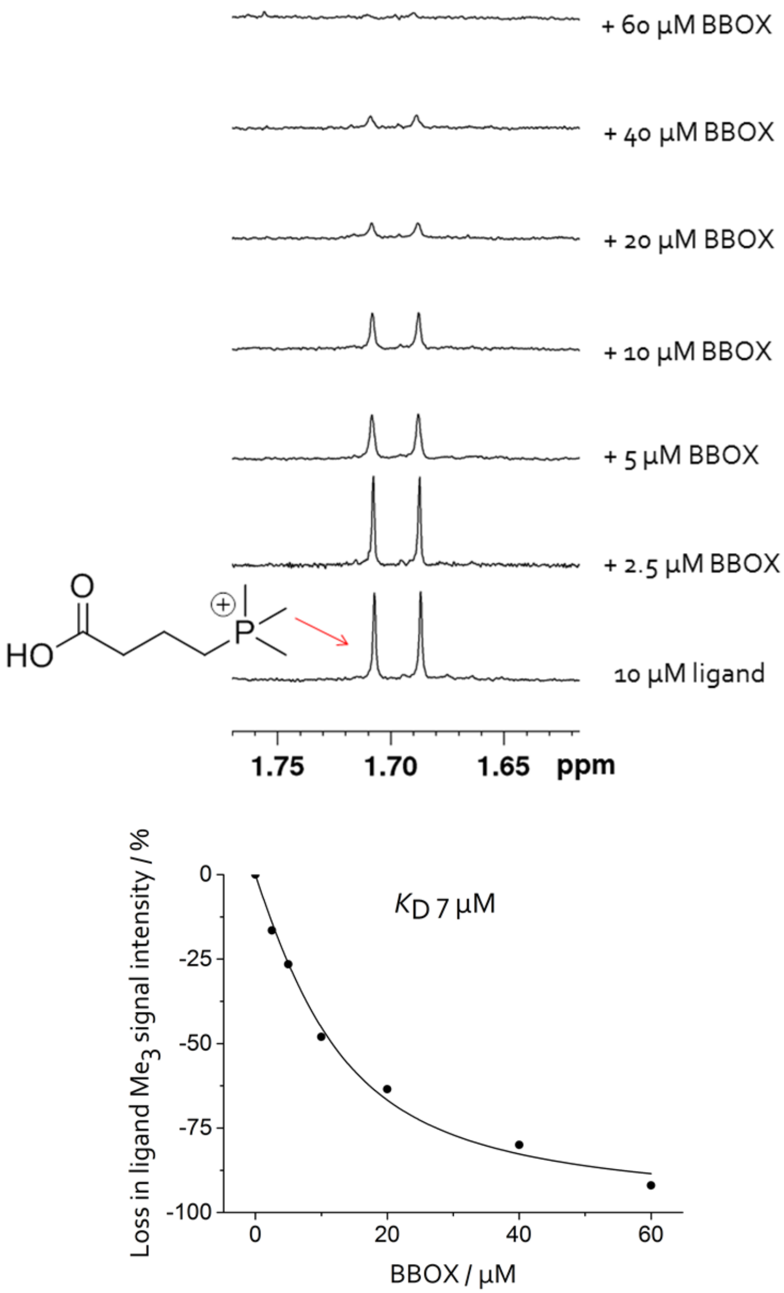

Figure S26. Measurements of the binding dissociation constant (K_D) of the phospha-substrate **2** by relaxation edited (CPMG) ^{1}H NMR experiments (part of the spectra shown) monitoring the attenuation of the Me_3 signal of the ligand as a function of BBOX concentration (top image). The bottom image shows the fitting curve of the titration data points using Origin Pro. A K_D of 7 μM was obtained. The assay mixture contains 10 μM **2**, 1 mM 2OG, 300 μM $ZnCl_2$ in 50 mM Tris-D11 buffer, pH 7.5 in 90% H_2O and 10% D_2O. The filter time used in CPMG experiments was 32 ms.

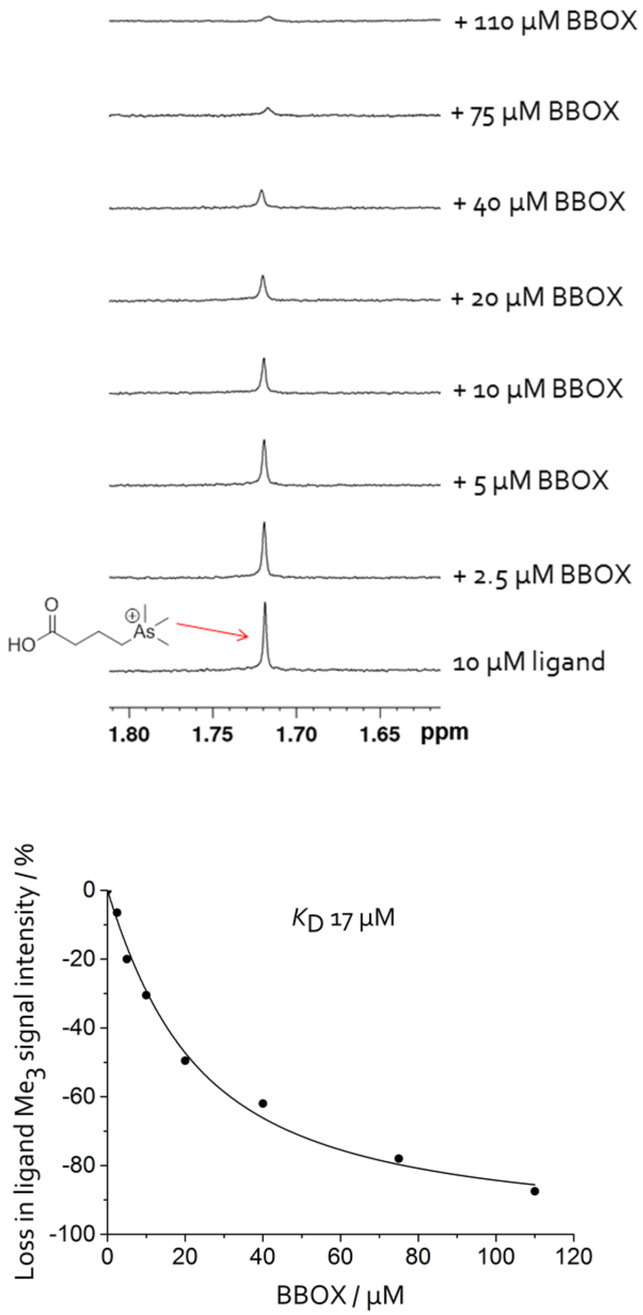

Figure S27. Measurements of the binding dissociation constant (K_D) of the arsa-substrate **3** by relaxation edited (CPMG) ^{1}H NMR experiments (part of the spectra shown) monitoring the attenuation of the Me3 signal of the ligand as a function of BBOX concentration (top image). The bottom image shows the fitting curve of the titration data points using Origin Pro. A K_D of 17 μM was obtained. The assay mixture contains 10 μM **3**, 1 mM 2OG, 300 μM $ZnCl_2$ in 50 mM Tris-D11 buffer, pH 7.5 in 90% H_2O and 10% D_2O. The filter time used in CPMG experiments was 32 ms.

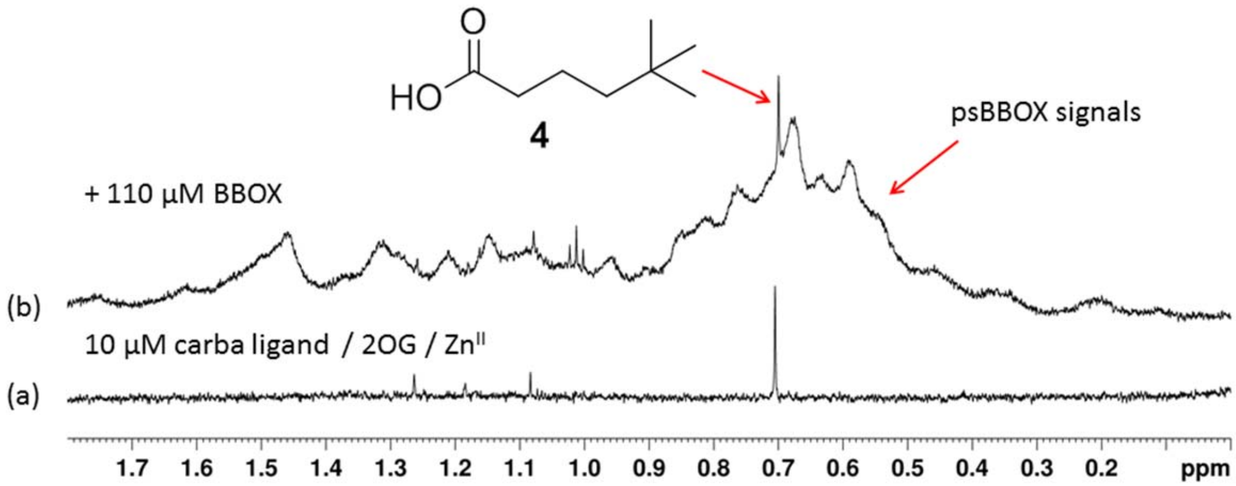

Figure S28. Monitoring the binding of carba analogue **4** to psBBOX by ^{1}H NMR direct ligand observation monitoring the broadness / attenuation of ligand NMR signals (Me_3 signal at 0.7 ppm shown) in the presence of psBBOX. (a) Spectrum (part shown) of **4** in the presence of co-factor Zn^{II} and co-substrate 2OG in the absence of psBBOX (b) in the presence of psBBOX. From spectrum (b), it is evident that the ligand (**4**) signals are not attenuated / broadened in the presence of psBBOX, demonstrating that **4** does not bind psBBOX. The assay mixture contains 10 µM **4**, 300 µM Zn^{II}, 1 mM 2OG, 110 µM psBBOX in 50 mM Tris-D11, pH 7.5 in 90% H_2O: 10% D_2O.

Table S1. Calculated CHELPG charges (in e) of the X (As, P, N and C) and its surrounding atoms in the model system for the psBBOX-substrate complex.[4]

Partial charge (CHELPG)	As_γBB	P_γBB	N_γBB	C_γBB
X= As, P, N, C	1.083874	0.982078	0.346632	0.781339
C6	-0.62055	-0.62769	-0.44605	-0.52202
C7	-0.51859	-0.54974	-0.36109	-0.48489
C8	-0.55633	-0.49209	-0.33038	-0.46134
H15	0.124747	0.16353	0.168474	0.091036
H16	0.133143	0.148607	0.168991	0.091743
H17	0.146389	0.138545	0.185918	0.093877
H18	0.153665	0.136004	0.146934	0.093287
H19	0.127262	0.199521	0.157062	0.076114
H20	0.176385	0.184089	0.151968	0.099502
H21	0.13269	0.125163	0.258579	0.087137
H22	0.174563	0.157739	0.14222	0.070371
H23	0.187265	0.1833	0.106538	0.092407
H_average	0.150679	0.159611	0.165187	0.088386

3. NMR spectra

^{1}H NMR and ^{13}C NMR spectra of (4)

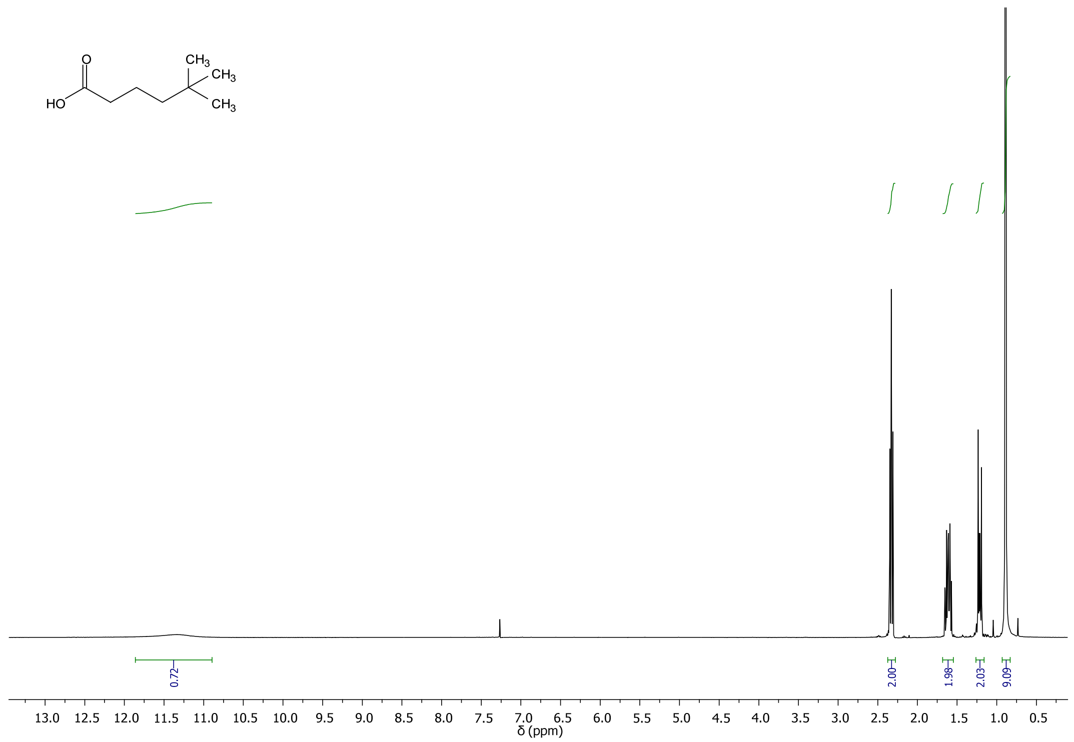

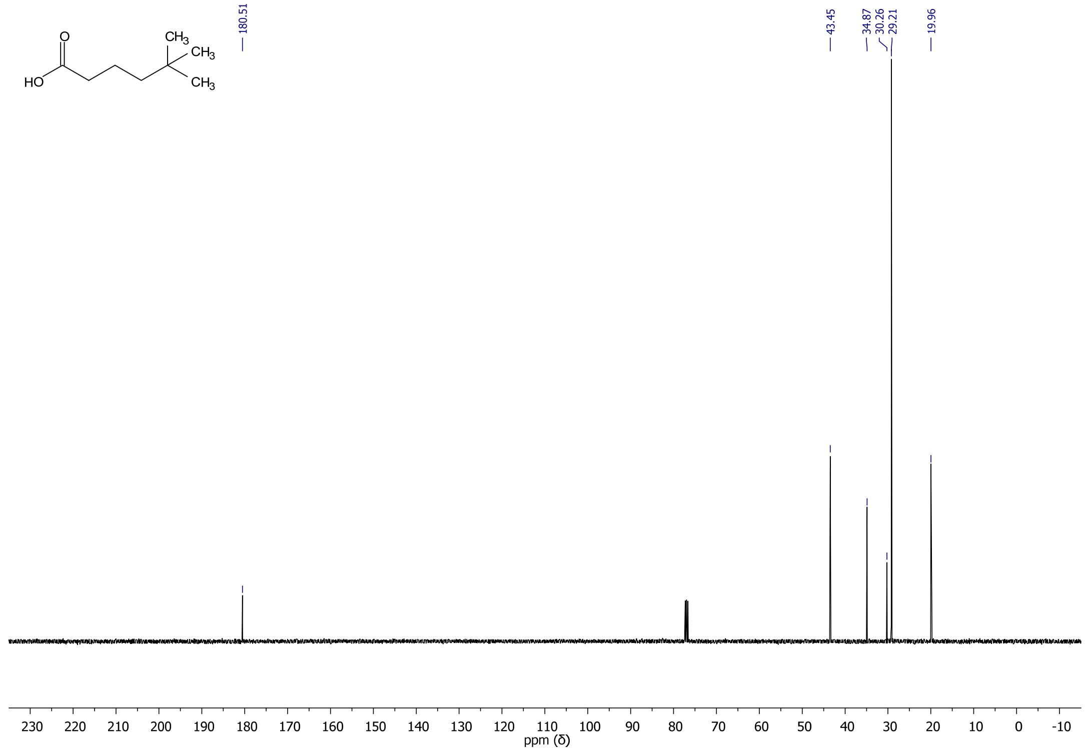

^{1}H NMR and ^{13}C NMR spectra of (9)

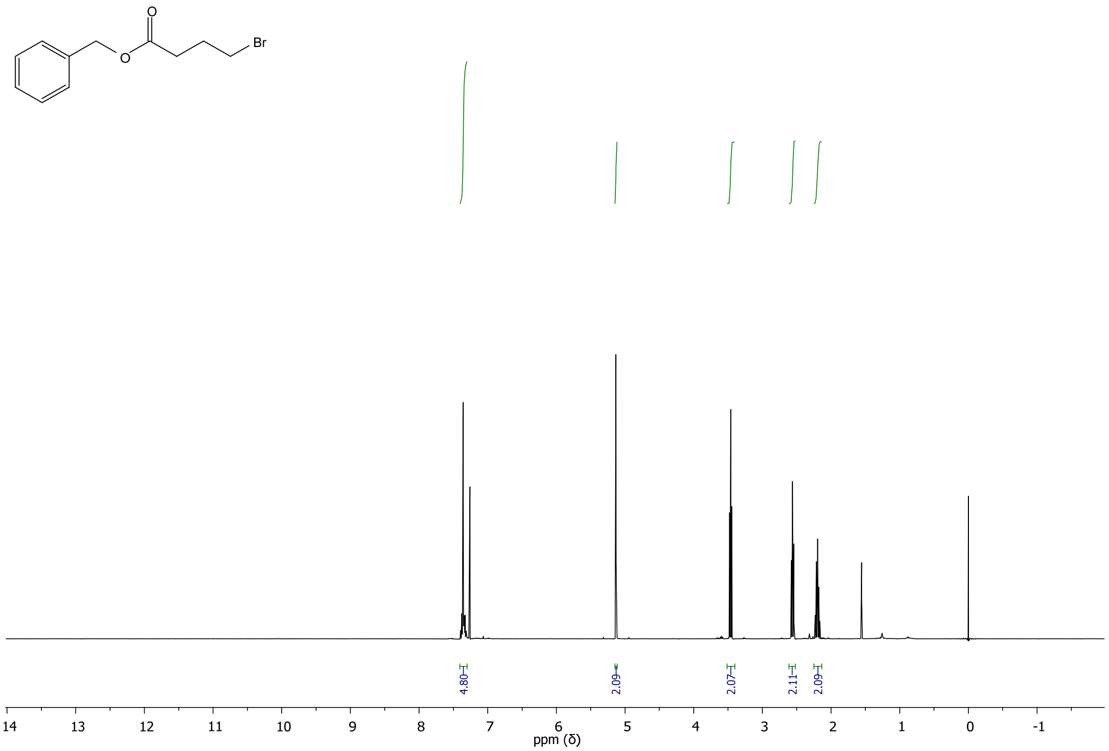

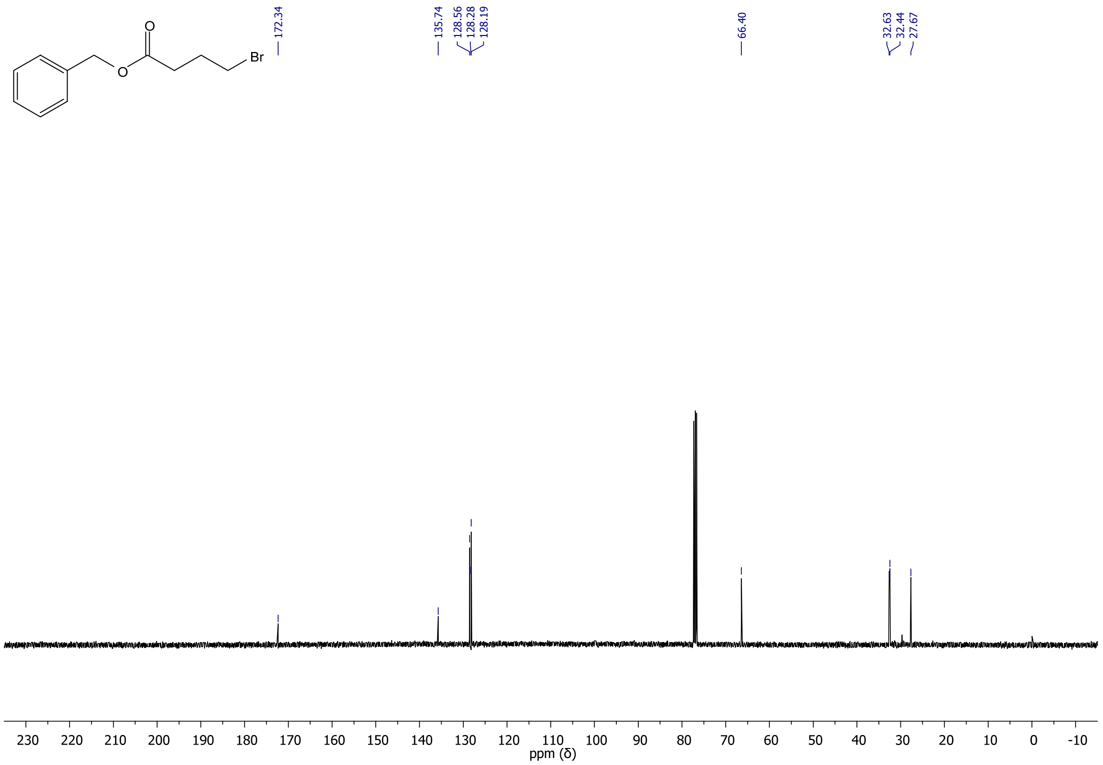

^{1}H NMR, ^{13}C NMR and ^{31}P NMR spectra of (10)

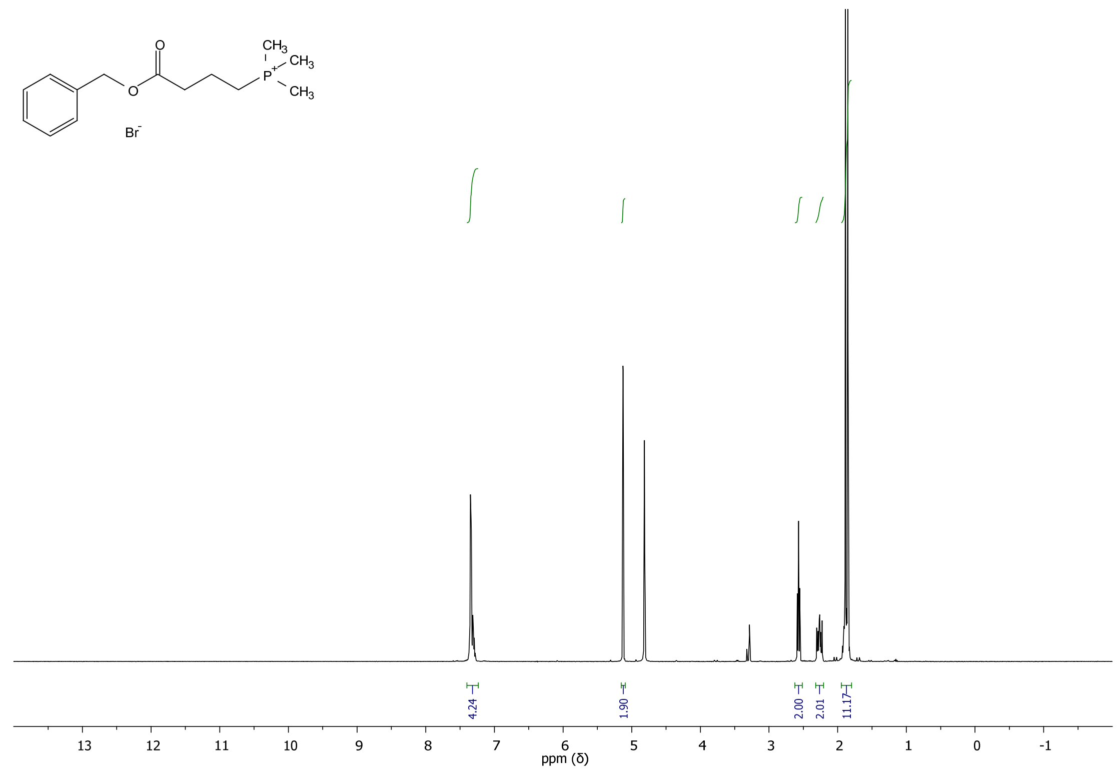

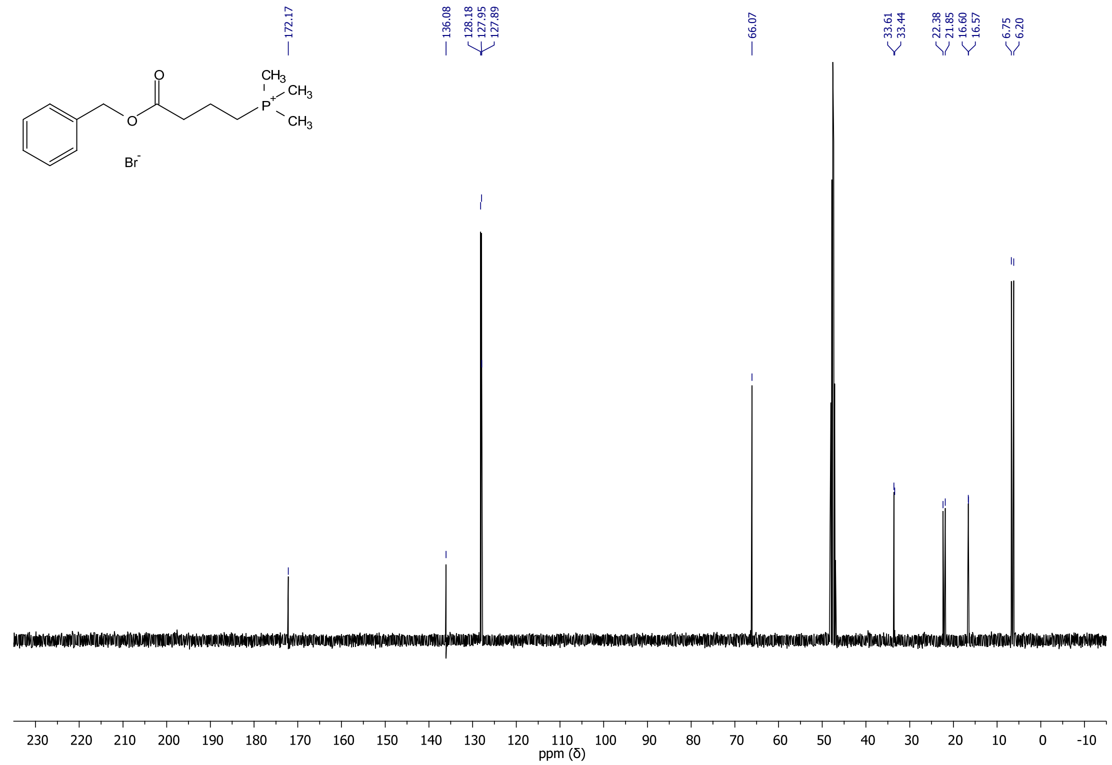

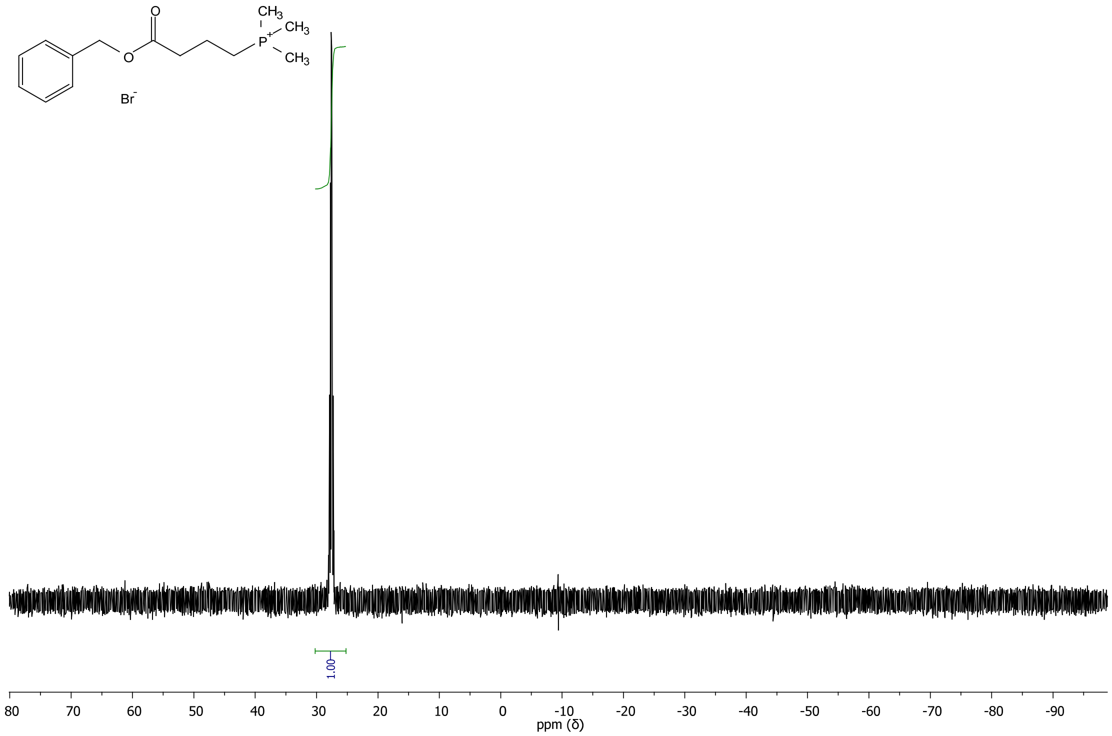

O
O
CH3
CH3
P+
CH3
Br-
1.00
80 70 60 50 40 30 20 10 0 -10 -20 -30 -40 -50 -60 -70 -80 -90
ppm (δ)

^{1}H NMR, ^{13}C NMR and ^{31}P NMR spectra of (2)

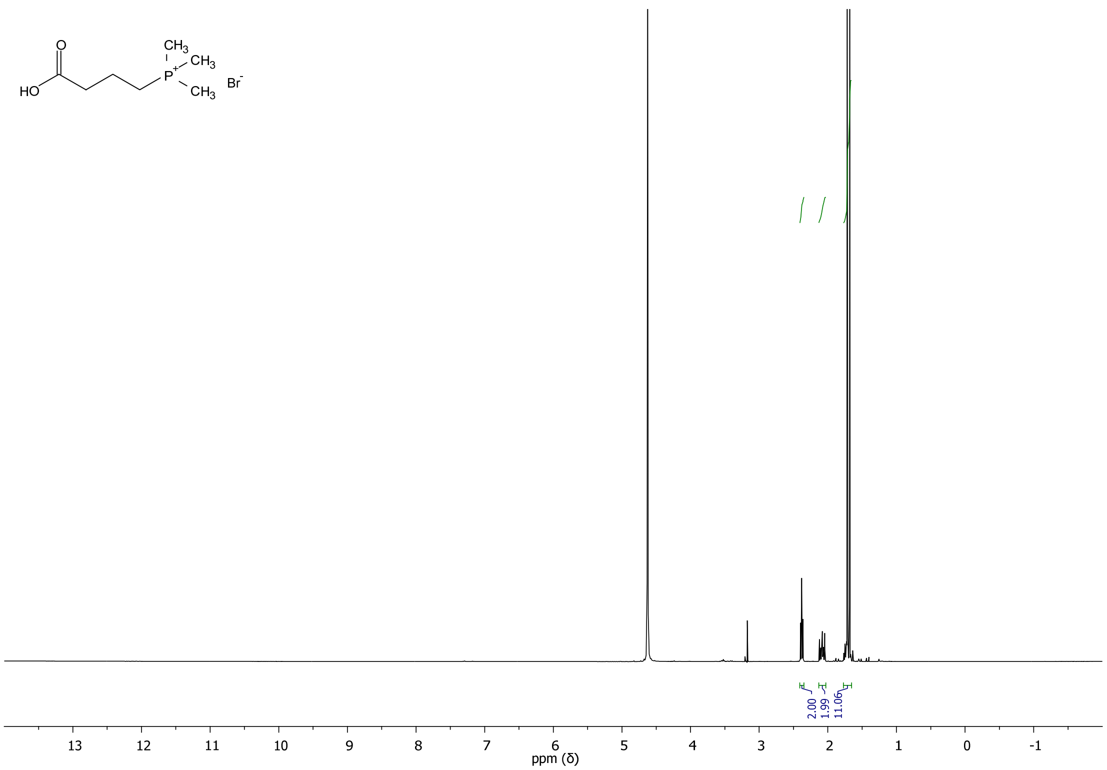

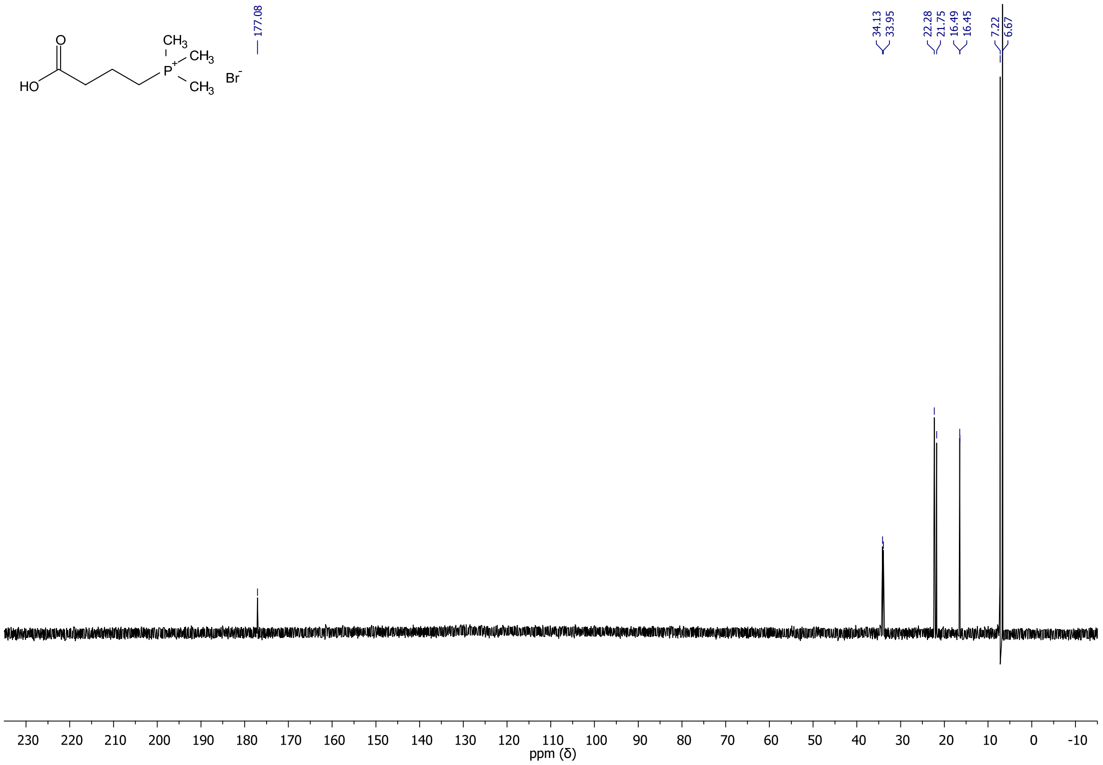

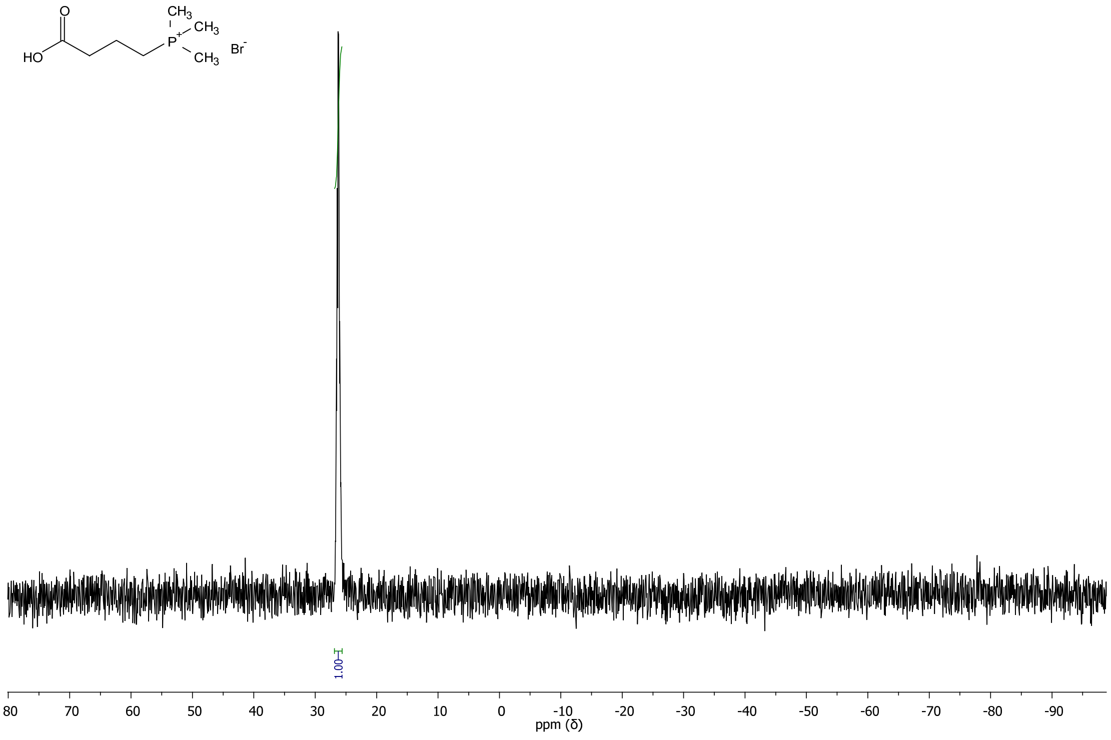

O
HO
CH_3
P+
CH_3
CH_3
Br-
1.00
80 70 60 50 40 30 20 10 0 -10 -20 -30 -40 -50 -60 -70 -80 -90
ppm (δ)

^{1}H NMR and ^{13}C NMR spectra of (11)

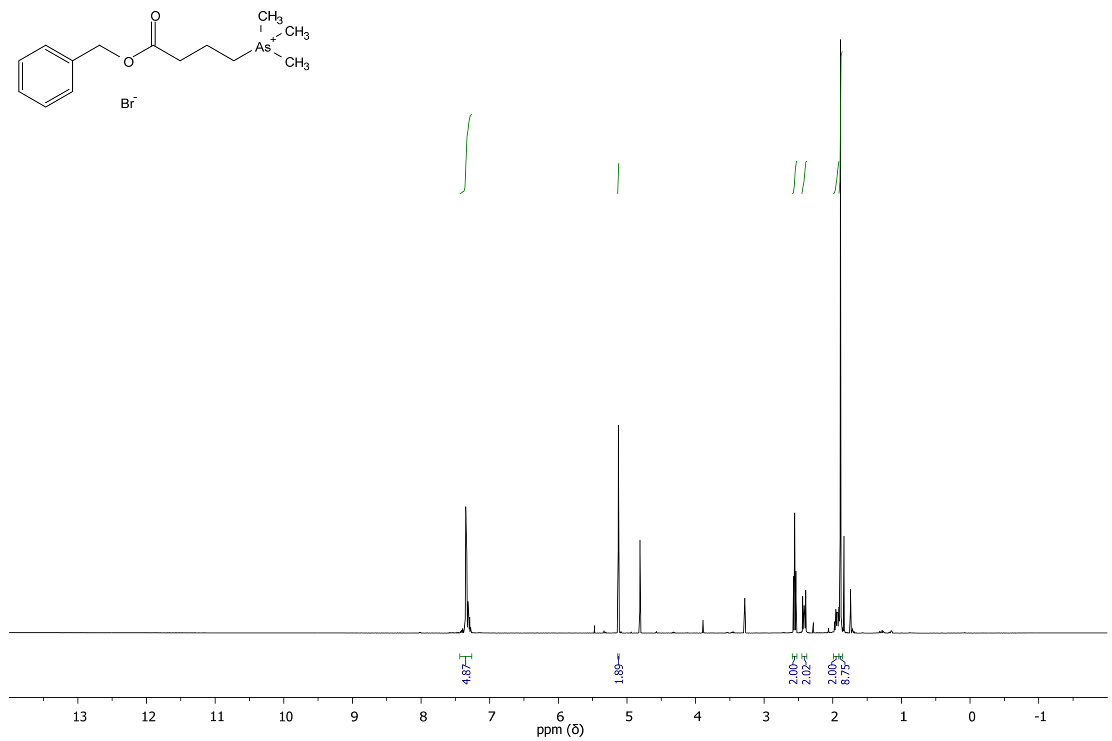

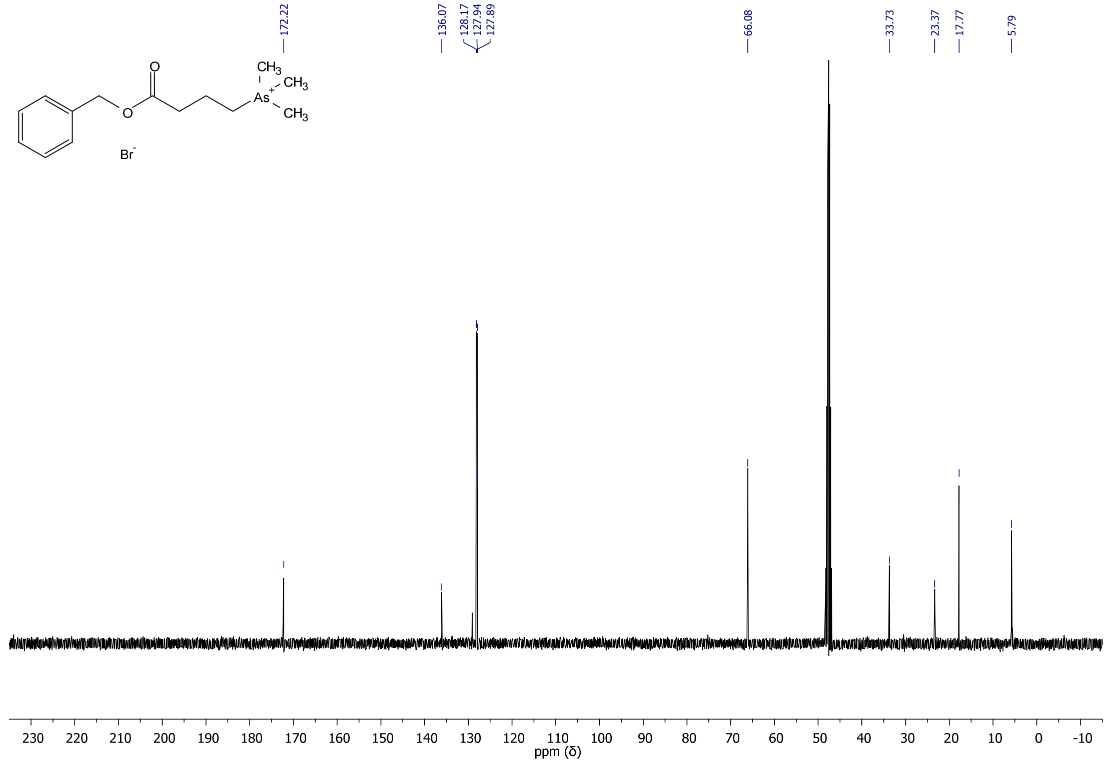

^{1}H NMR and ^{13}C NMR spectra of (3)

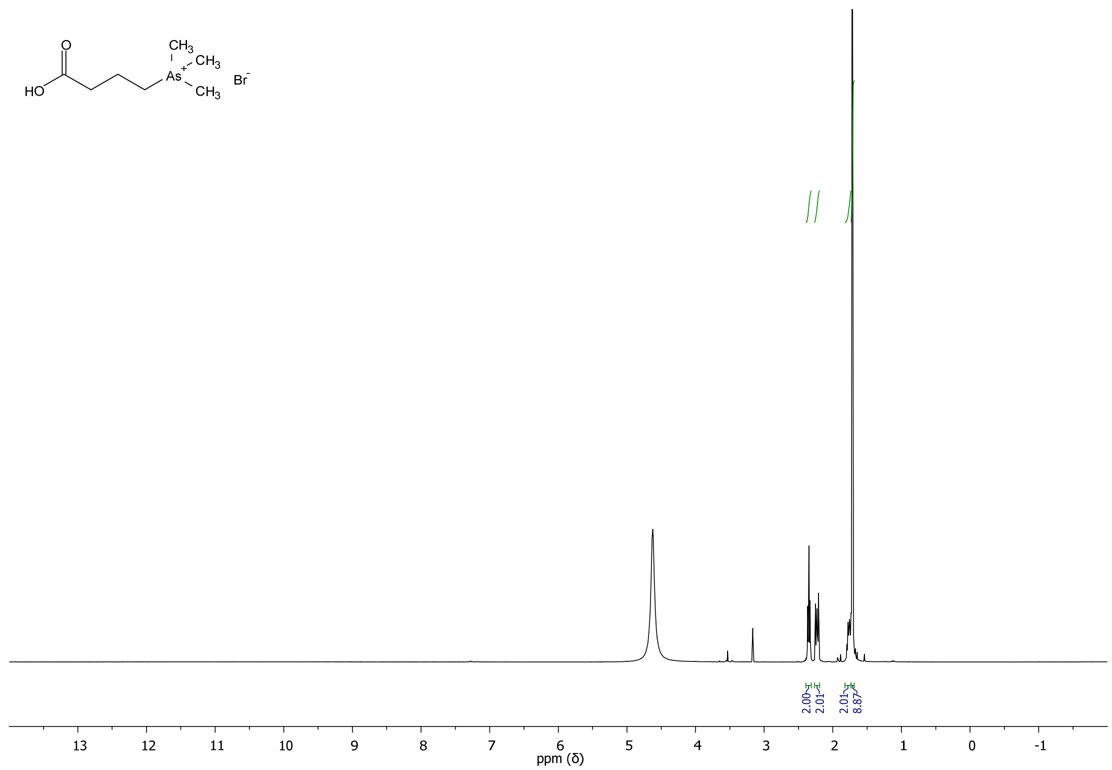

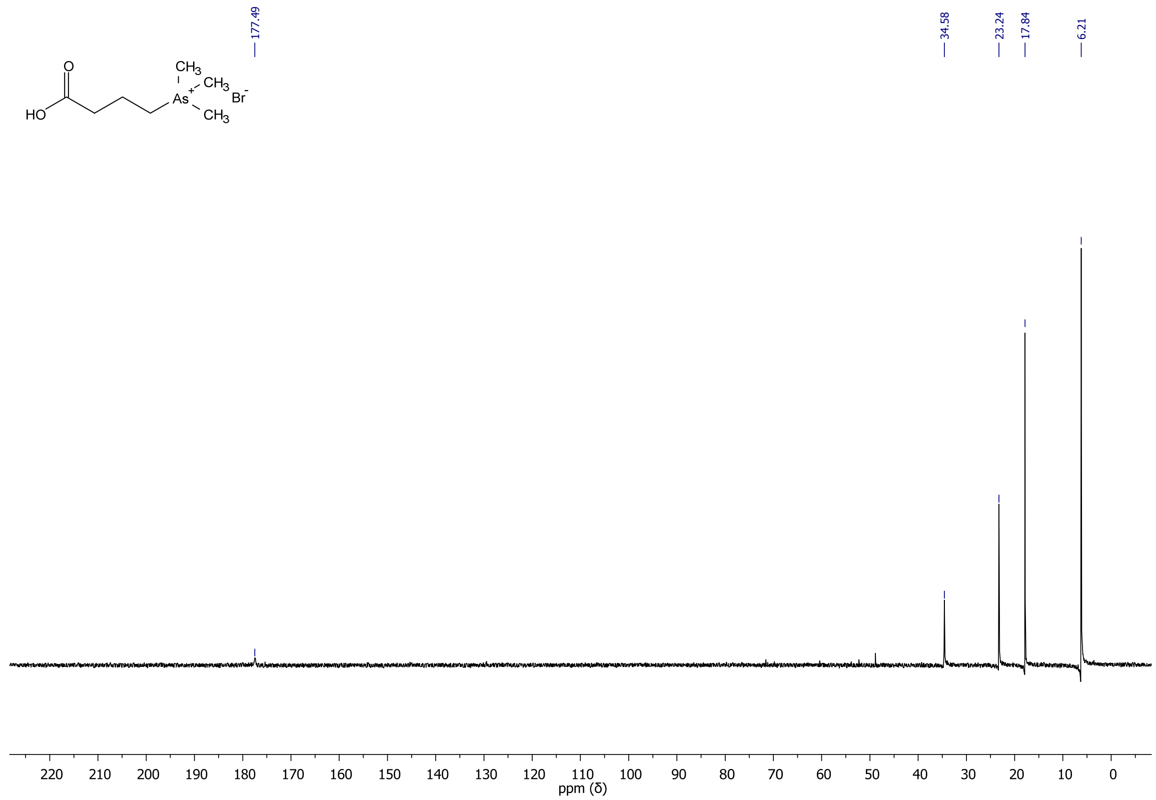

^{1}H NMR and ^{13}C NMR of (12)

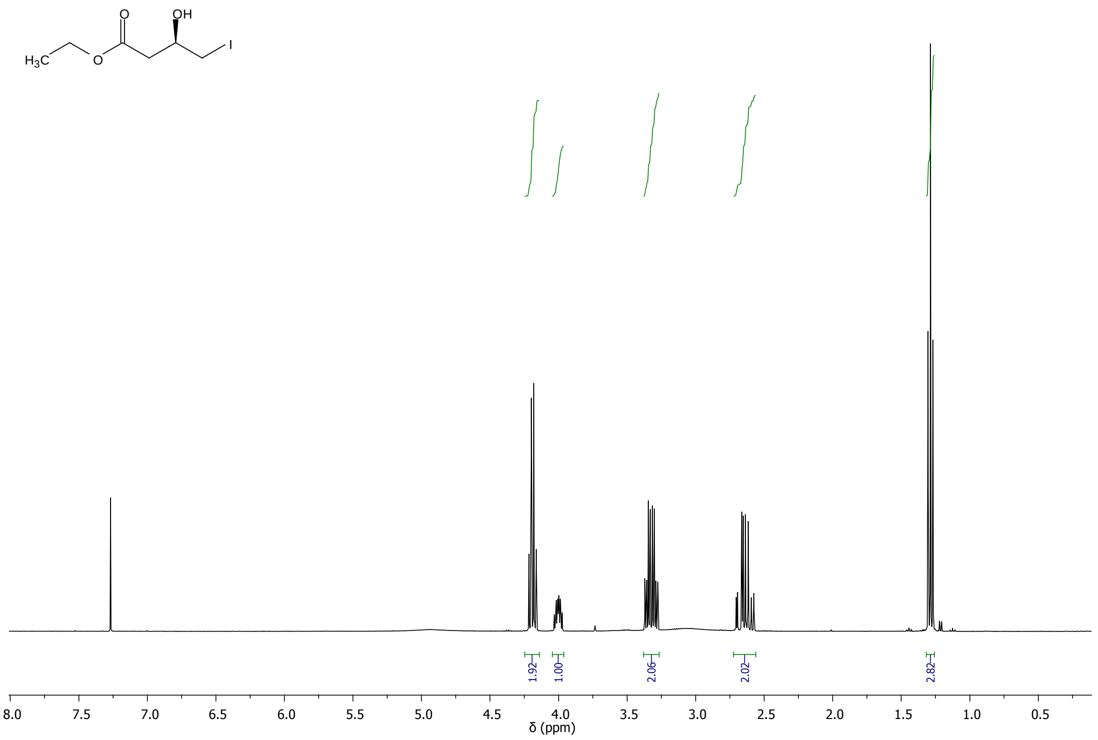

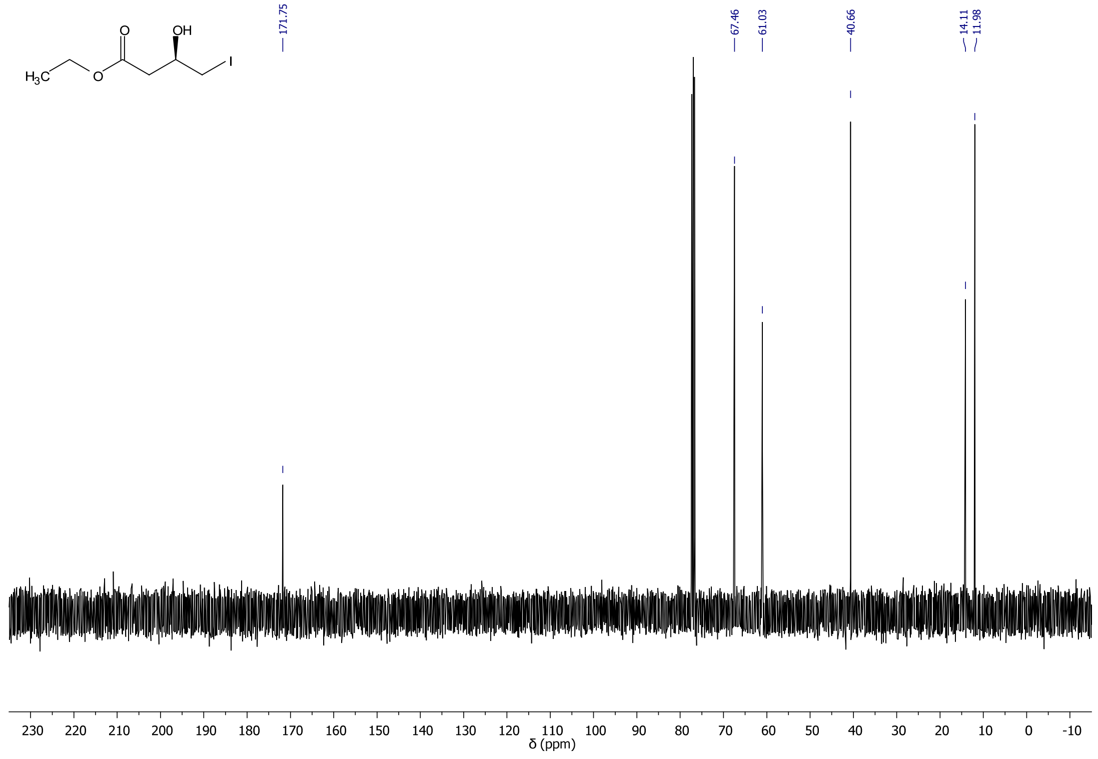

^{1}H NMR and ^{13}C NMR of (13)

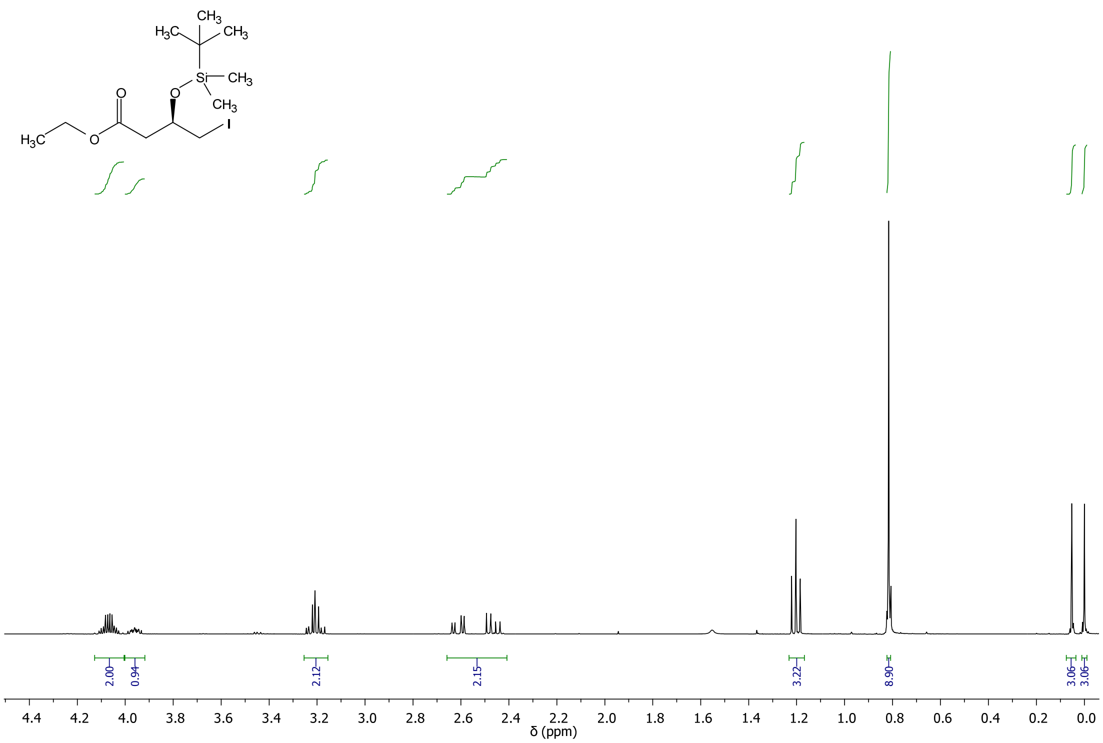

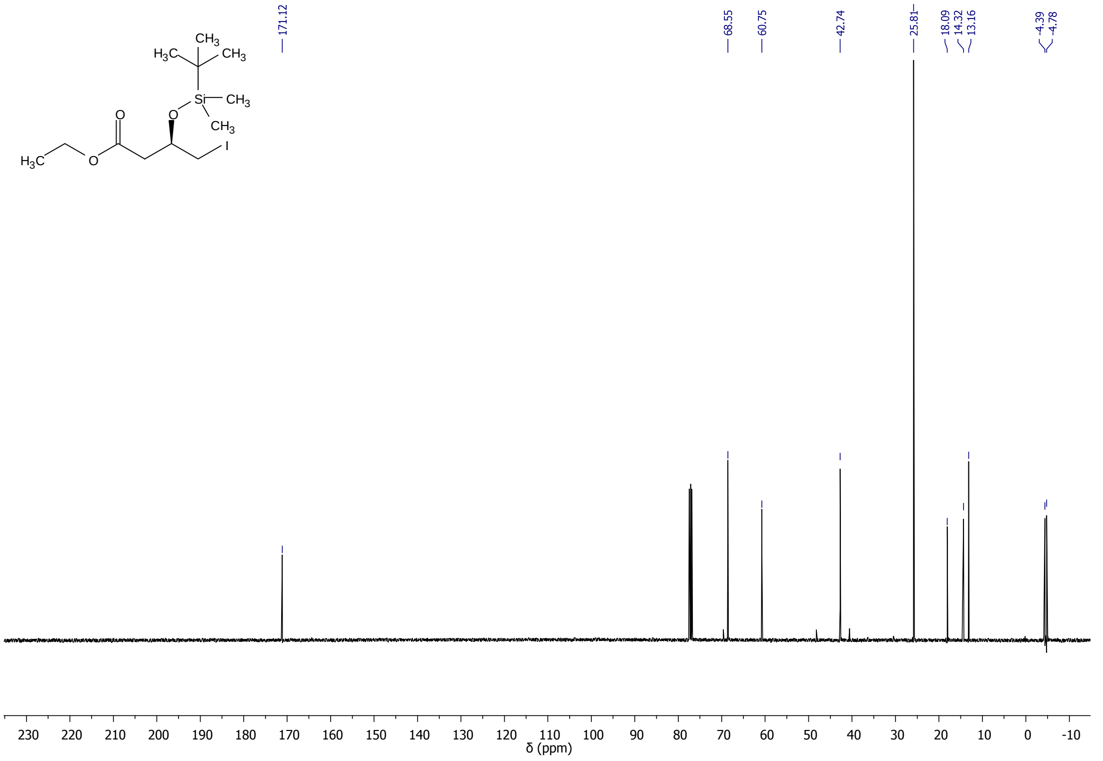

^{1}H NMR, ^{13}C NMR and ^{31}P NMR of (14)

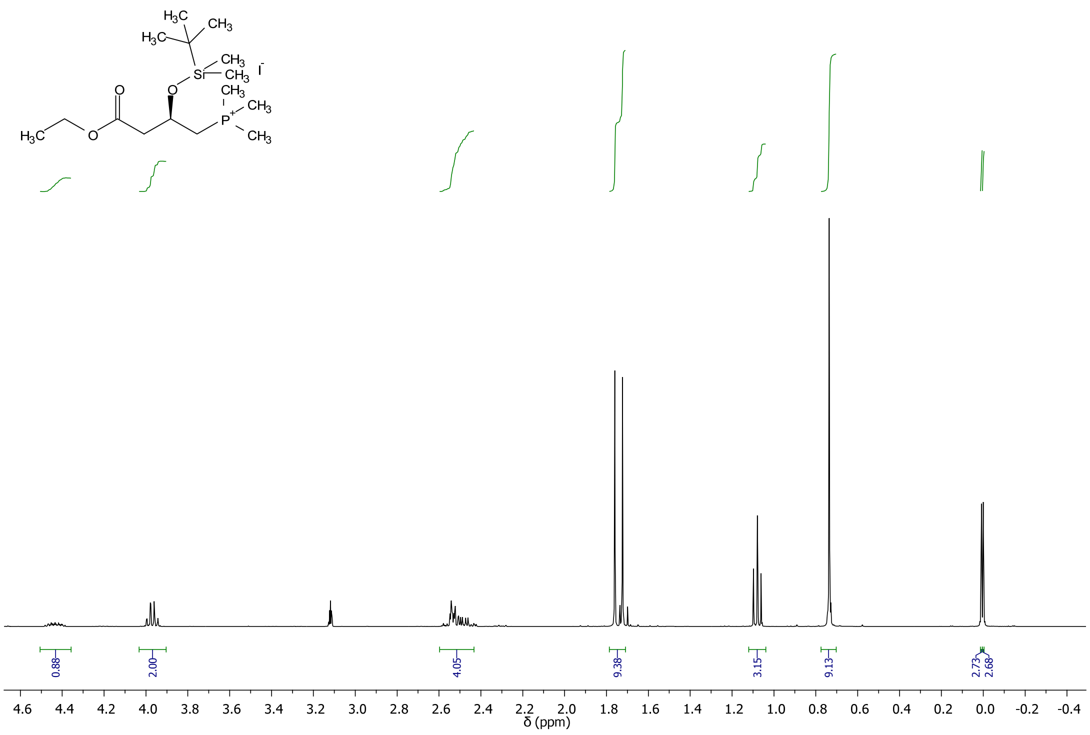

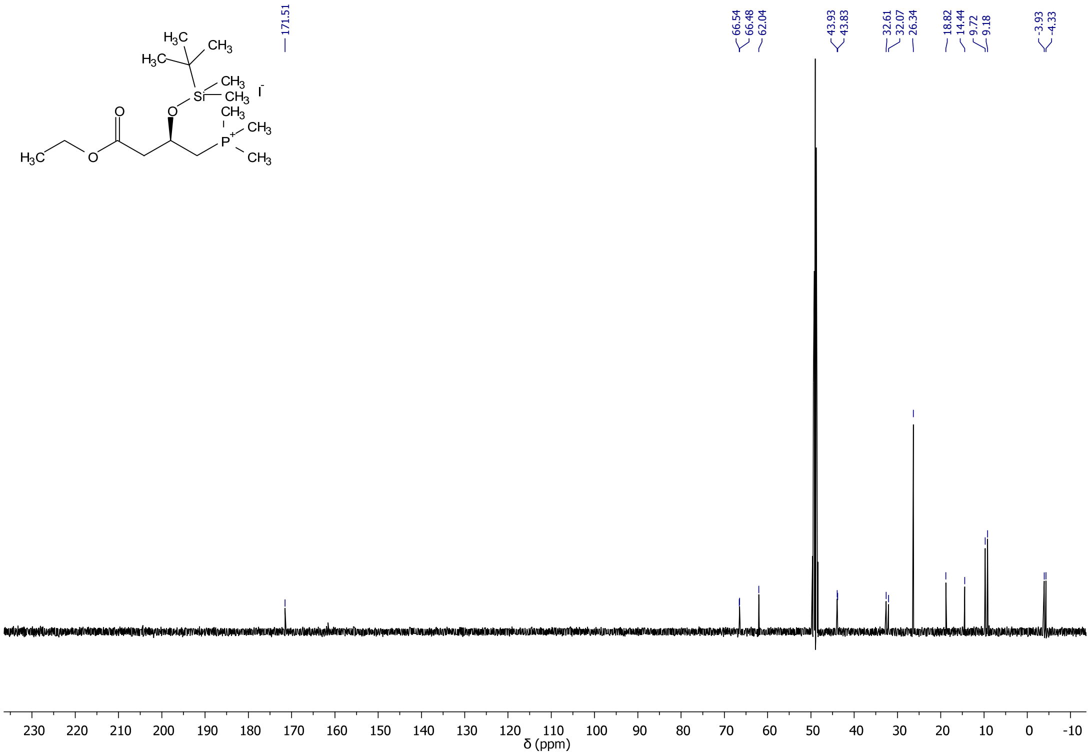

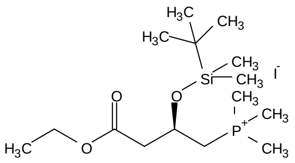

H3C
CH3
H3C
CH3
Si
CH3
I-
O
O
CH3
CH3
P+
H3C
O
CH3

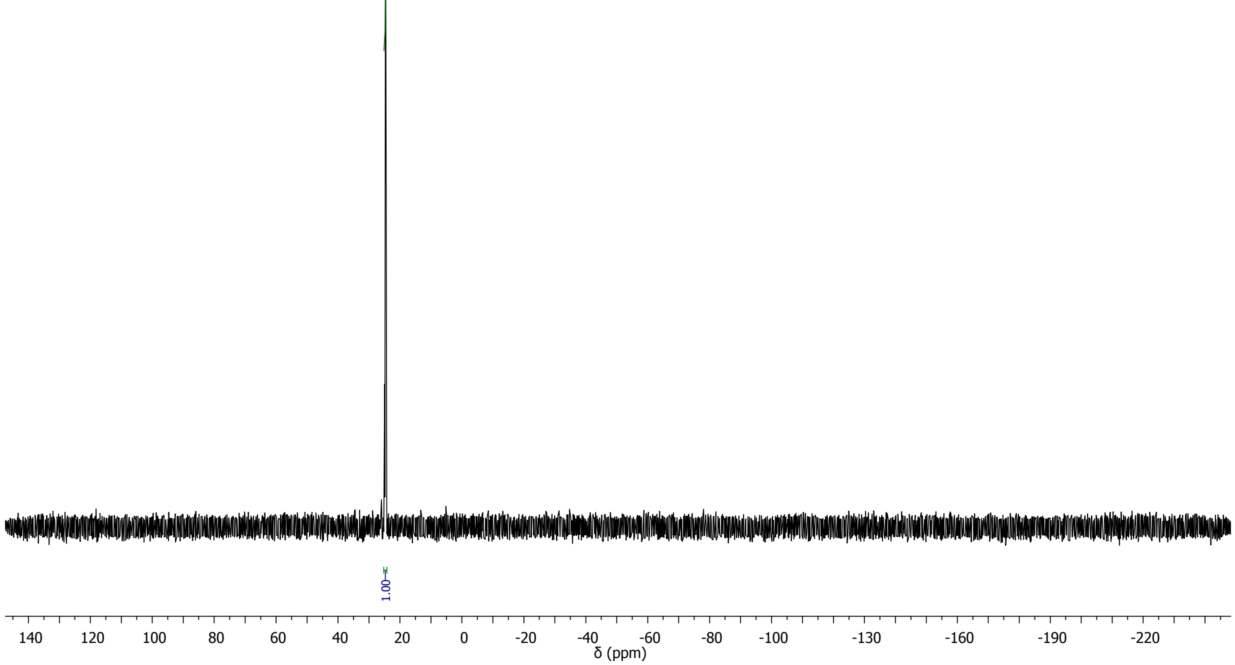

1.00
140
120
100
80
60
40
20
0
-20
-40
-60
-80
-100
-130
-160
-190
-220
δ (ppm)

^{1}H NMR, ^{13}C NMR and ^{31}P NMR of (15)

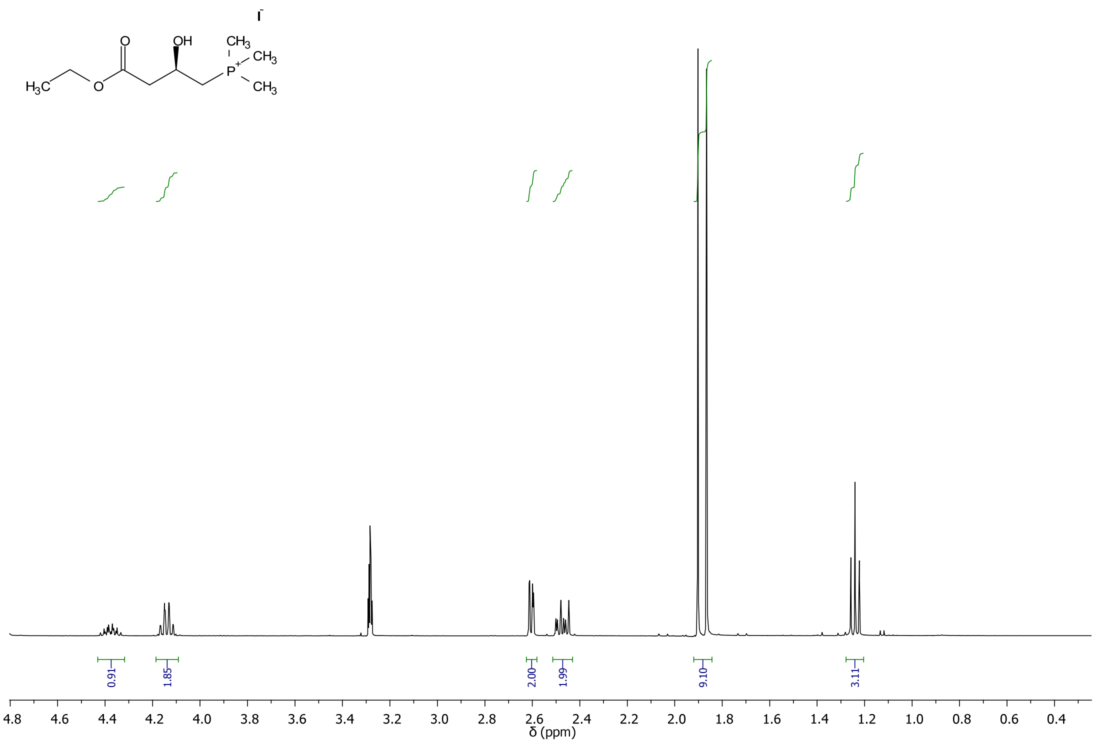

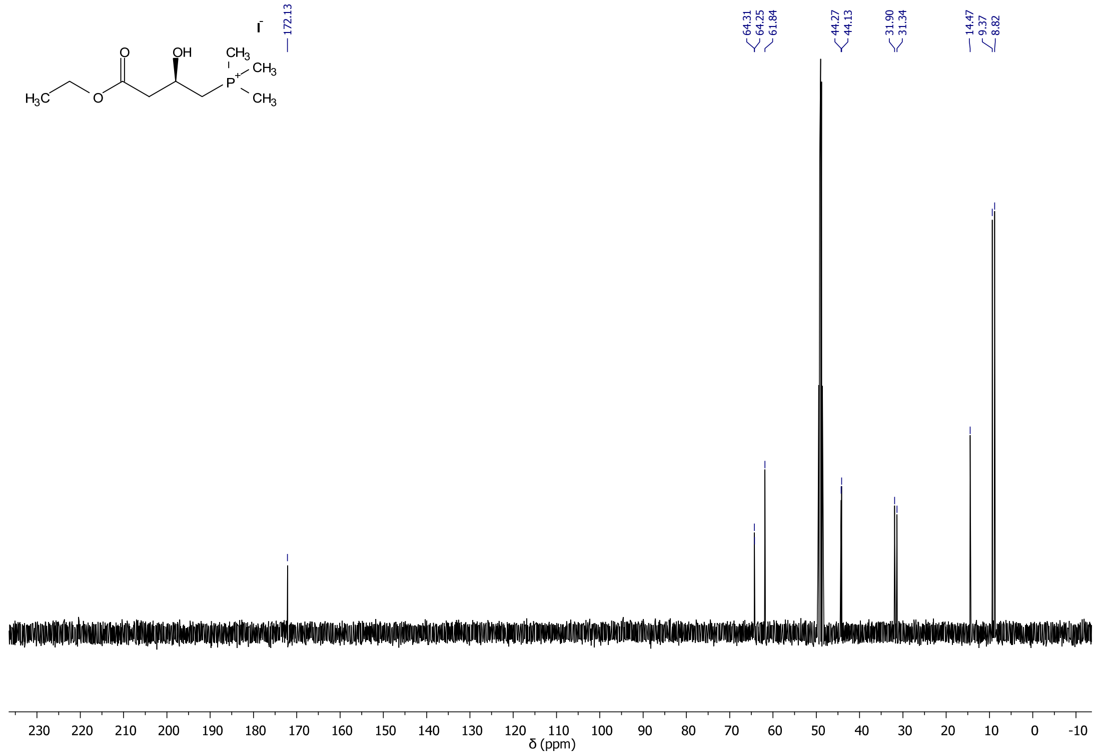

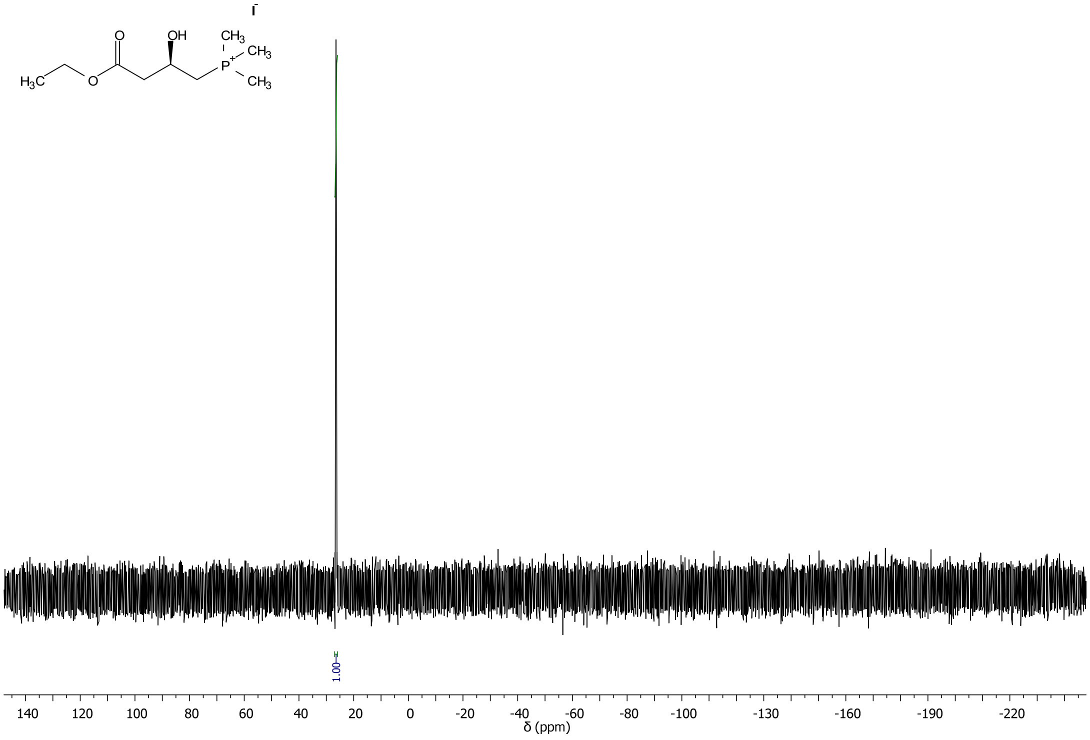
I⁻
OH
O
CH3
CH3
CH3
P+
H3C
O
1.00
140
120
100
80
60
40
20
0
-20
-40
-60
-80
-100
-130
-160
-190
-220
δ (ppm)

^{1}H NMR, ^{13}C NMR and ^{31}P NMR of (5)

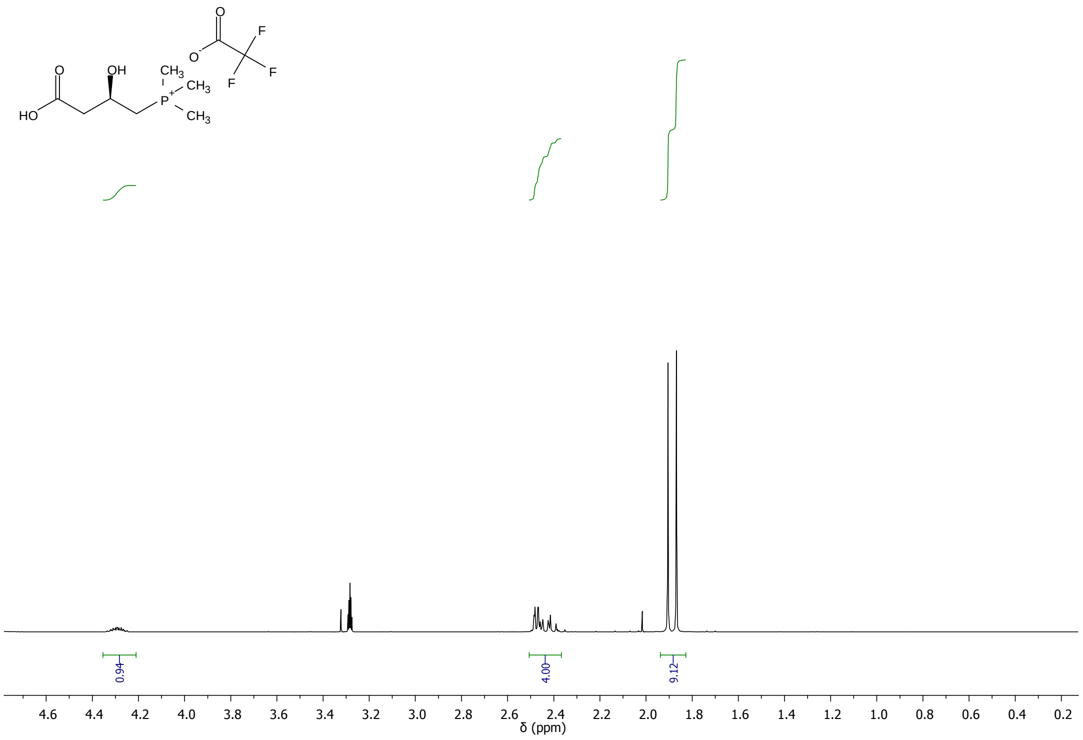

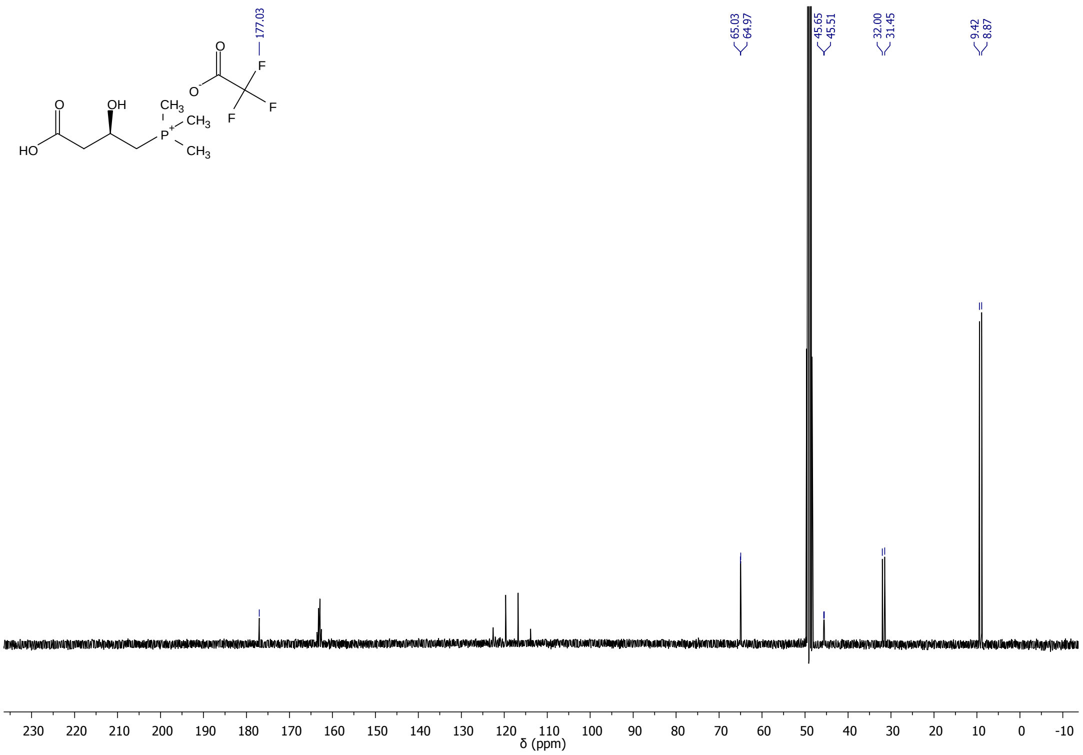

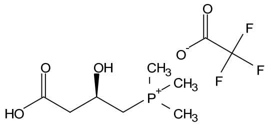
HO
O
OH
CH3
CH3
CH3
P+
O-
O
F
F
F

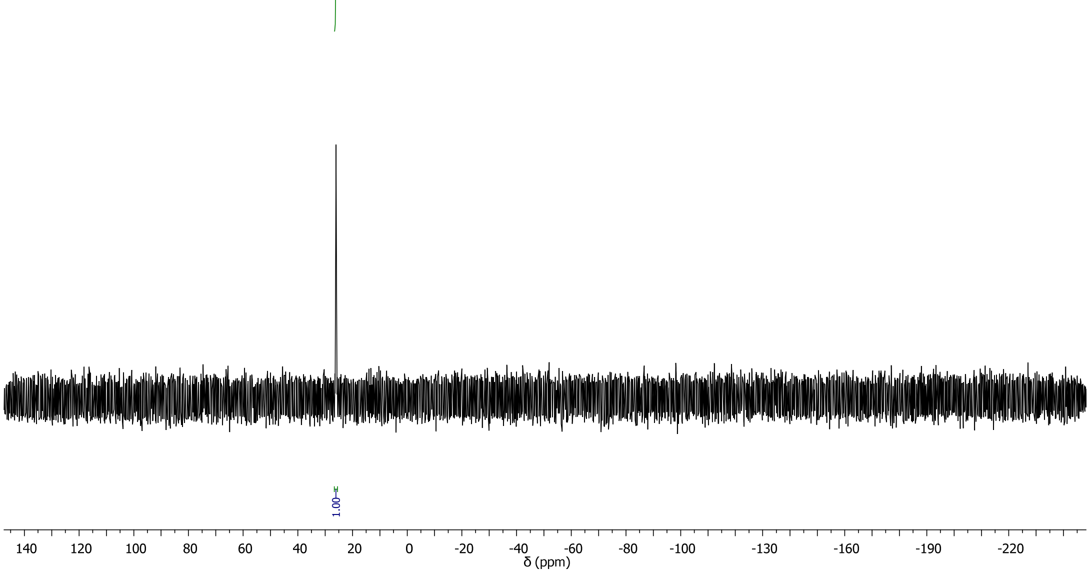
1.00
140
120
100
80
60
40
20
0
-20
-40
-60
-80
-100
-130
-160
-190
-220
δ (ppm)

^{1}H NMR and ^{13}C NMR of (16)

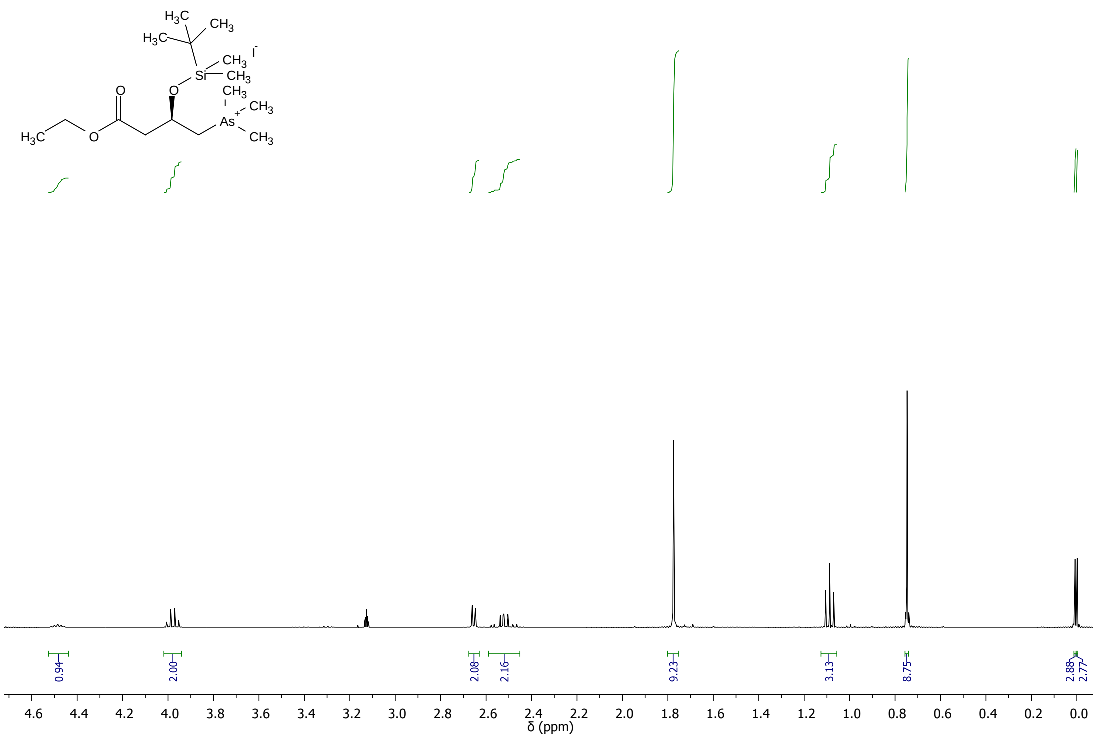

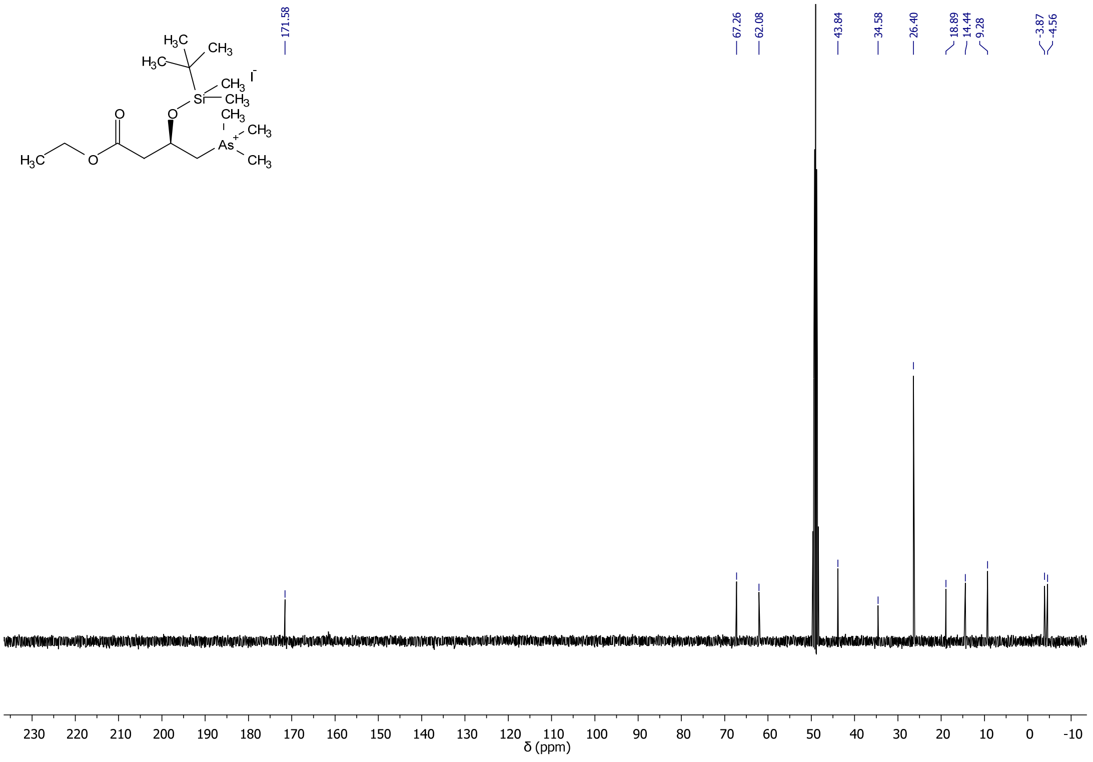

^{1}H NMR and ^{13}C NMR of (17)

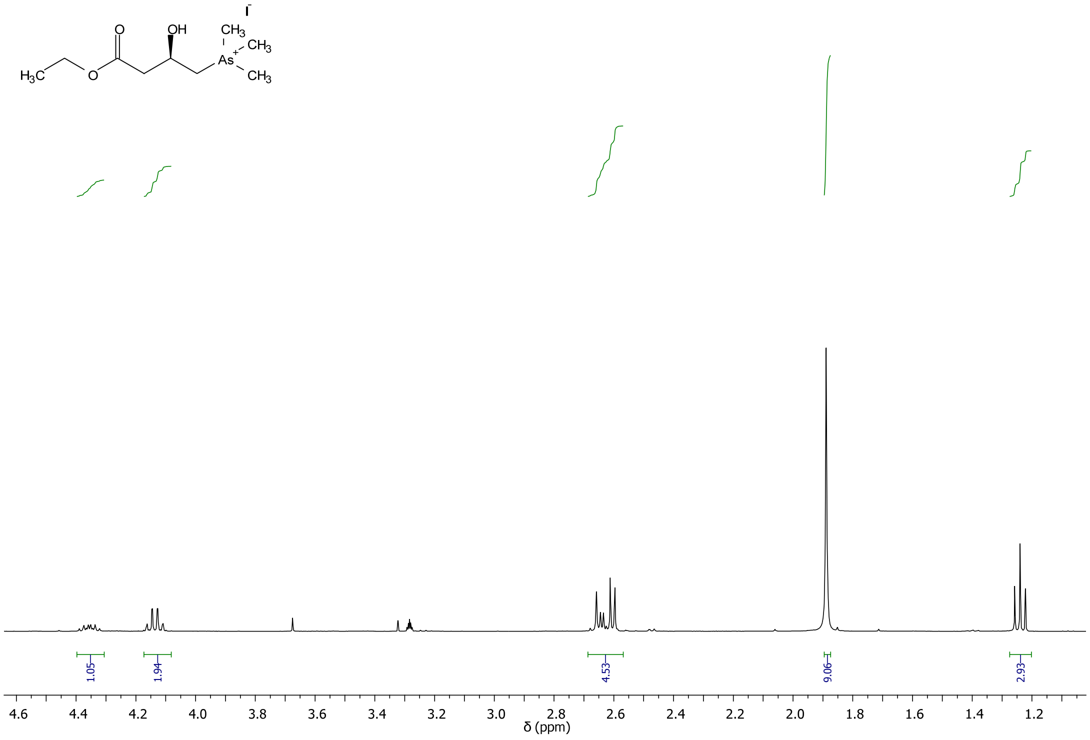

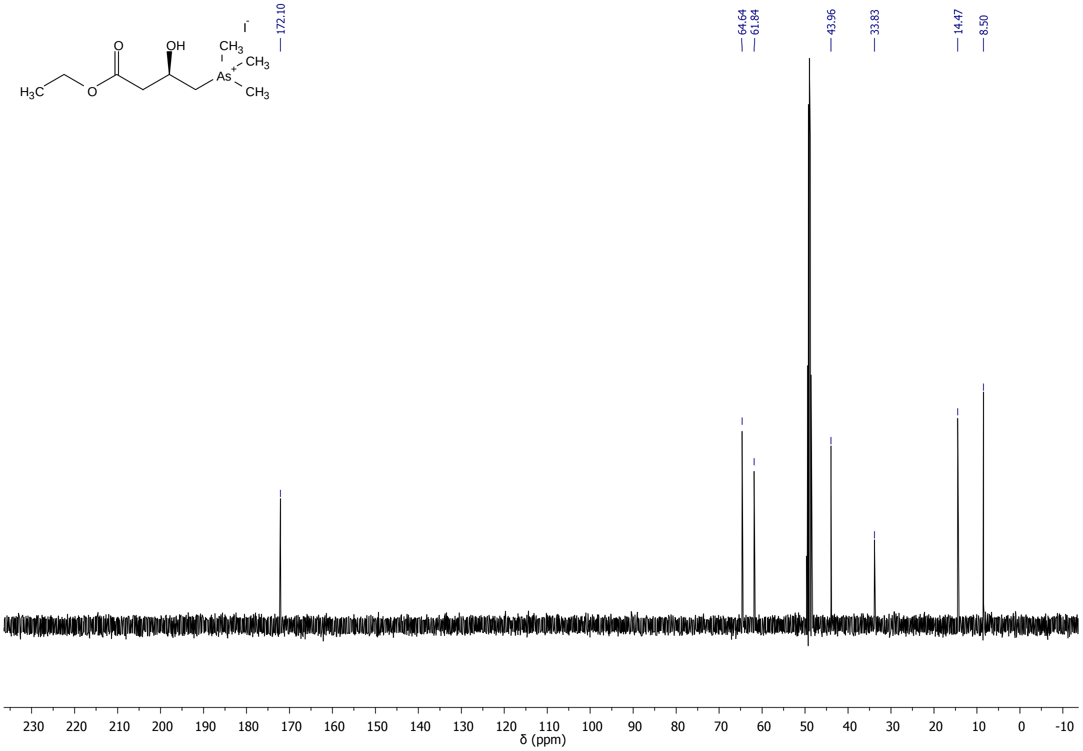

^{1}H NMR and ^{13}C NMR of (6)

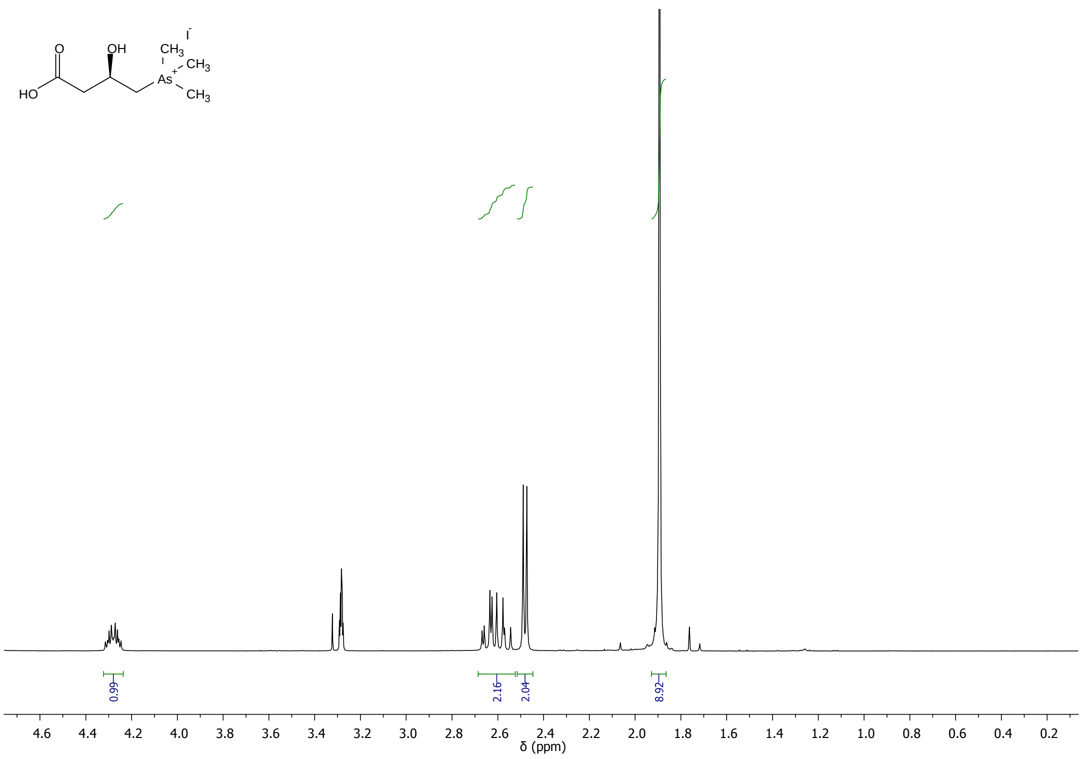

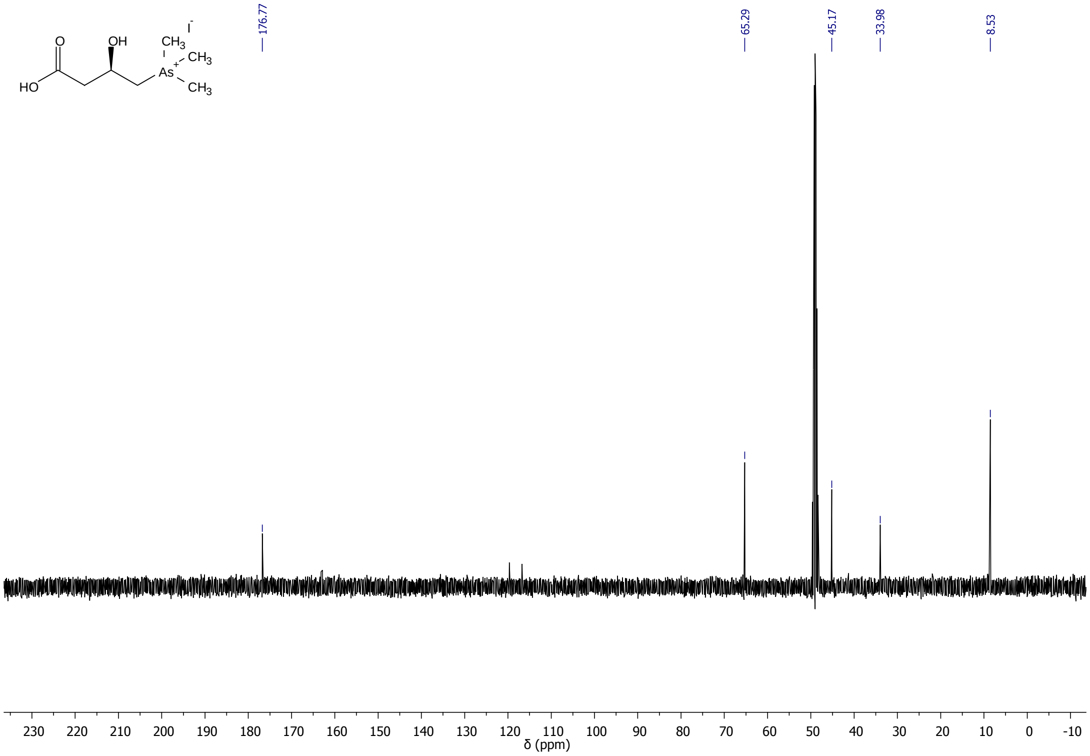

Peaks at 119 ppm derive from the TFA anion, which partially substitutes for the iodide ion.

^{1}H NMR and ^{13}C NMR of (18)

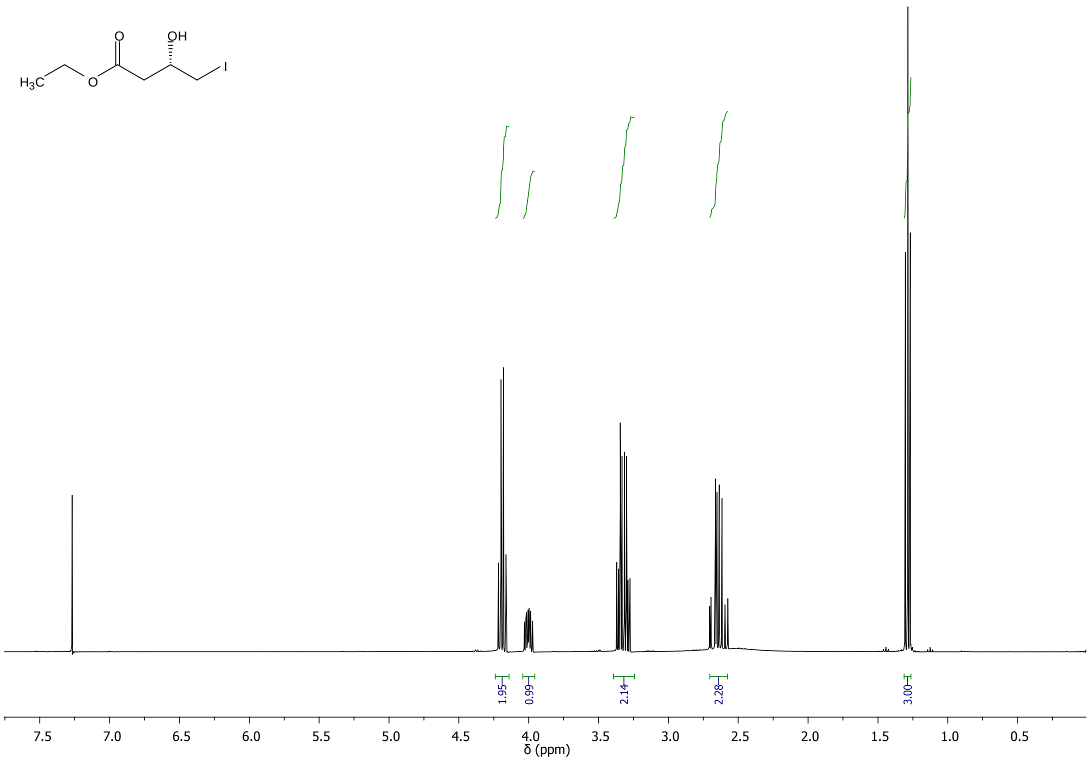

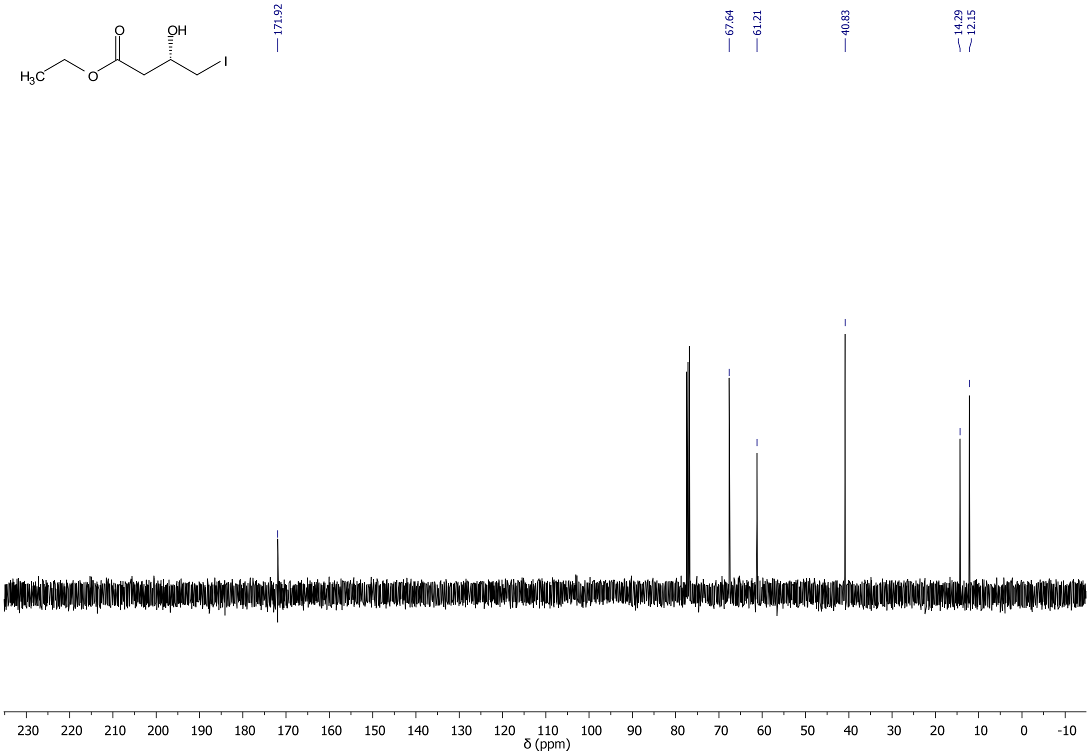

^{1}H NMR and ^{13}C NMR of (19)

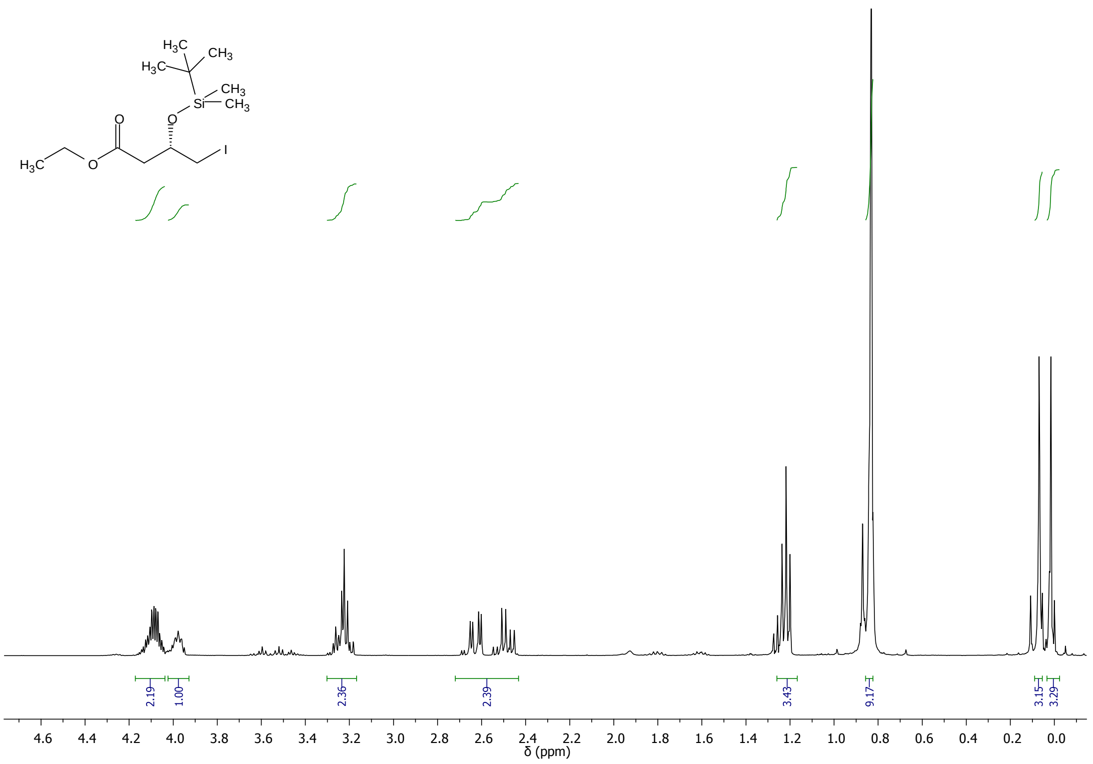

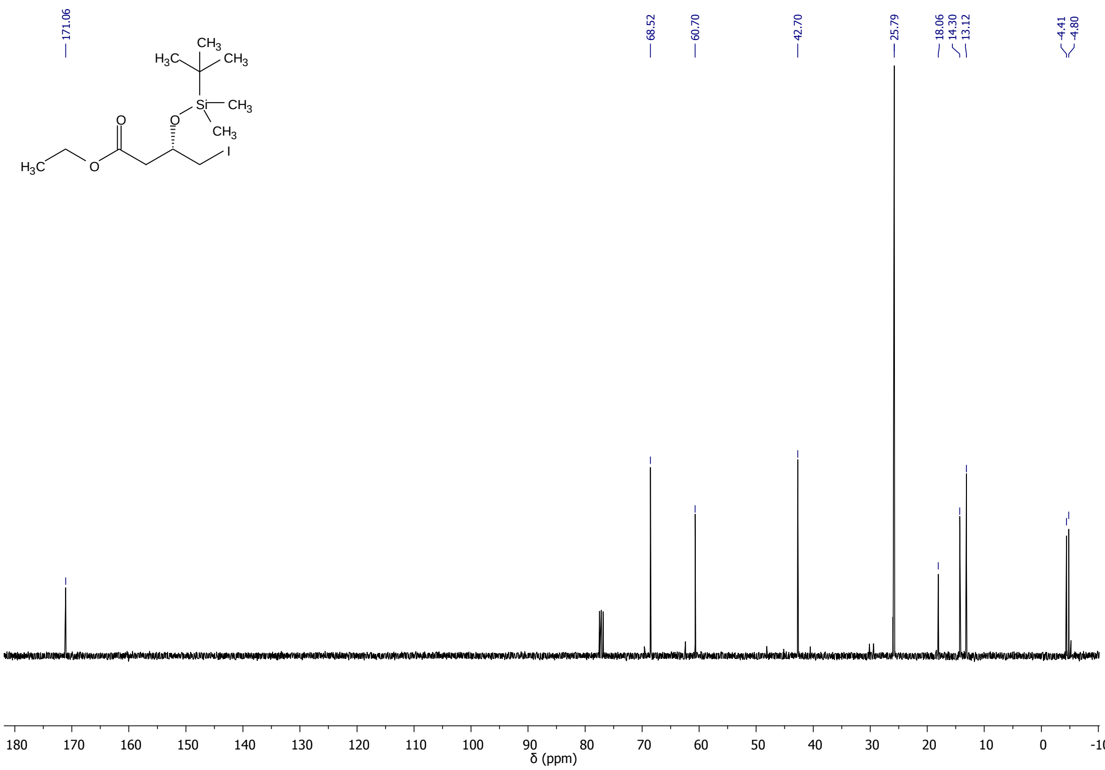

^{1}H NMR, ^{13}C NMR and ^{31}P NMR of (20)

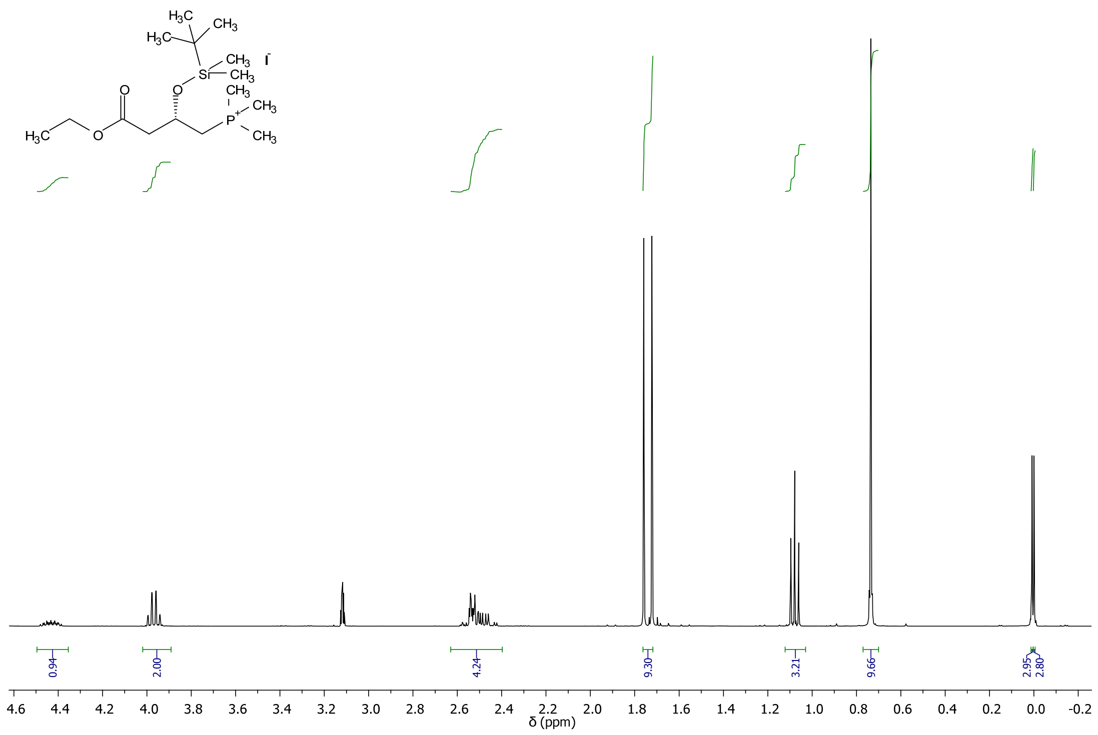

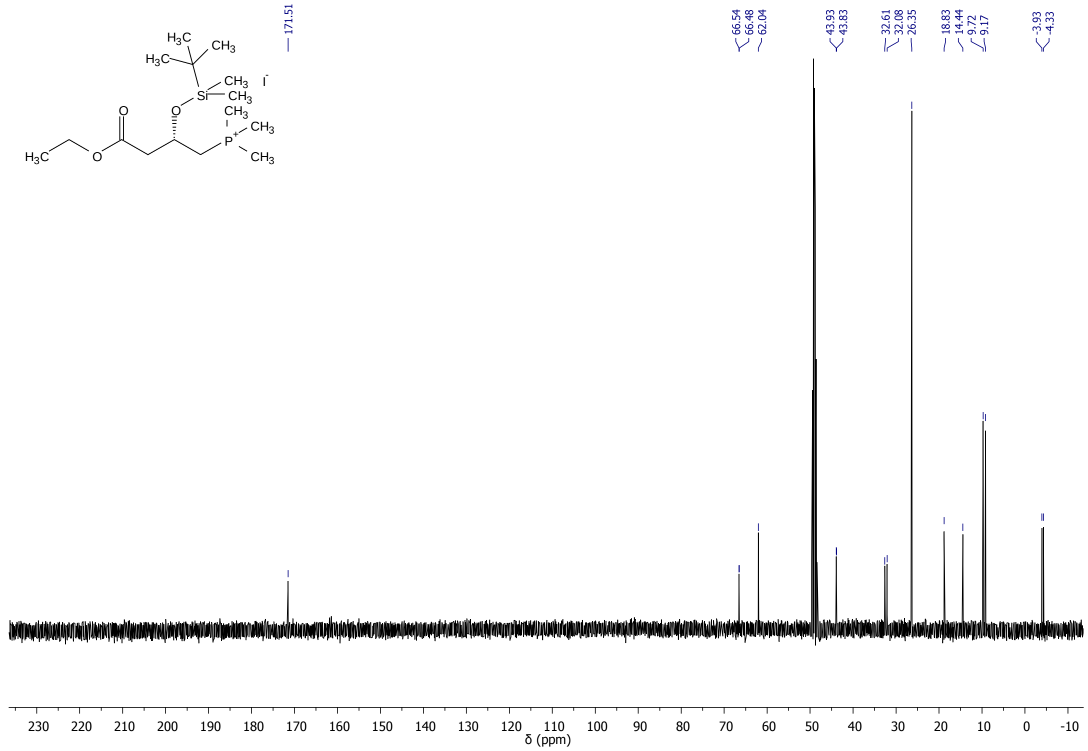

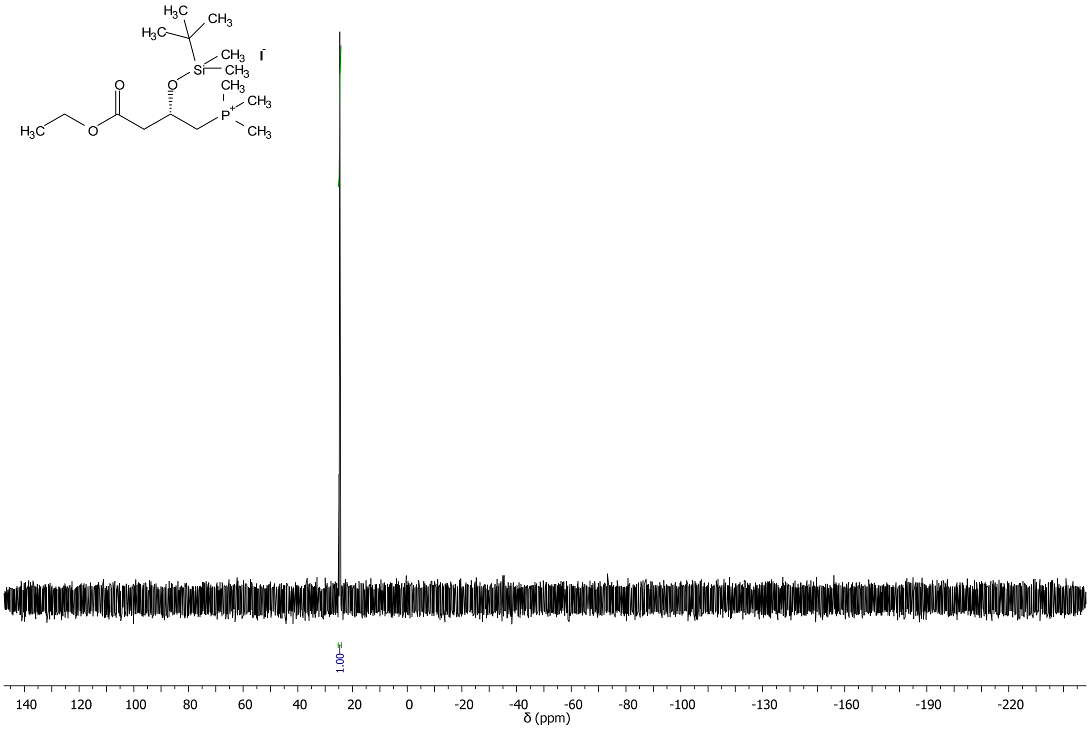
1.00
140
120
100
80
60
40
20
0
-20
-40
-60
-80
-100
-130
-160
-190
-220
δ (ppm)

^{1}H NMR, ^{13}C NMR and ^{31}P NMR of (21)

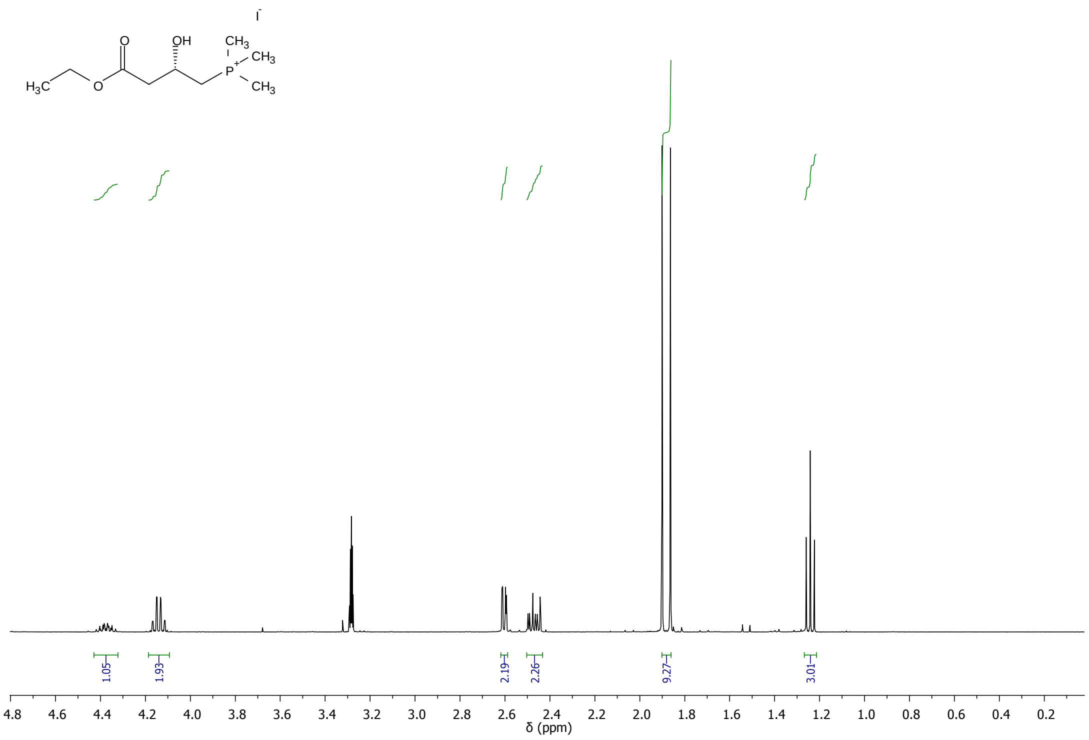

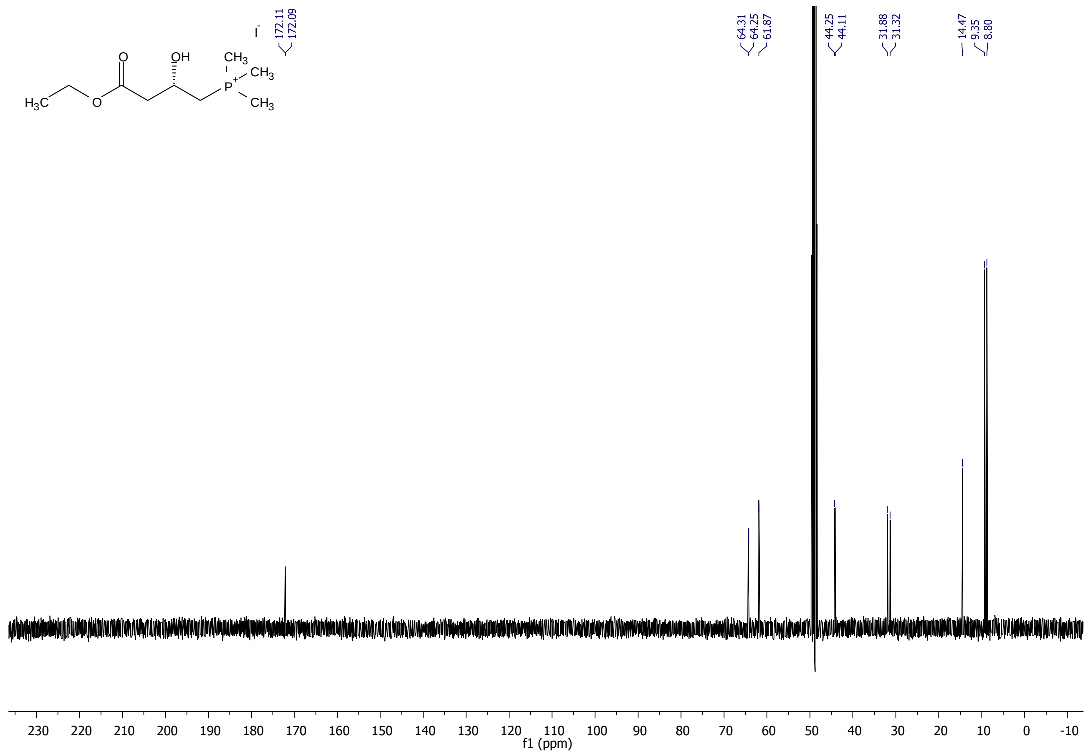

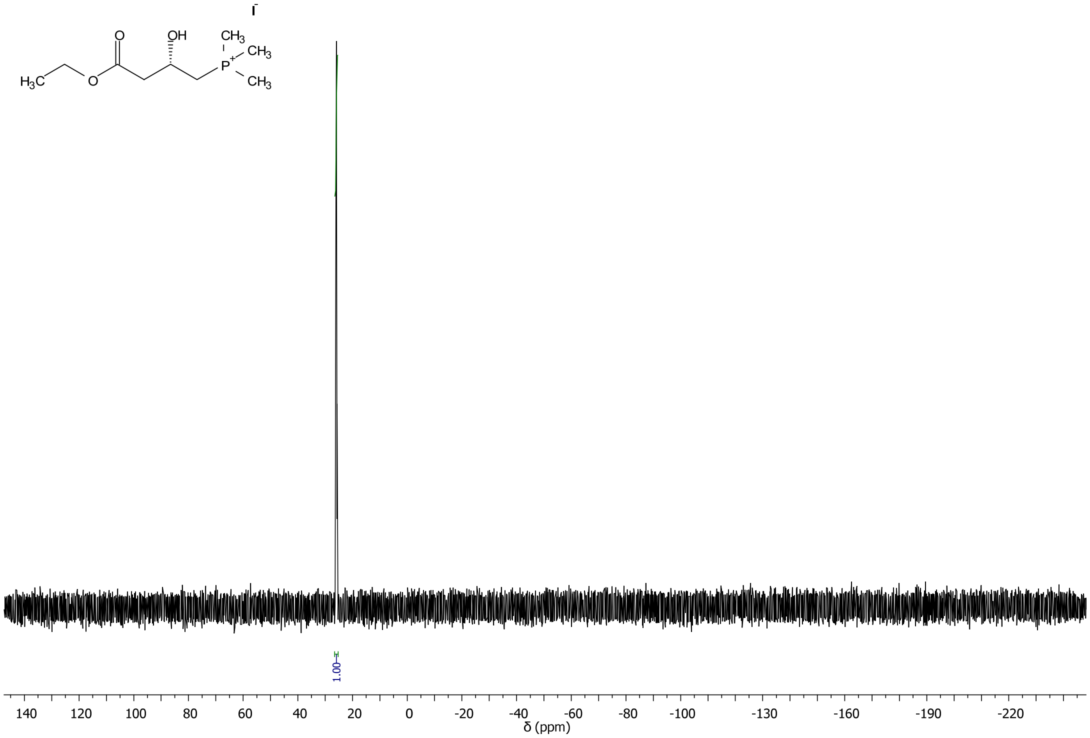

I⁻
O
OH
CH3
CH3
H3C
O
P+
CH3
1.00
140
120
100
80
60
40
20
0
-20
-40
-60
-80
-100
-130
-160
-190
-220
δ (ppm)

^{1}H NMR, ^{13}C NMR and ^{31}P NMR of (7)

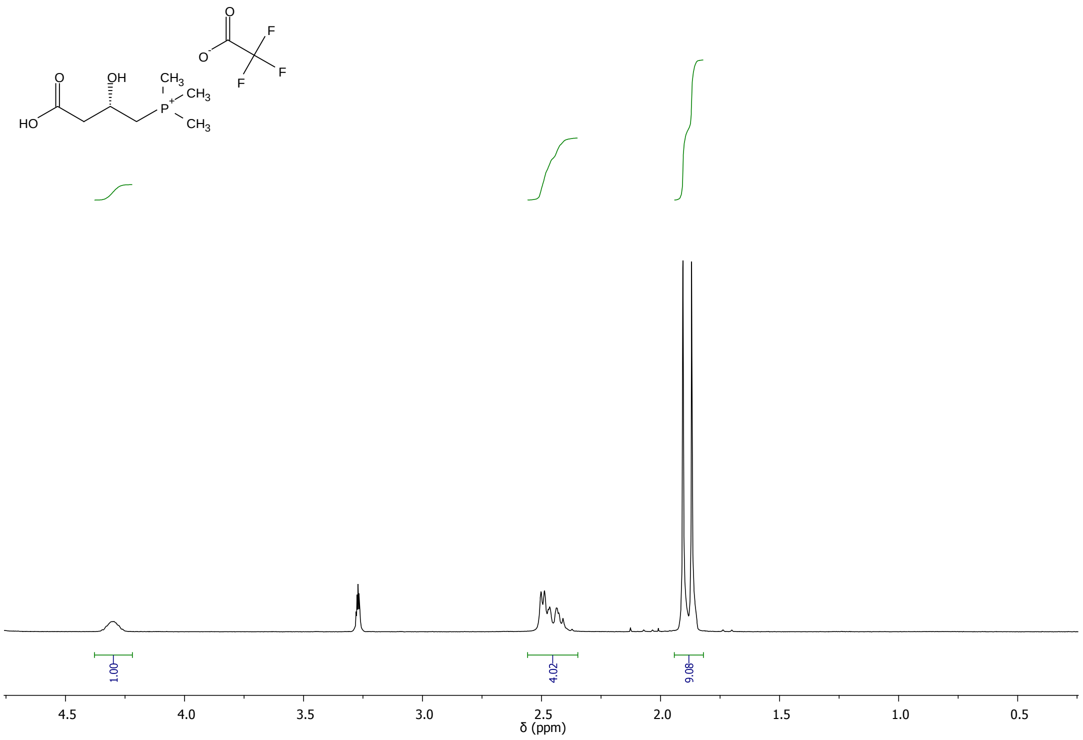

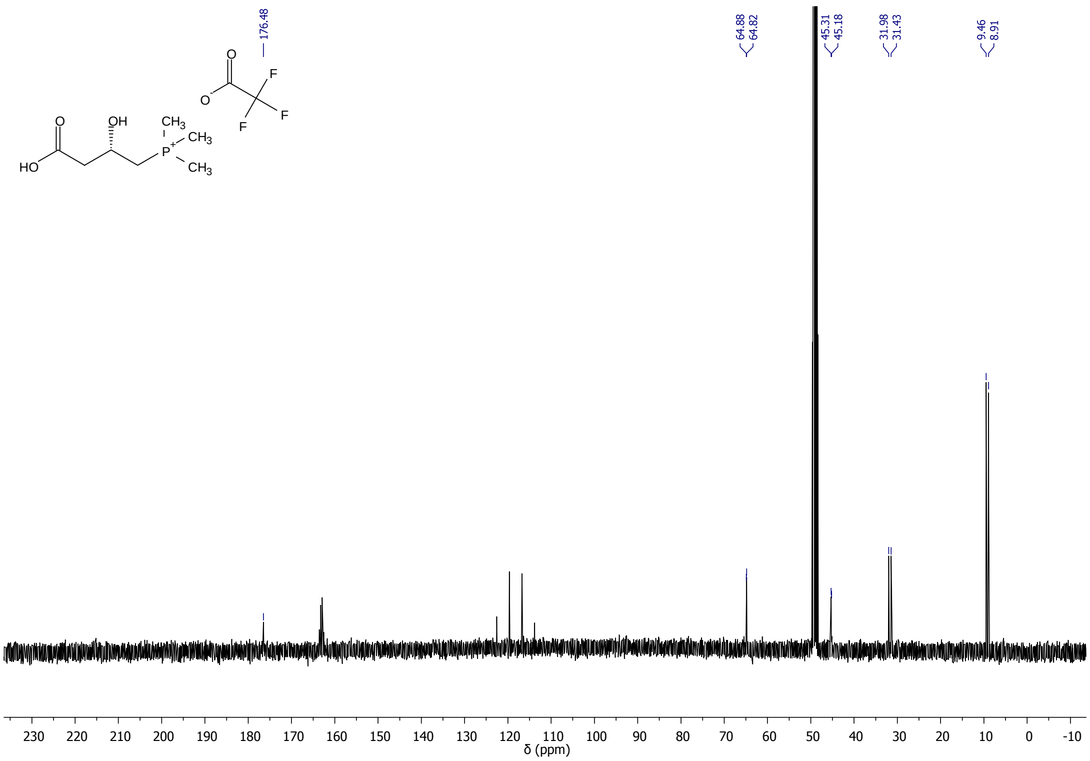

Peaks at 163 and 119 ppm derive from the TFA anion.

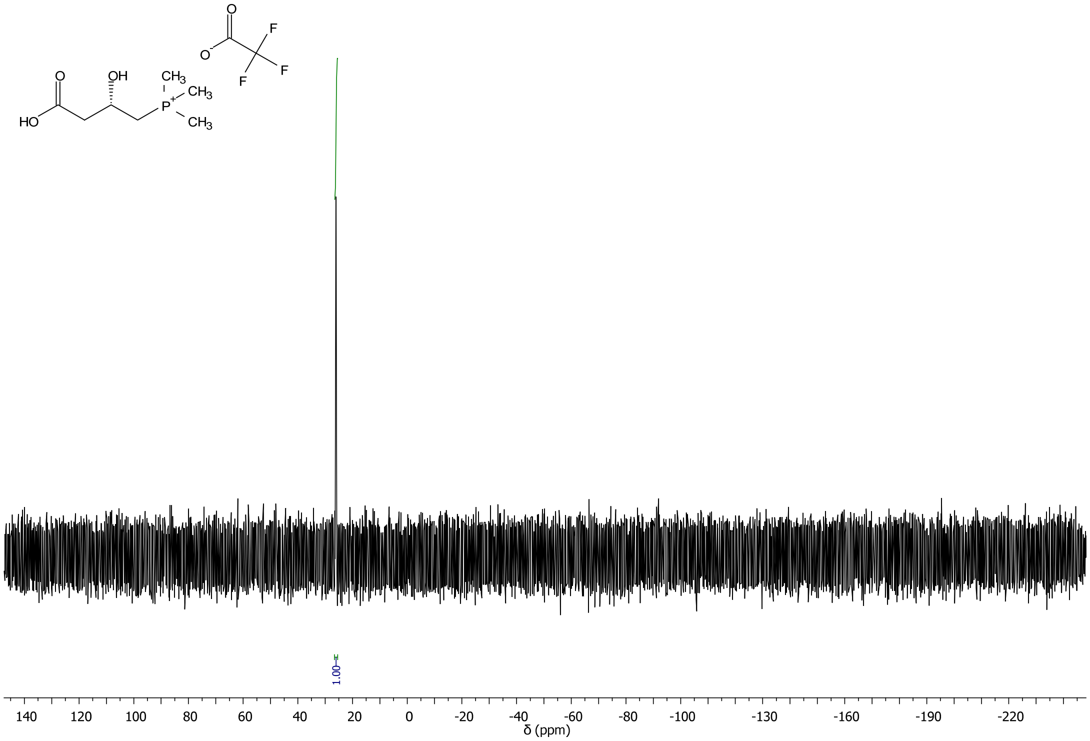
1.00
140 120 100 80 60 40 20 0 -20 -40 -60 -80 -100 -130 -160 -190 -220
δ (ppm)

^{1}H NMR and ^{13}C NMR of (22)

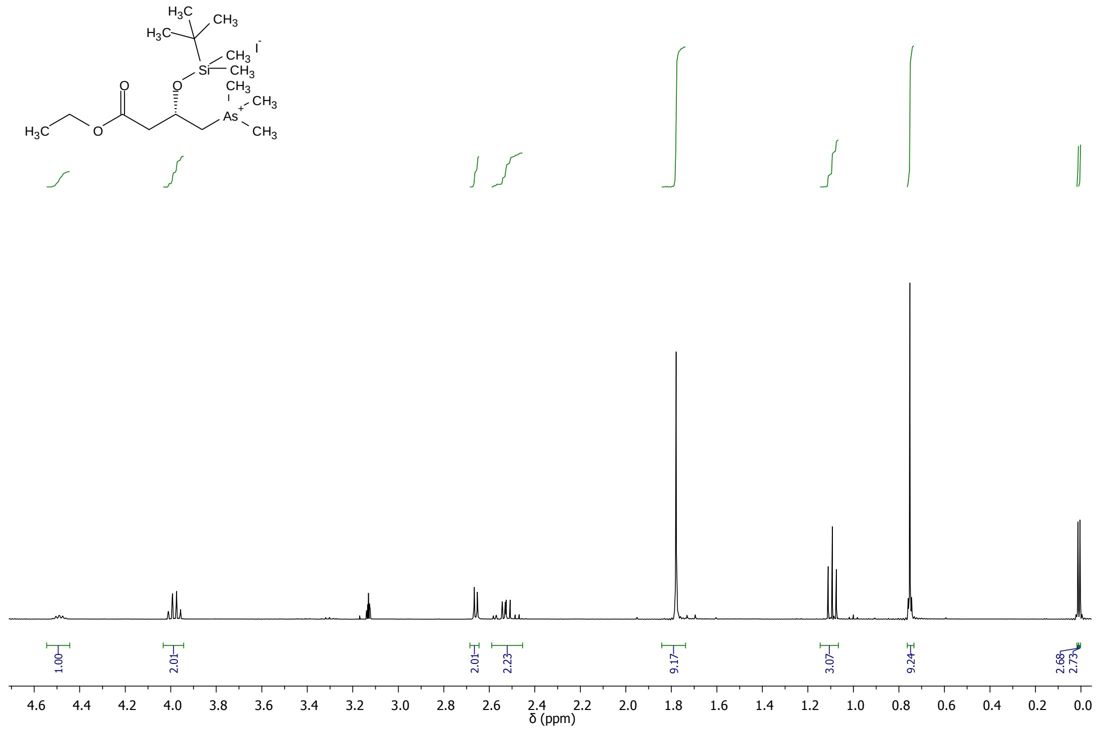

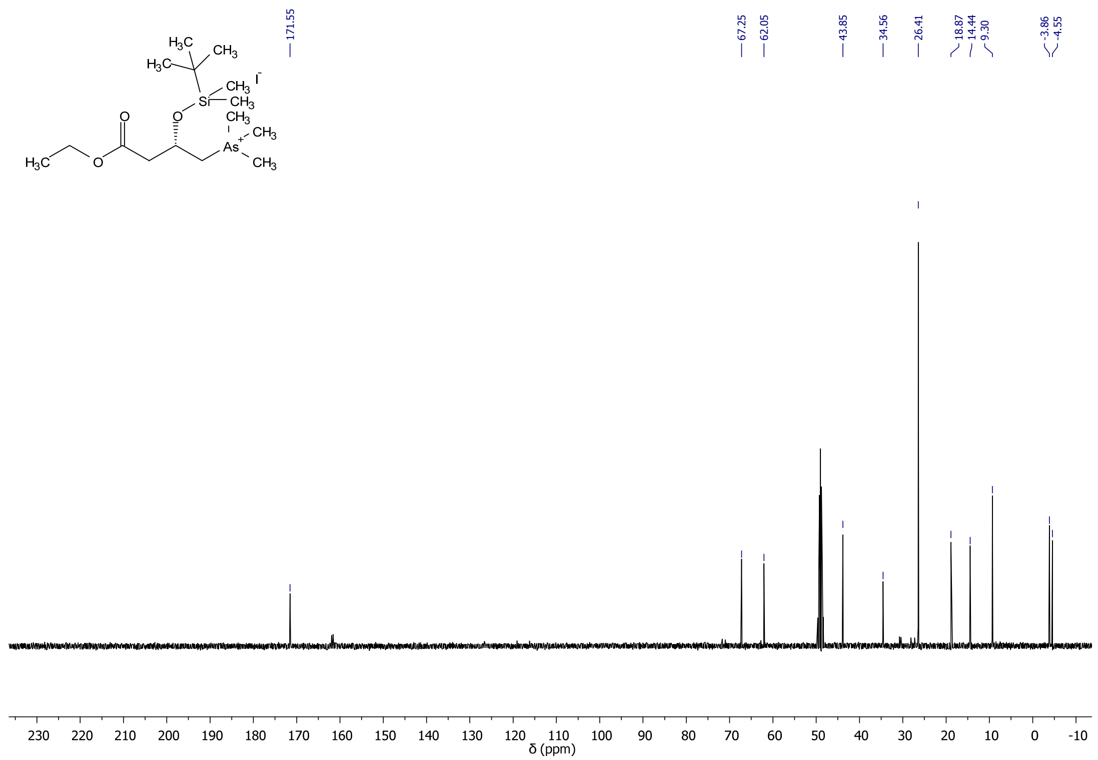

^{1}H NMR and ^{13}C NMR of (23)

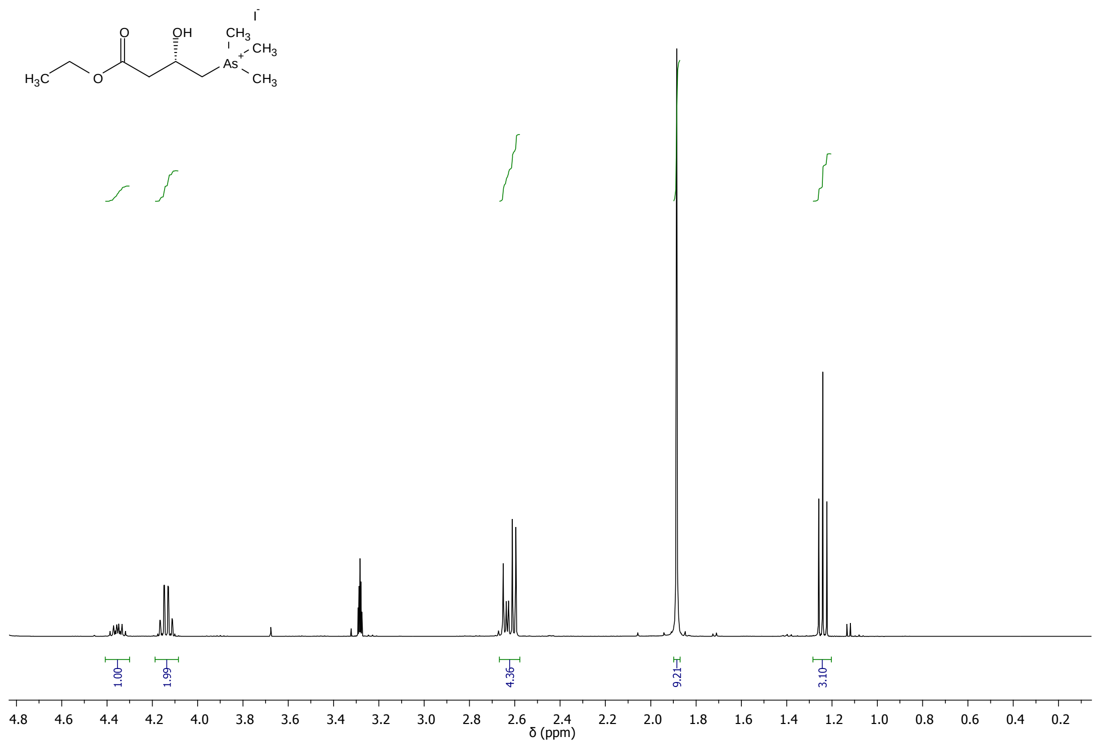

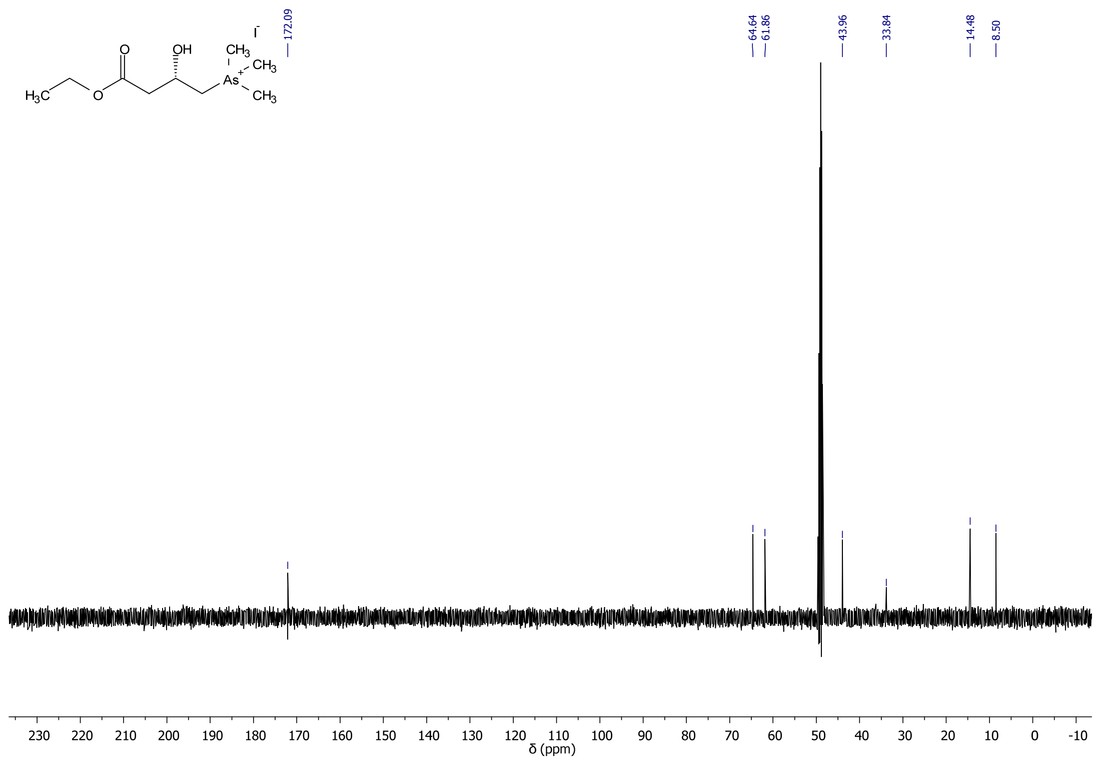

^{1}H NMR and ^{13}C NMR of (8)

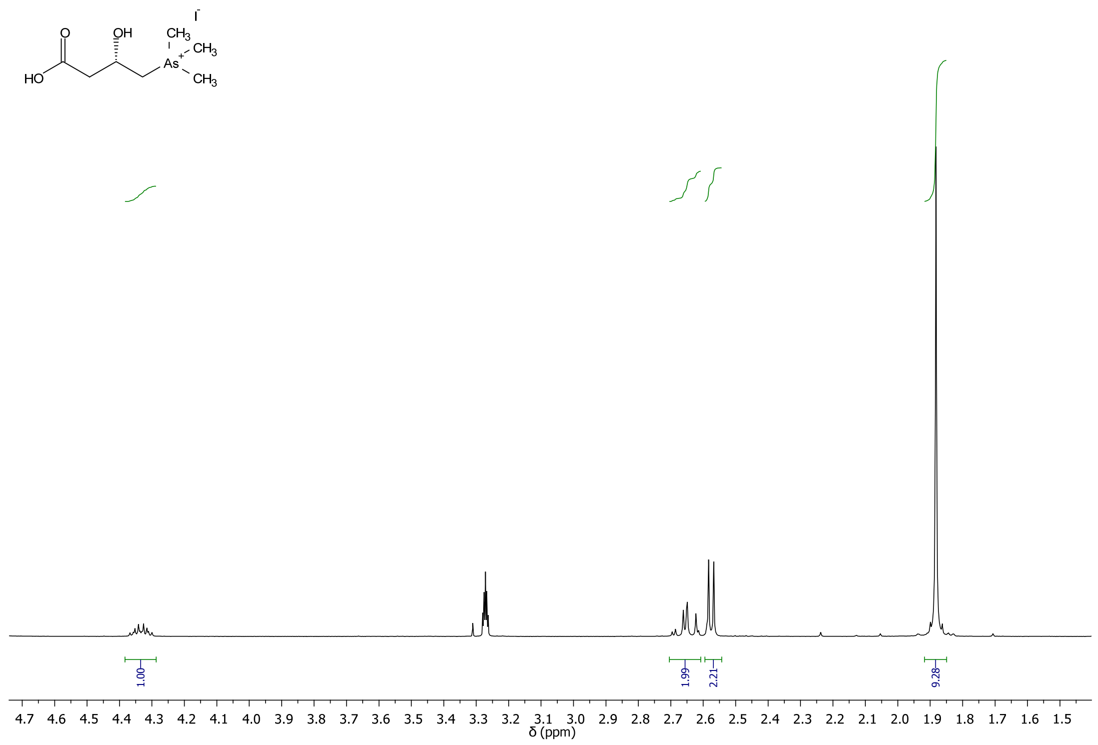

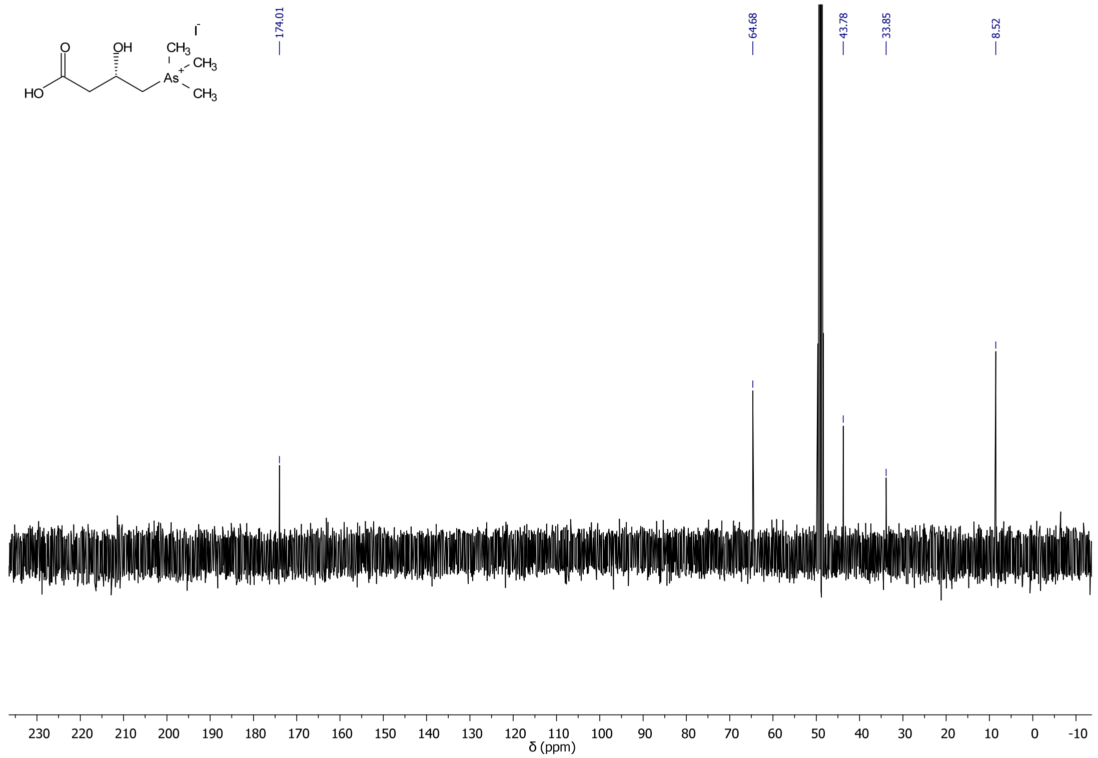

4. References

1. D. L. Ziering, R. A. Pascal, *J. Am. Chem. Soc.* **1990**, *112*, 834–838.

2. G. P. J. Hareau, M. Koiwa, S. Hikichi, F. Sato, *J. Am. Chem. Soc.* **1999**, *121*, 3640-3650.

3. A. M. Rydzik, I. K. H. Leung, G. T. Kochan, N. D. Loik, L. Henry, M. A. McDonough, T. D. W. Claridge, C. J. Schofield, *Org. Biomol. Chem.* **2014**, *12*, 6354-6358.

4. F. Martin, H. Zipse, *J. Comput. Chem.* **2005**, *26*, 97-105.
